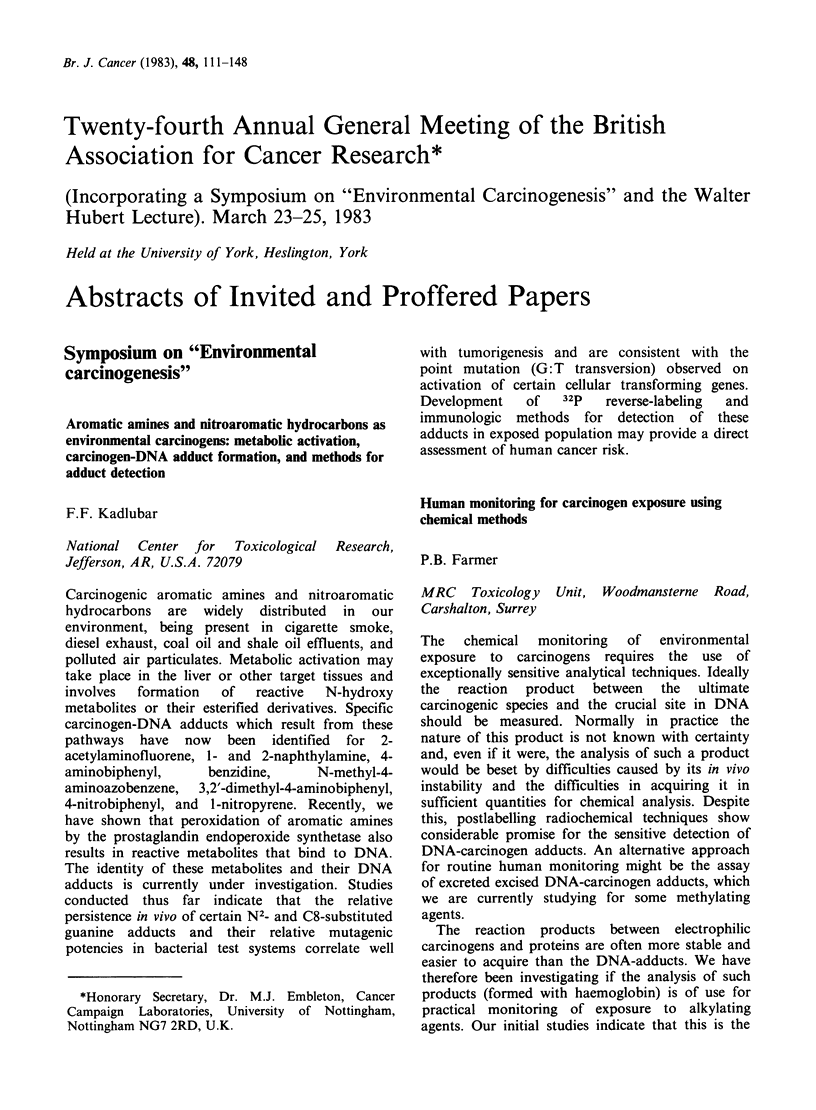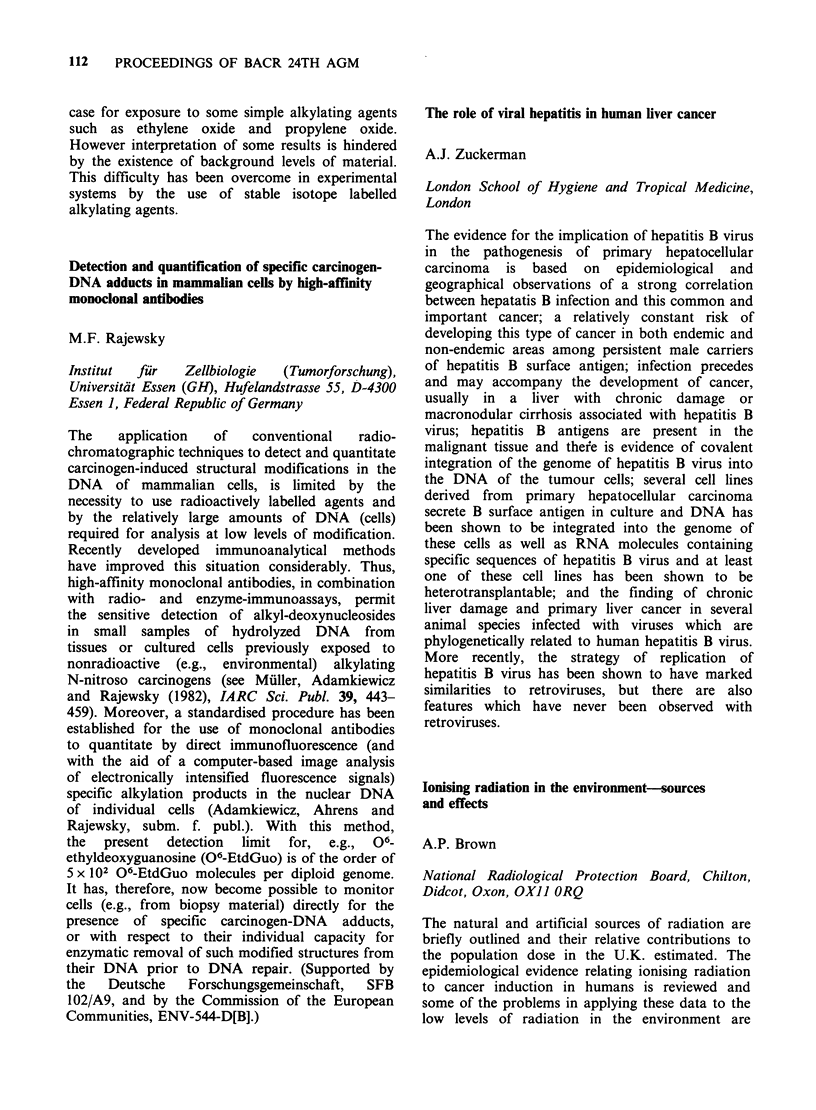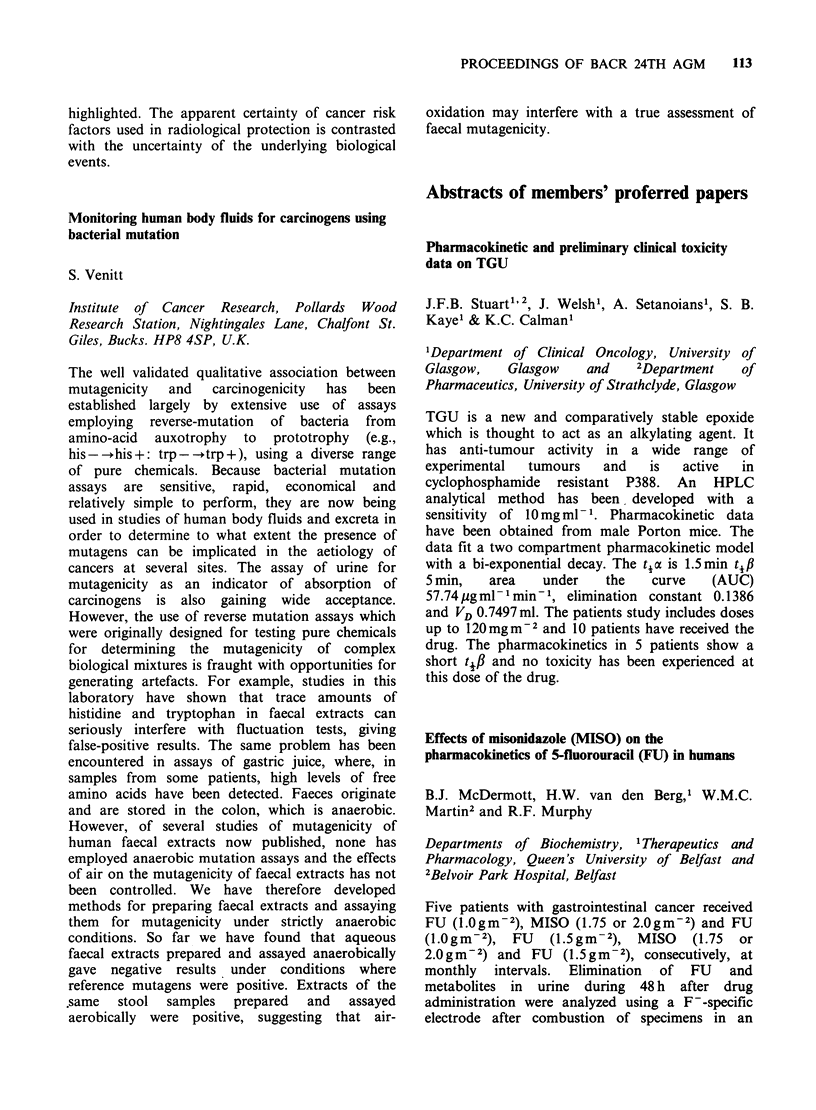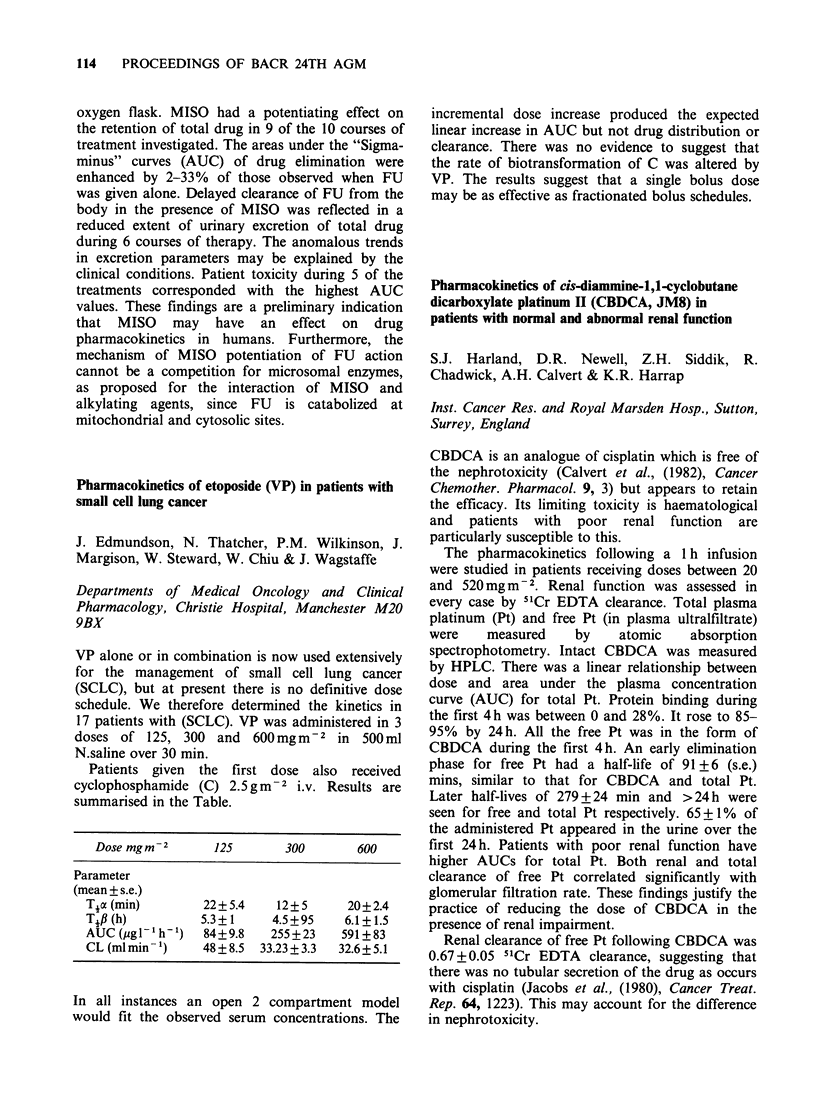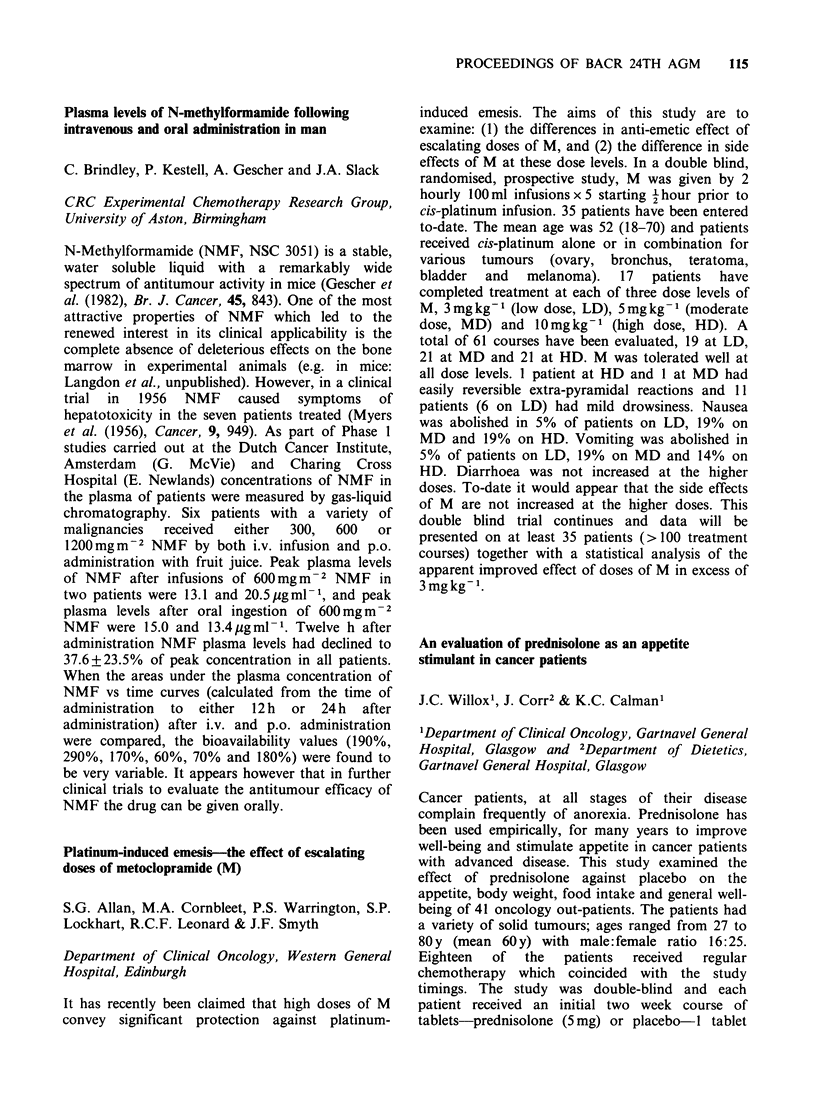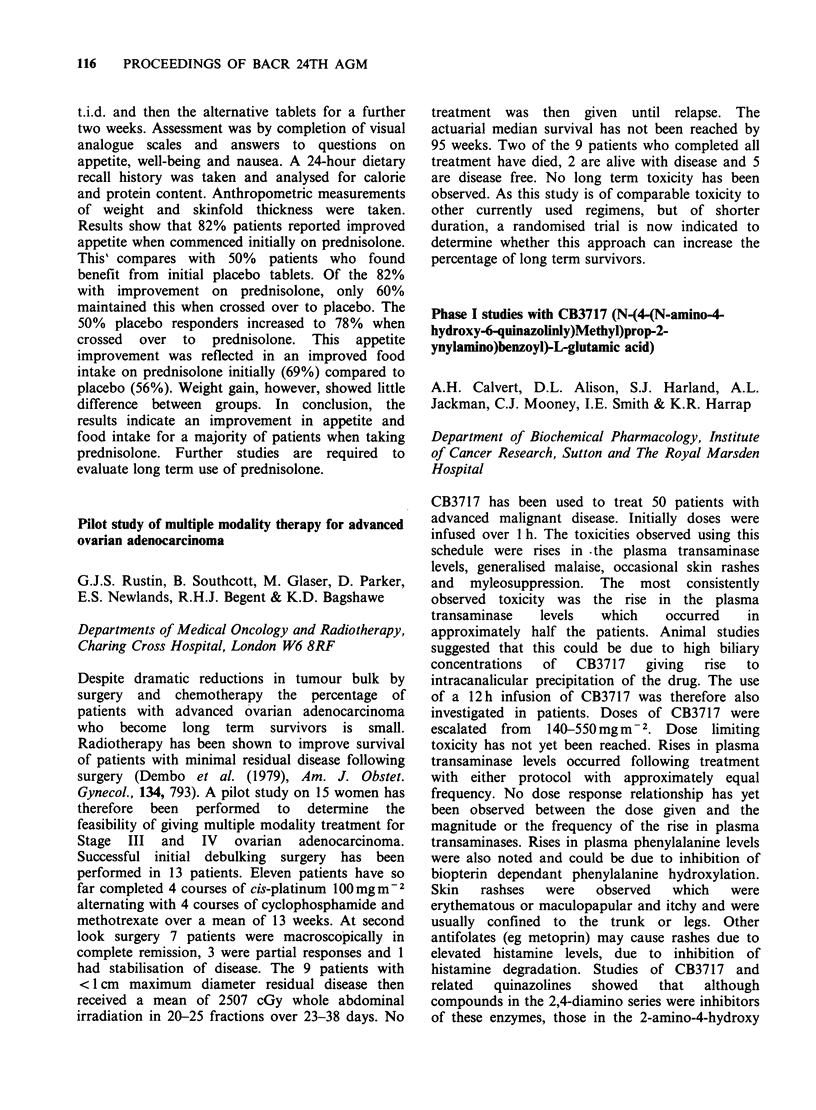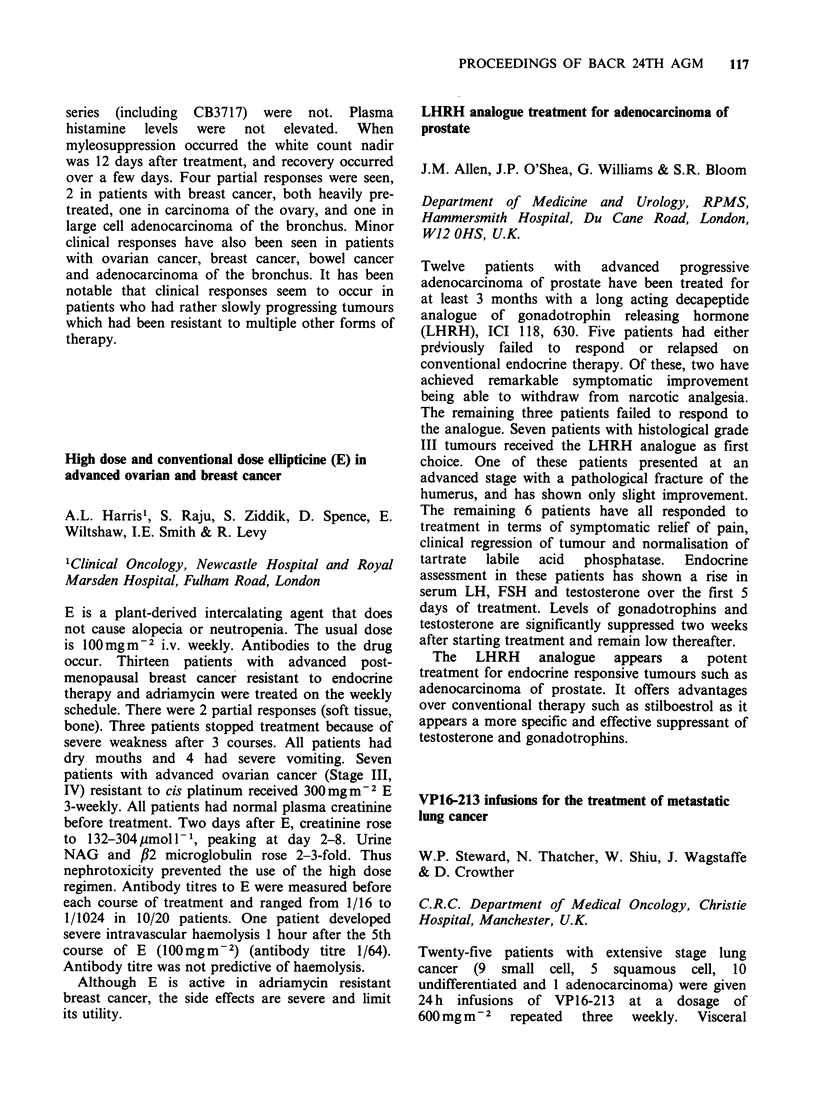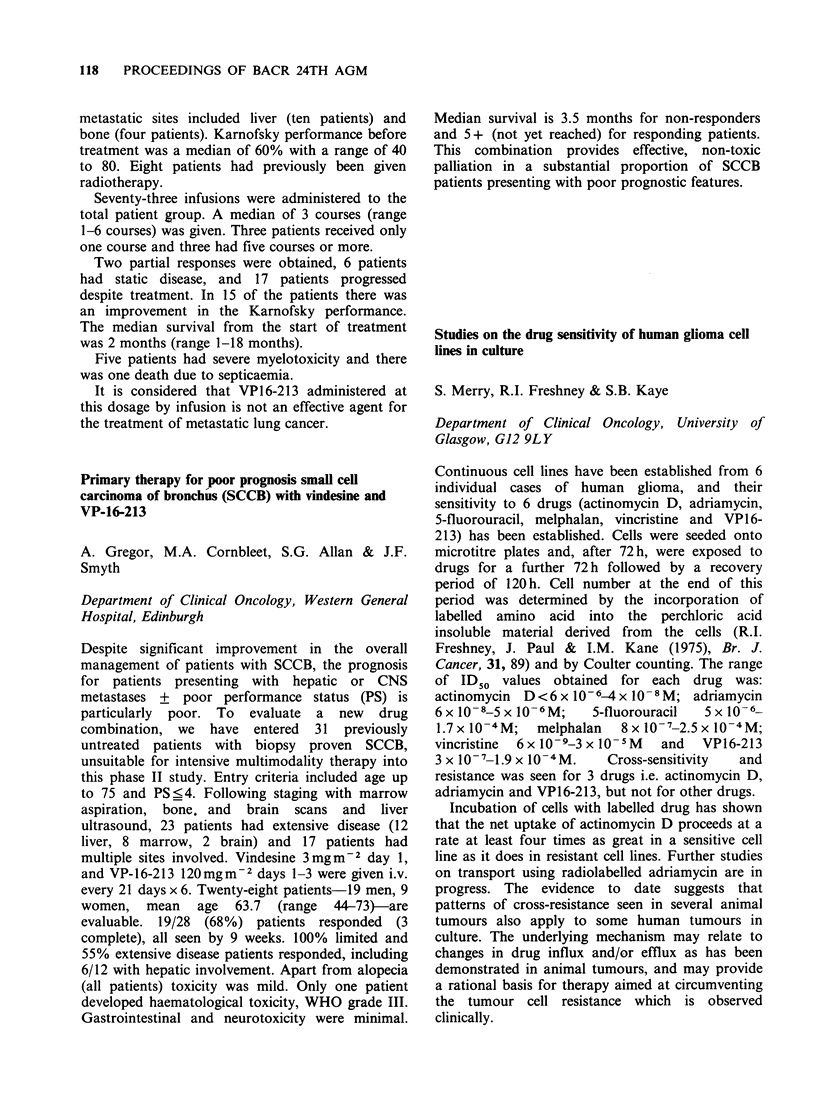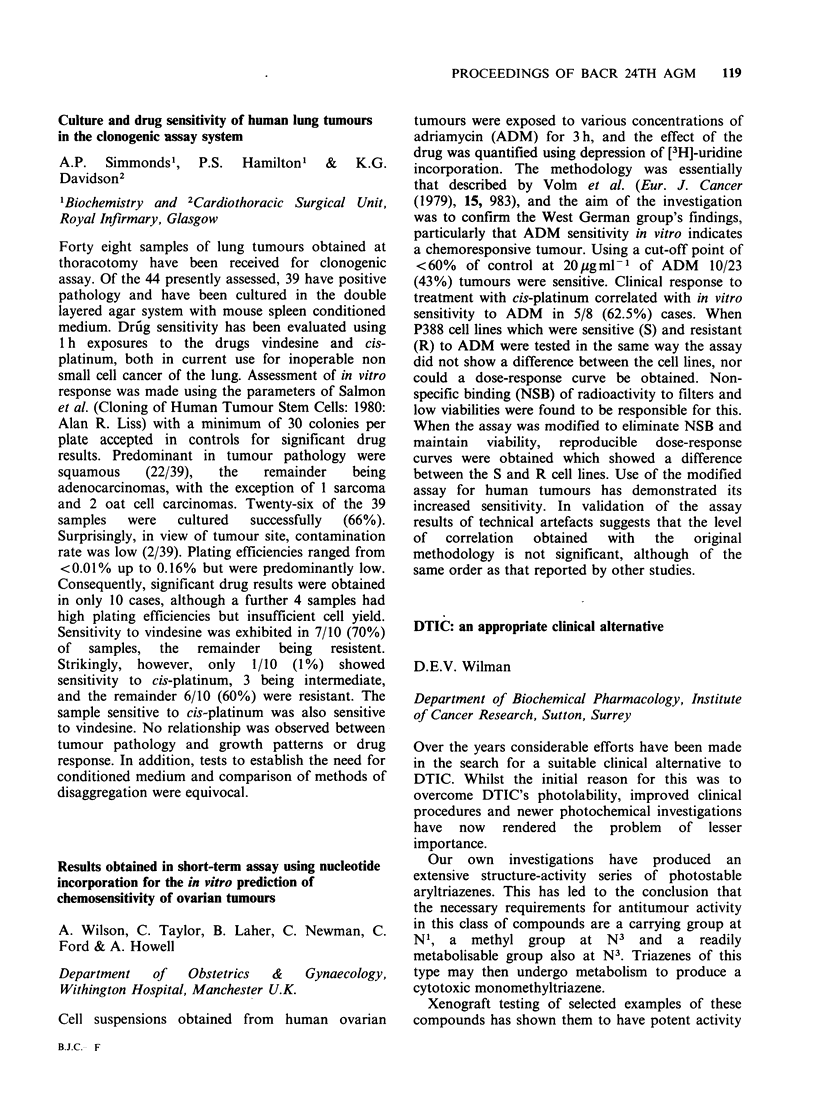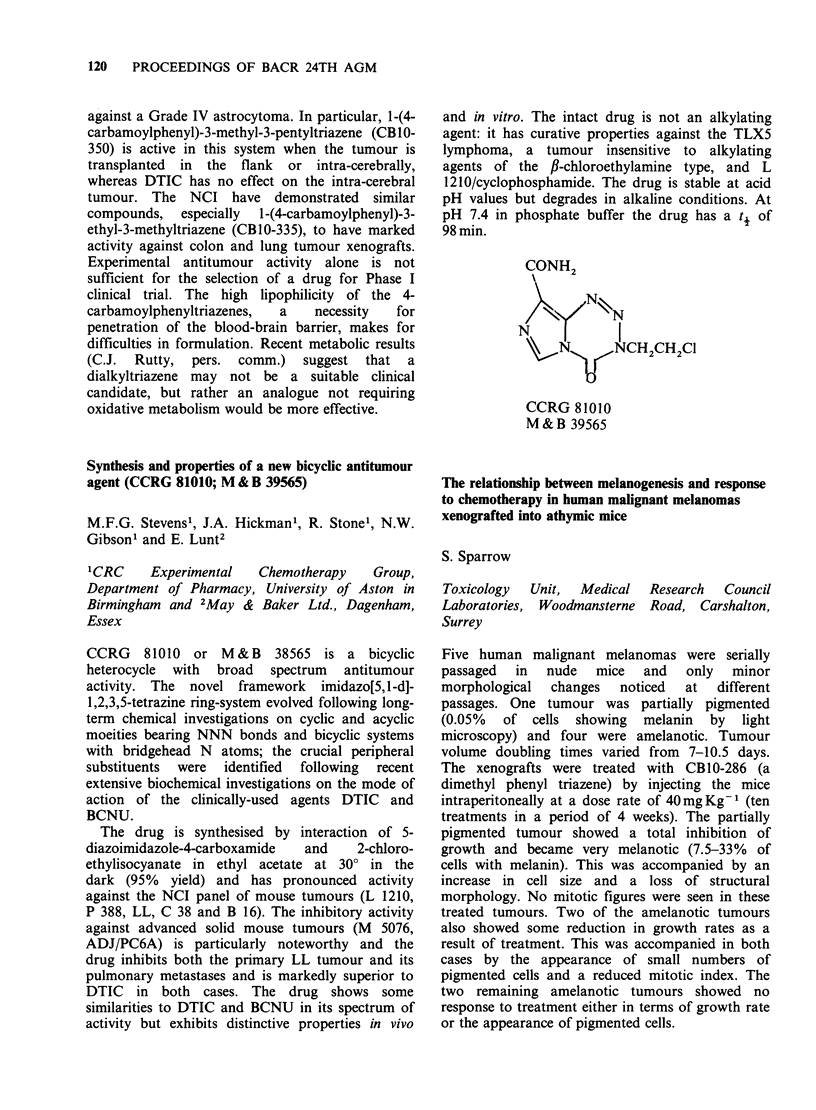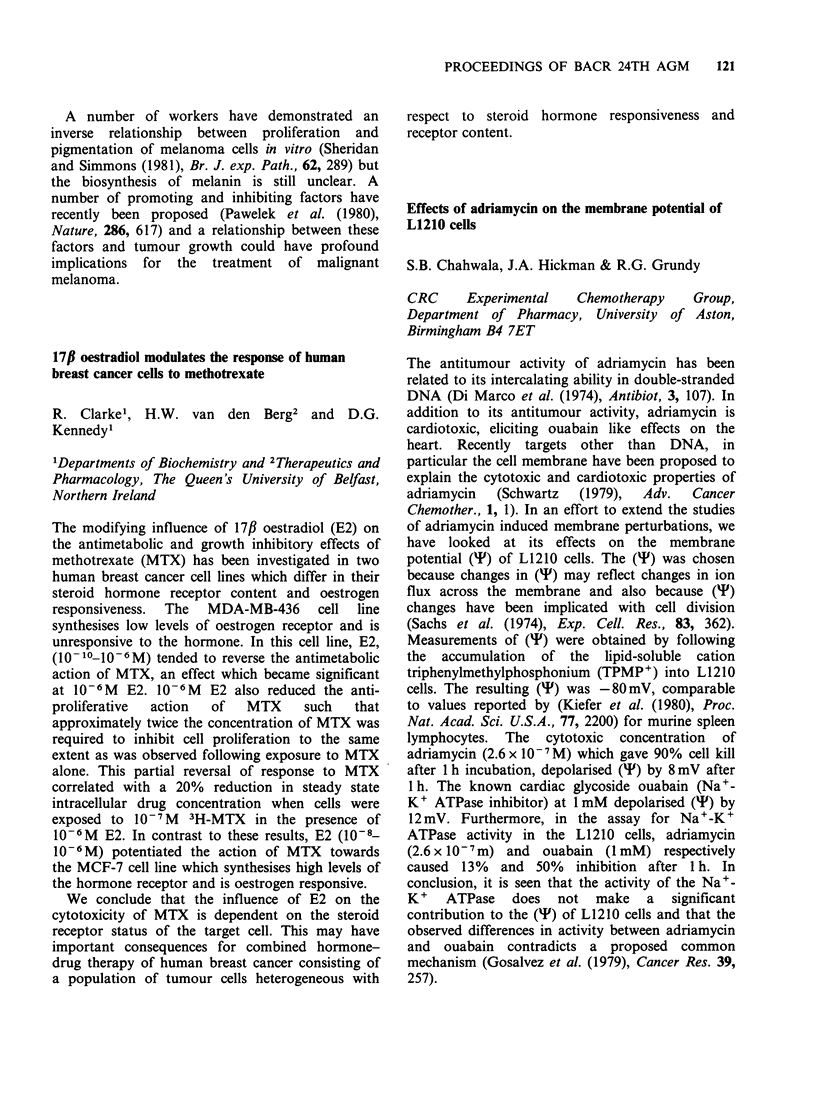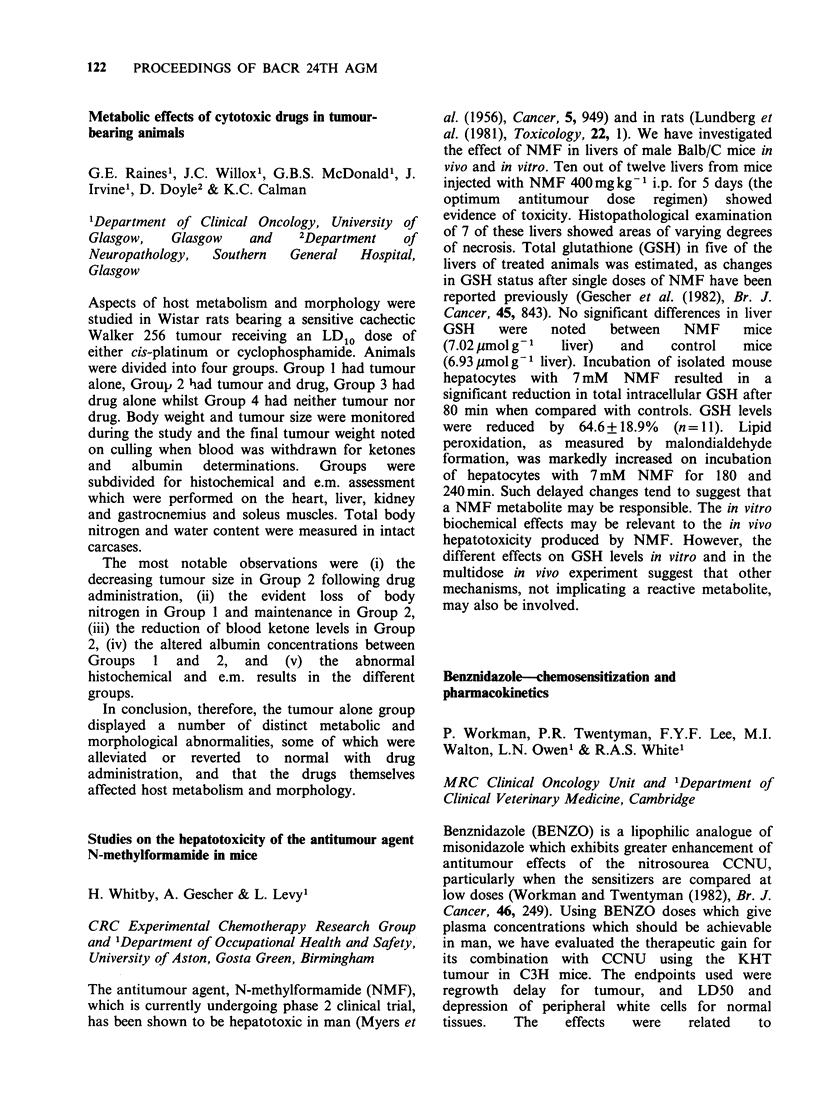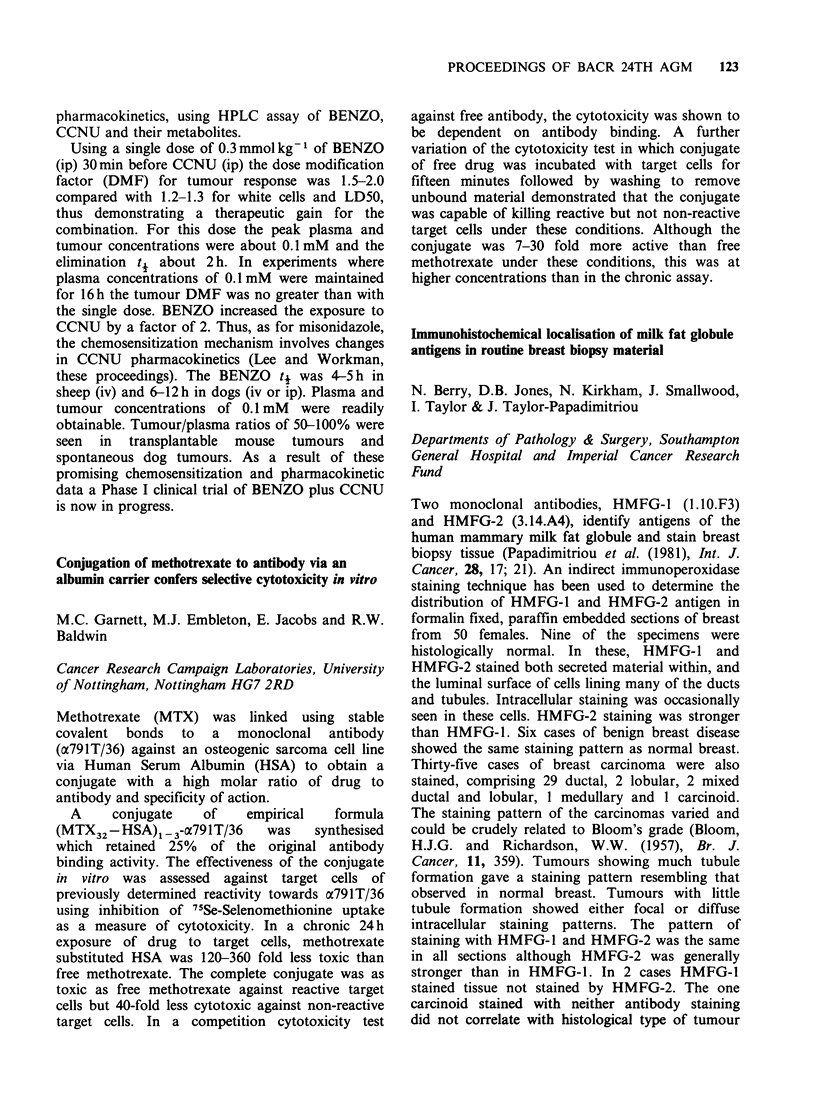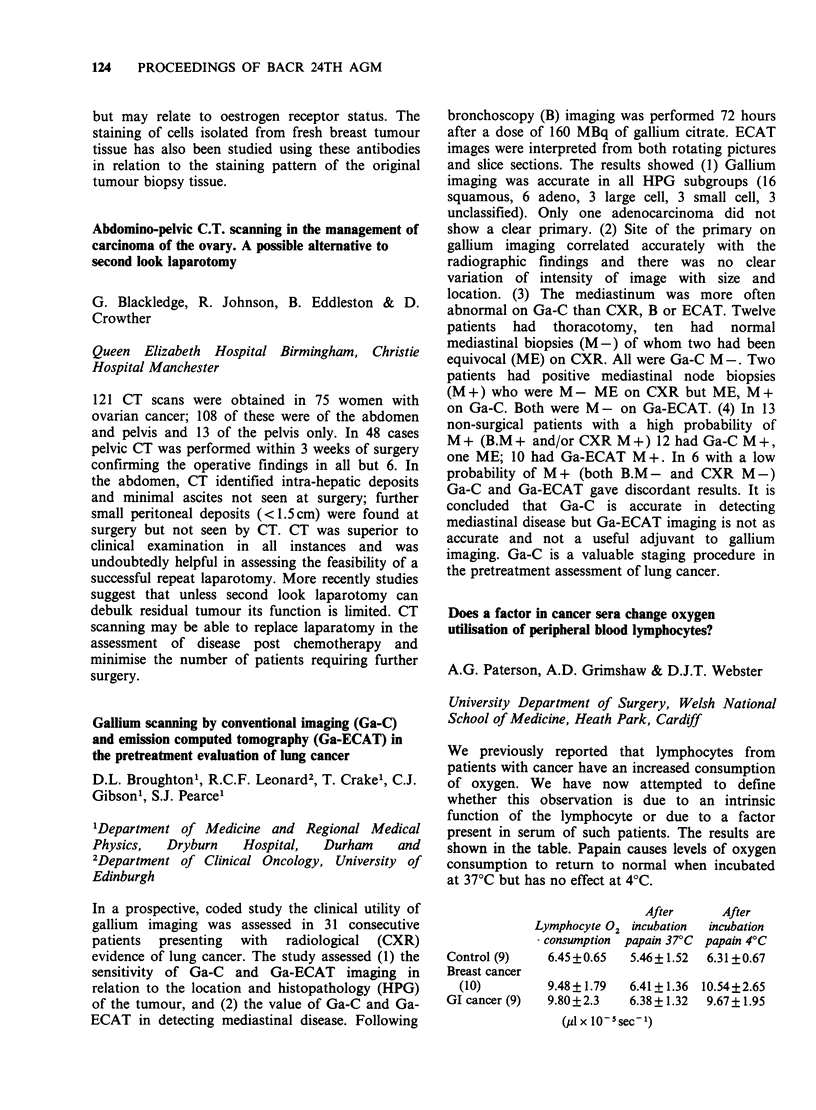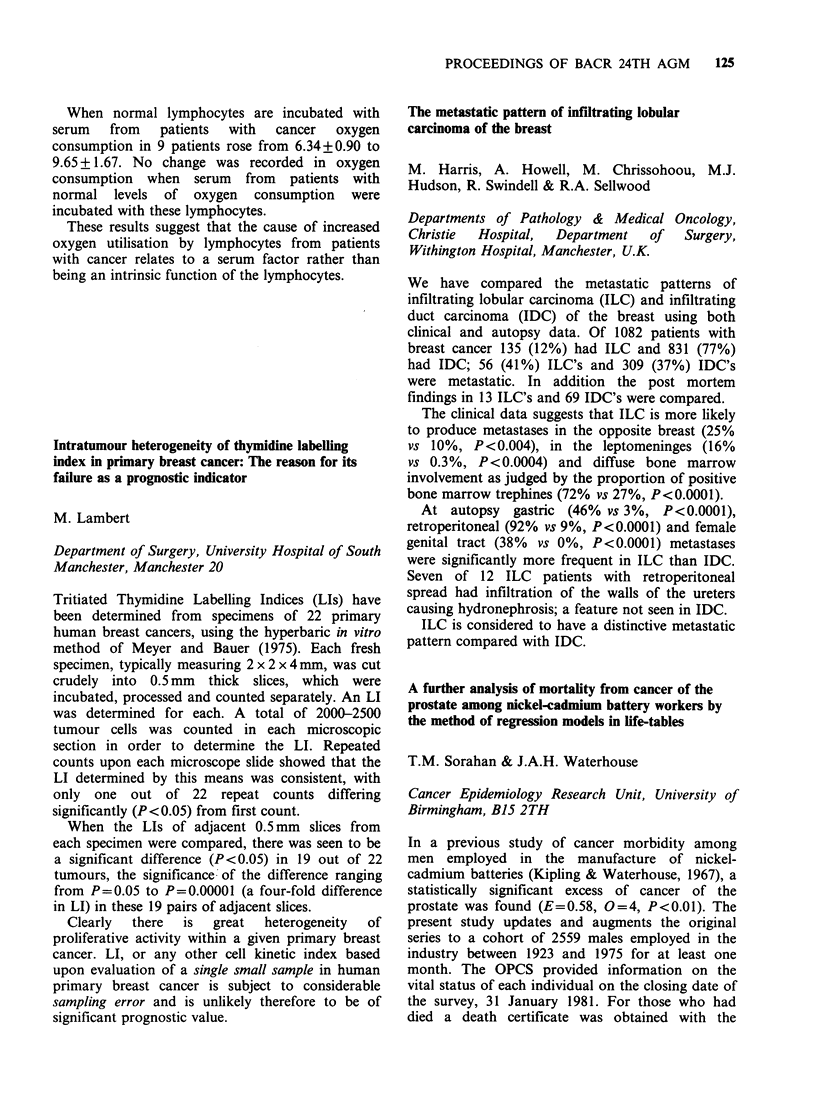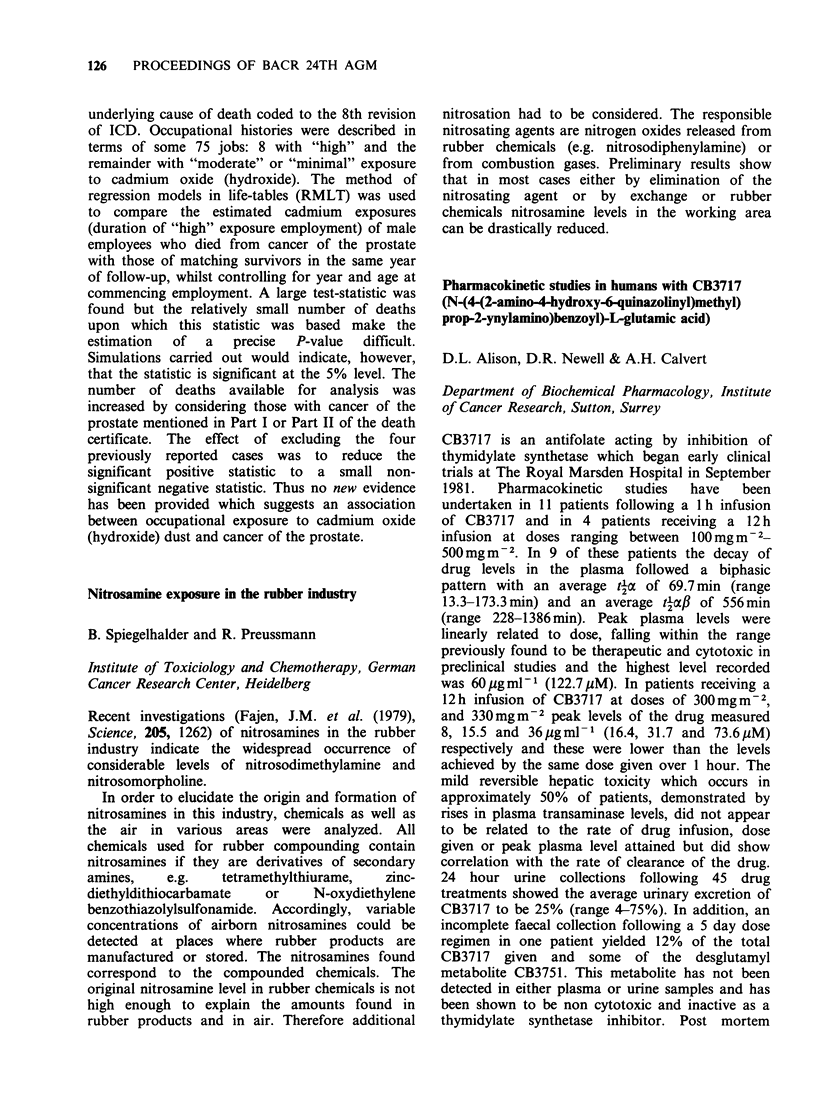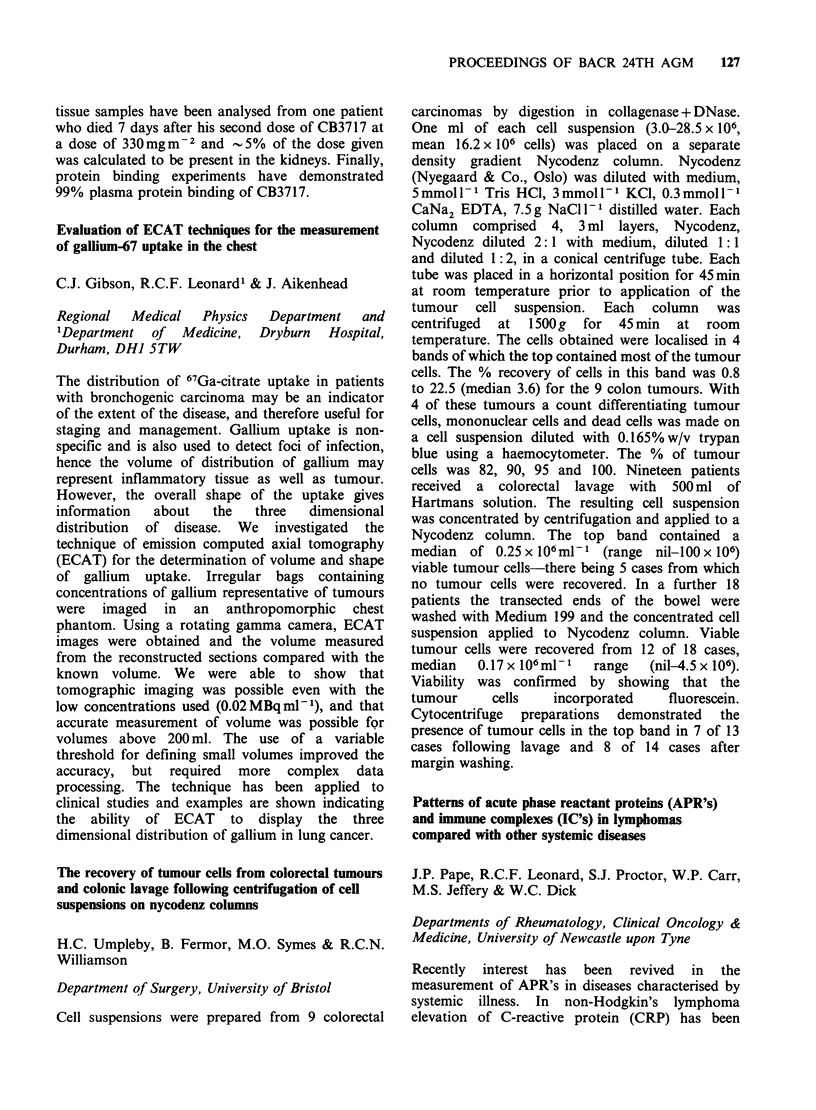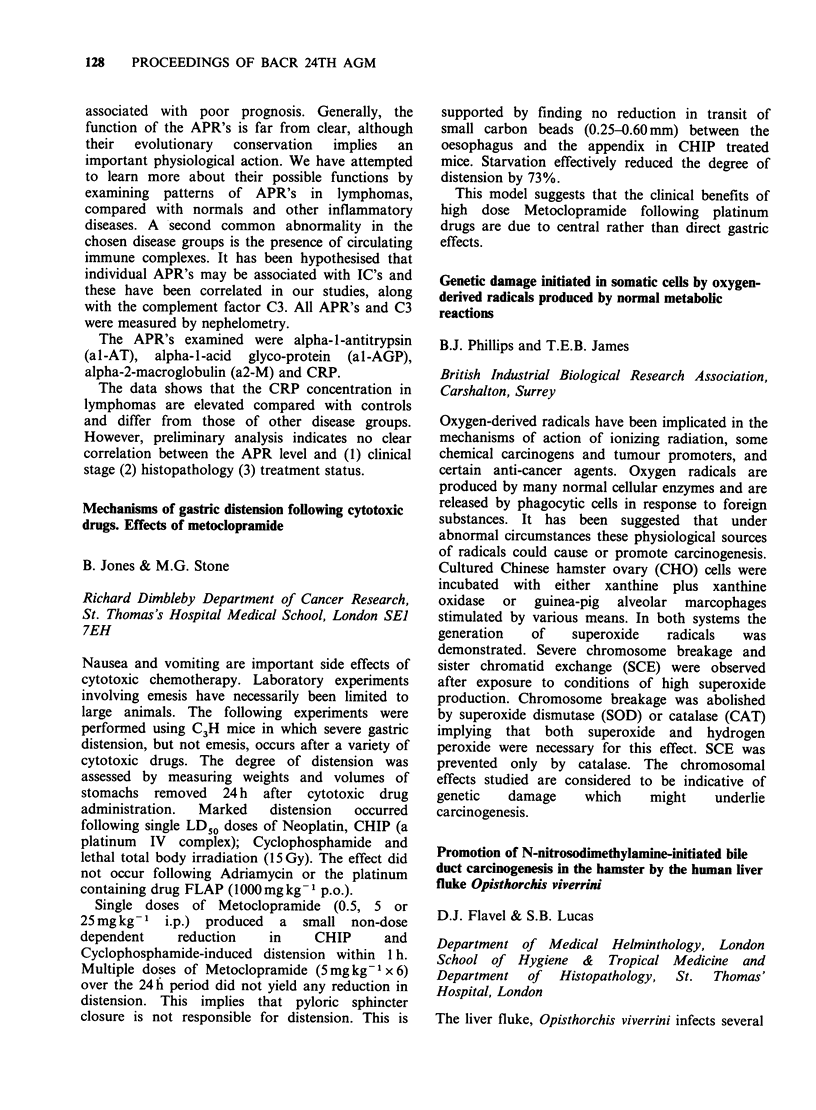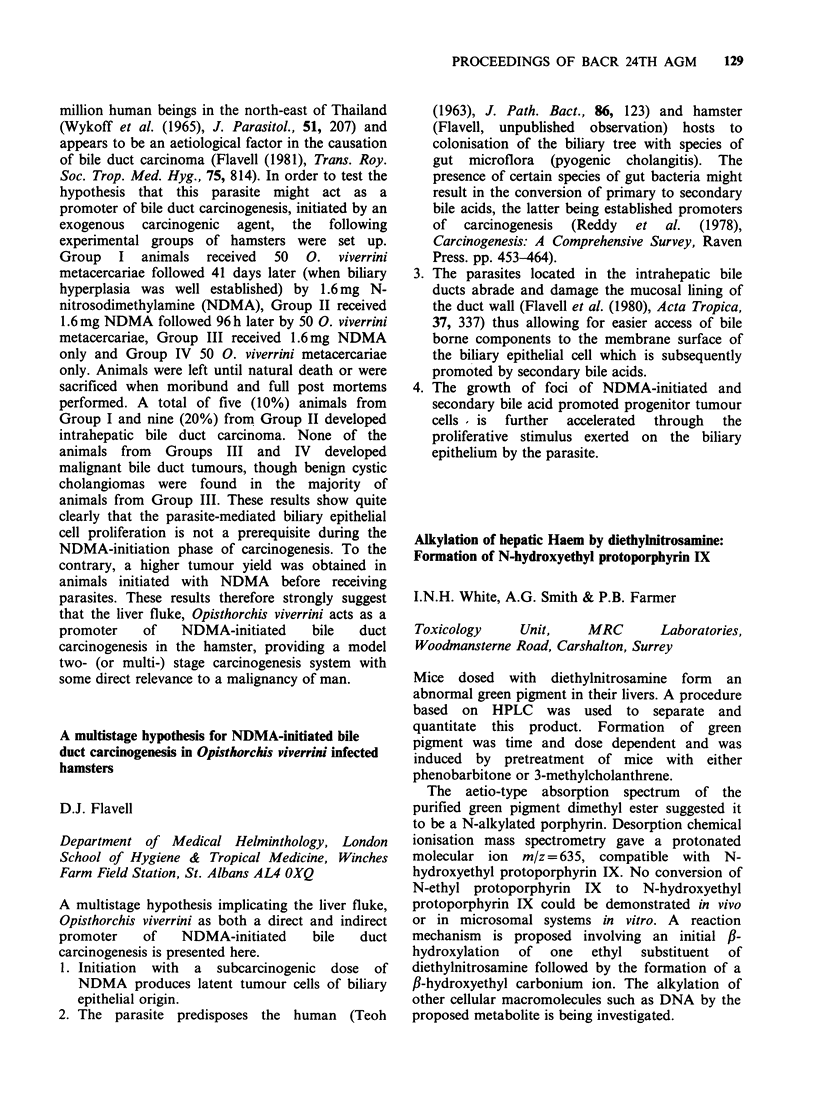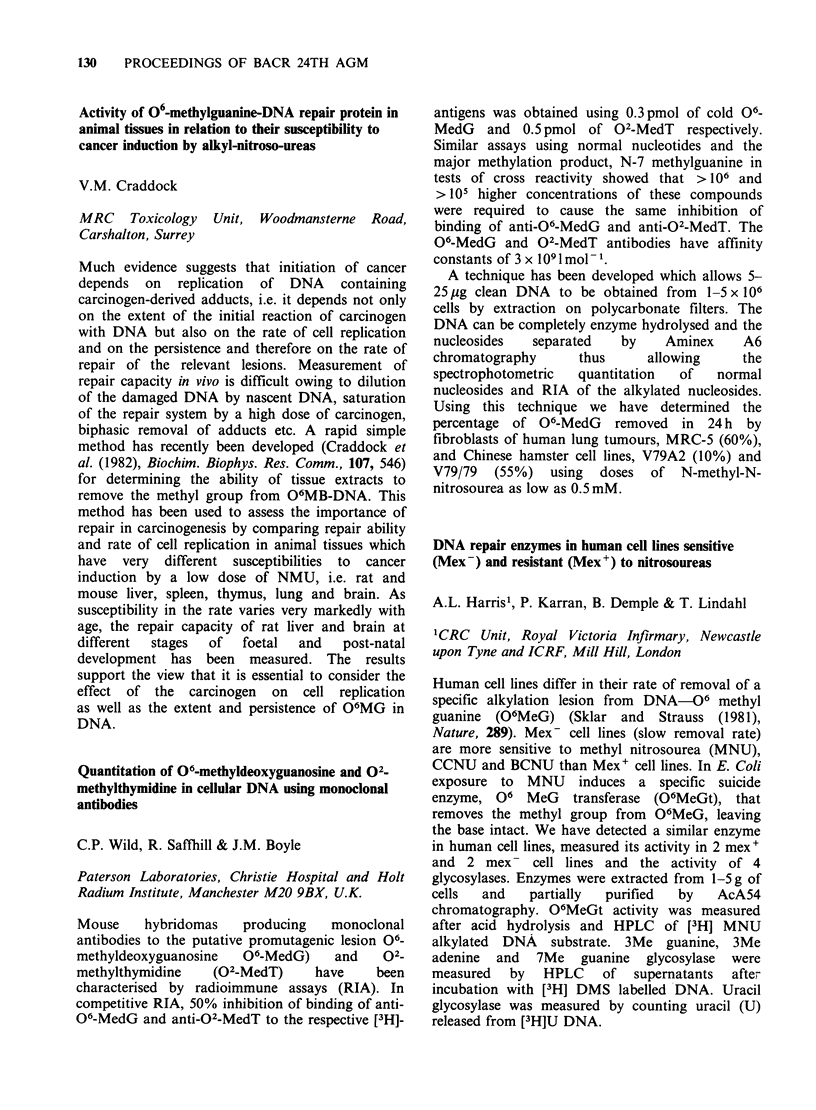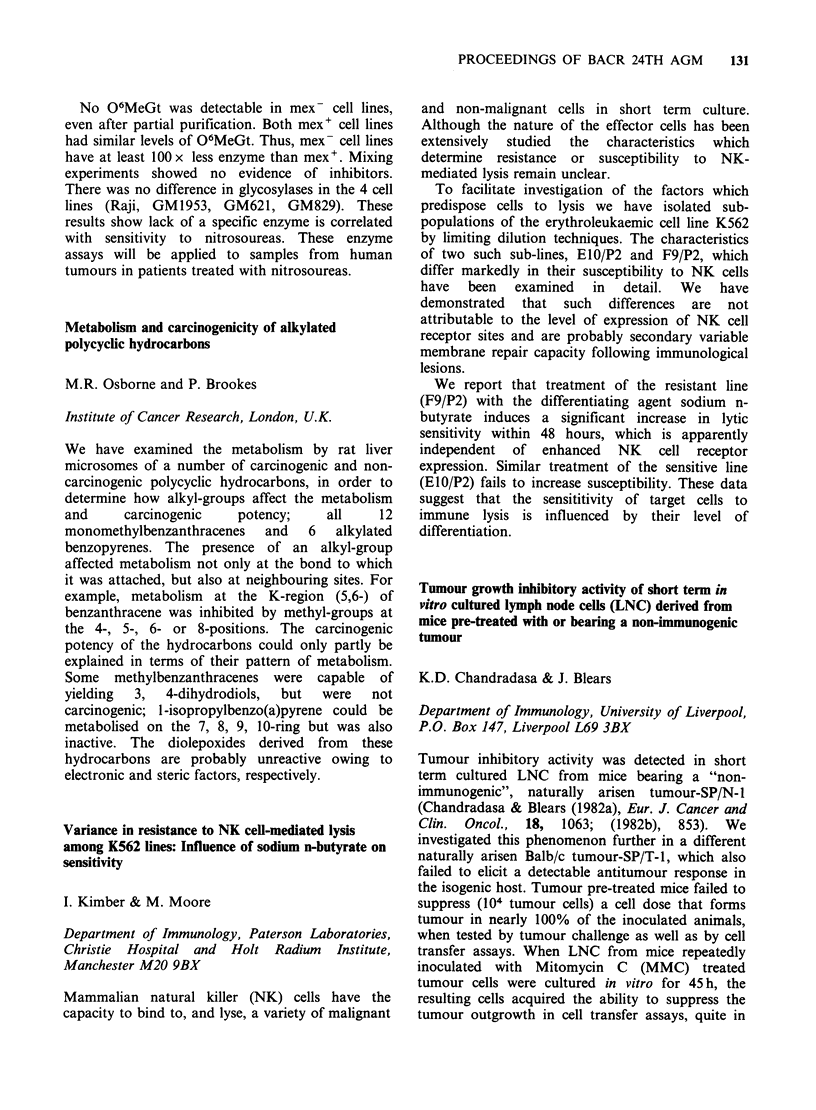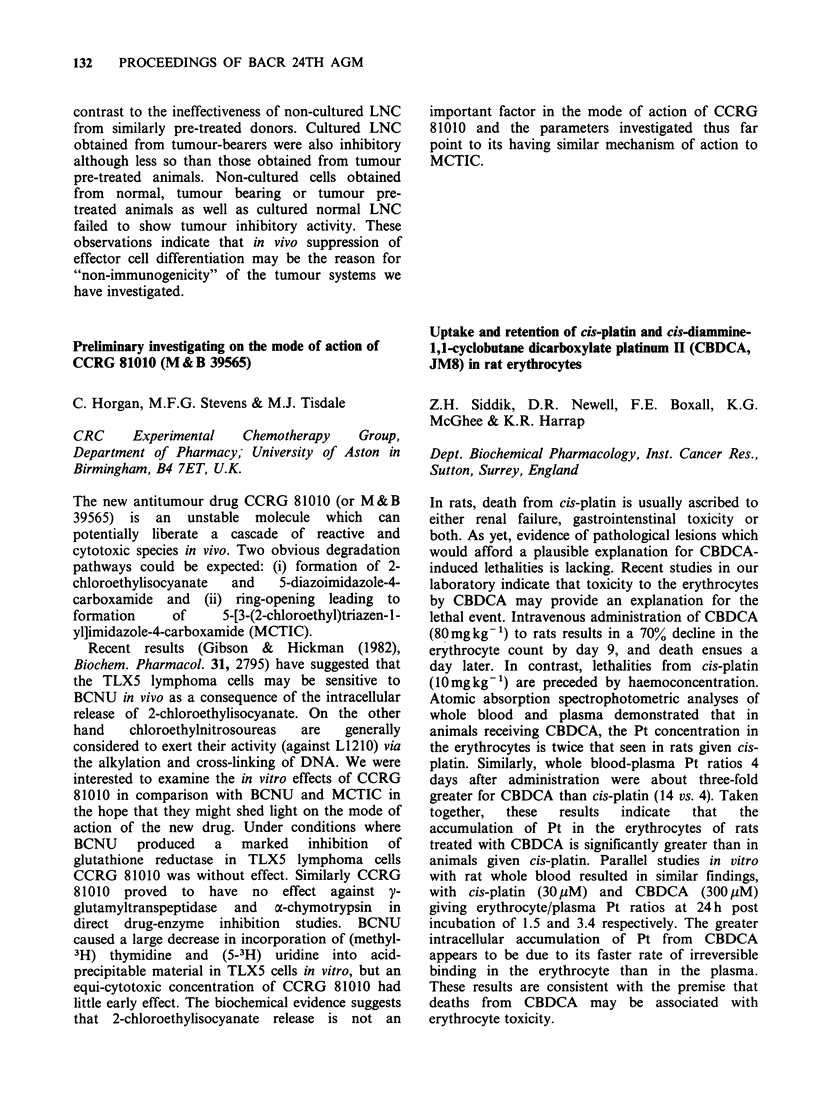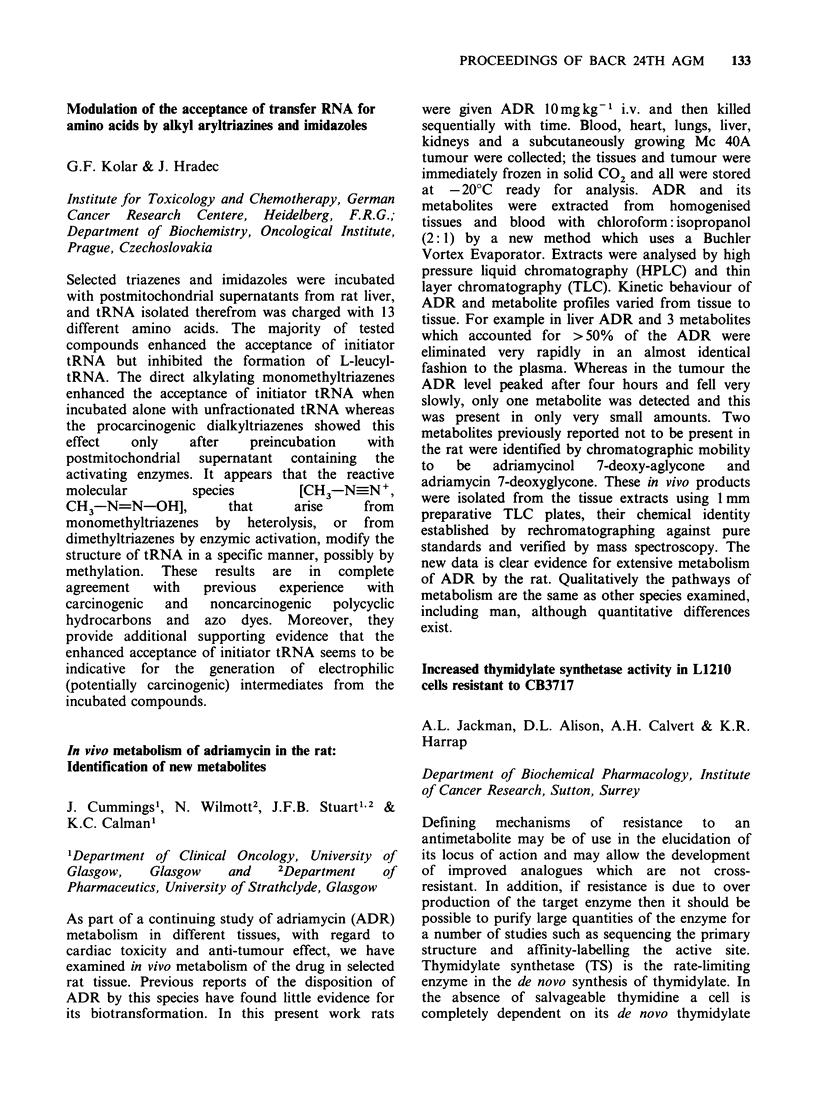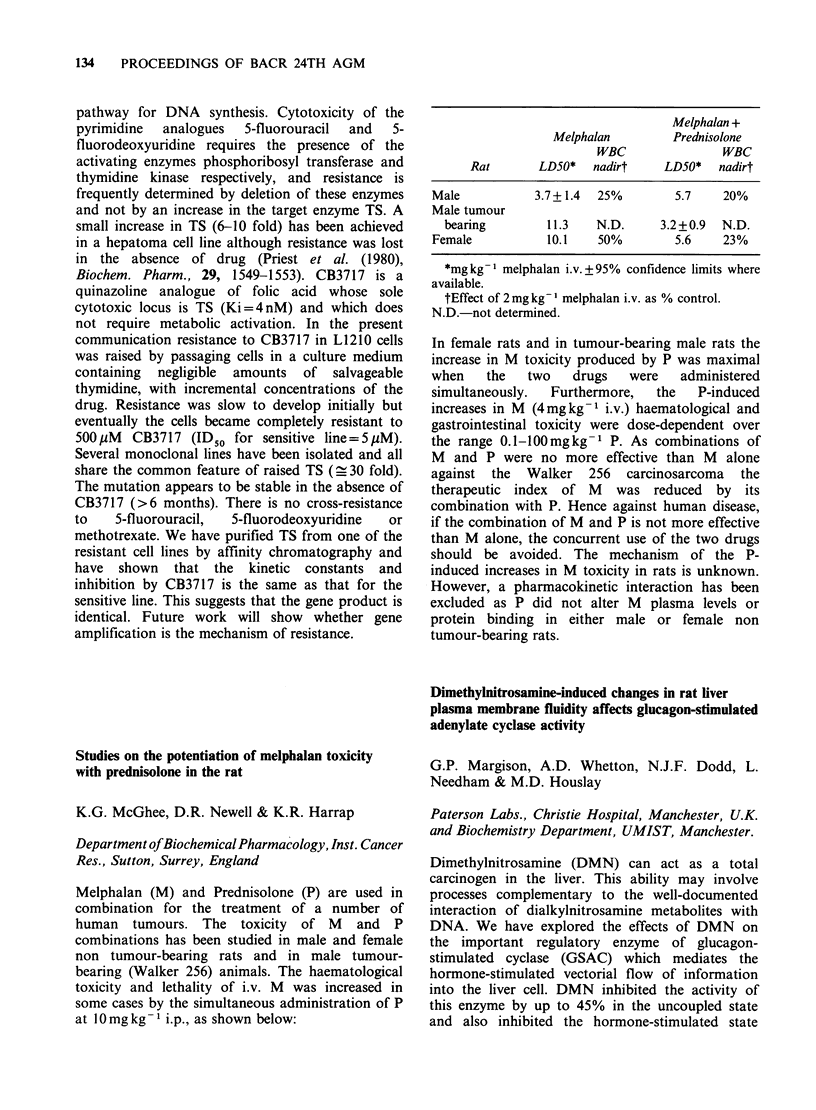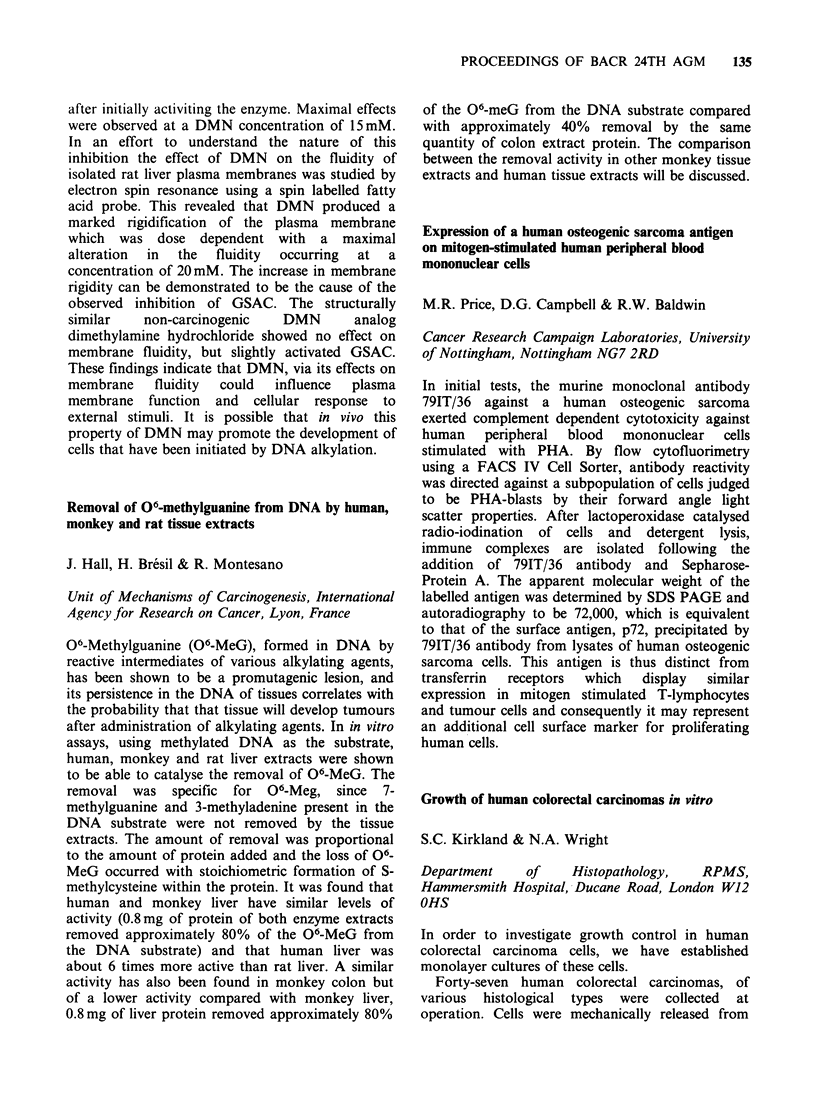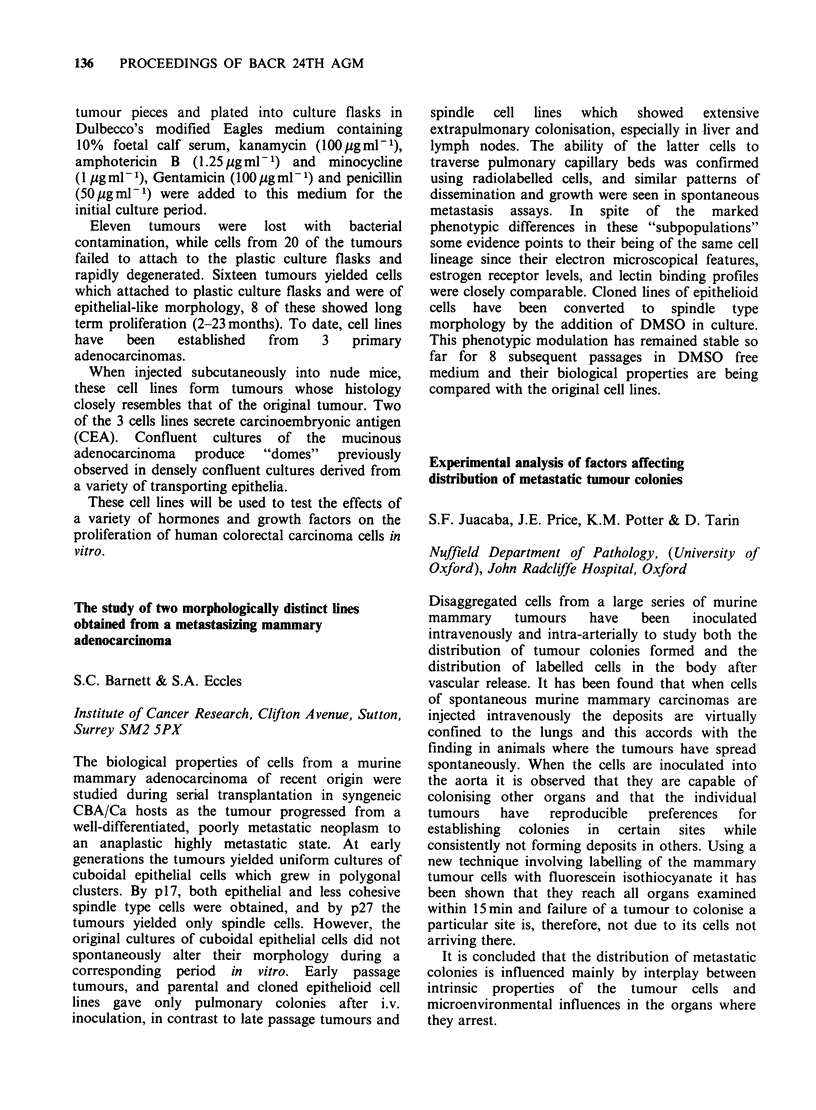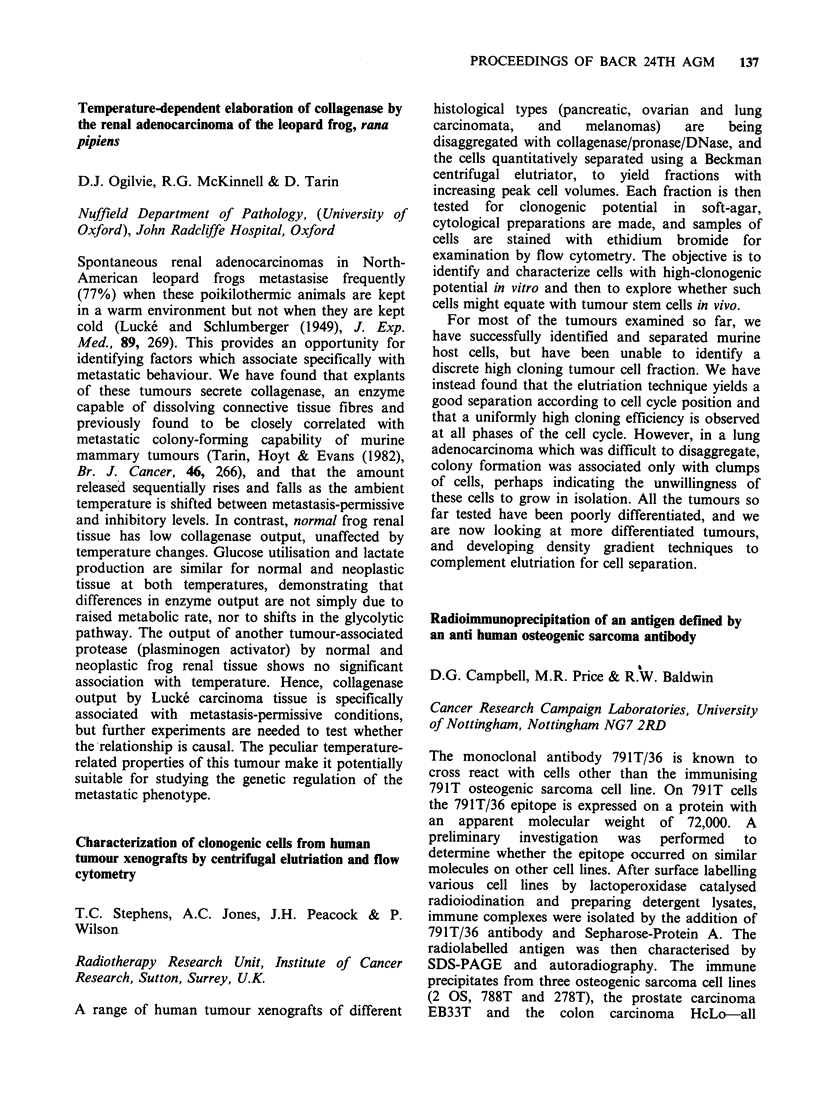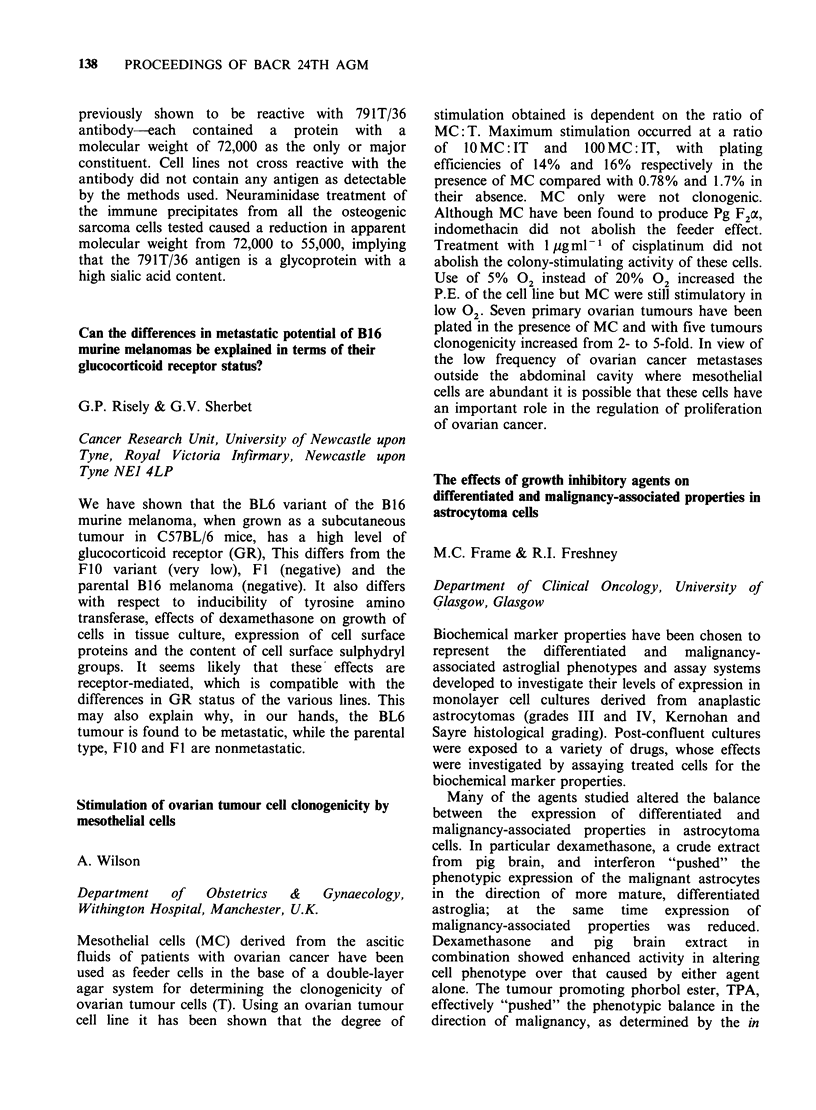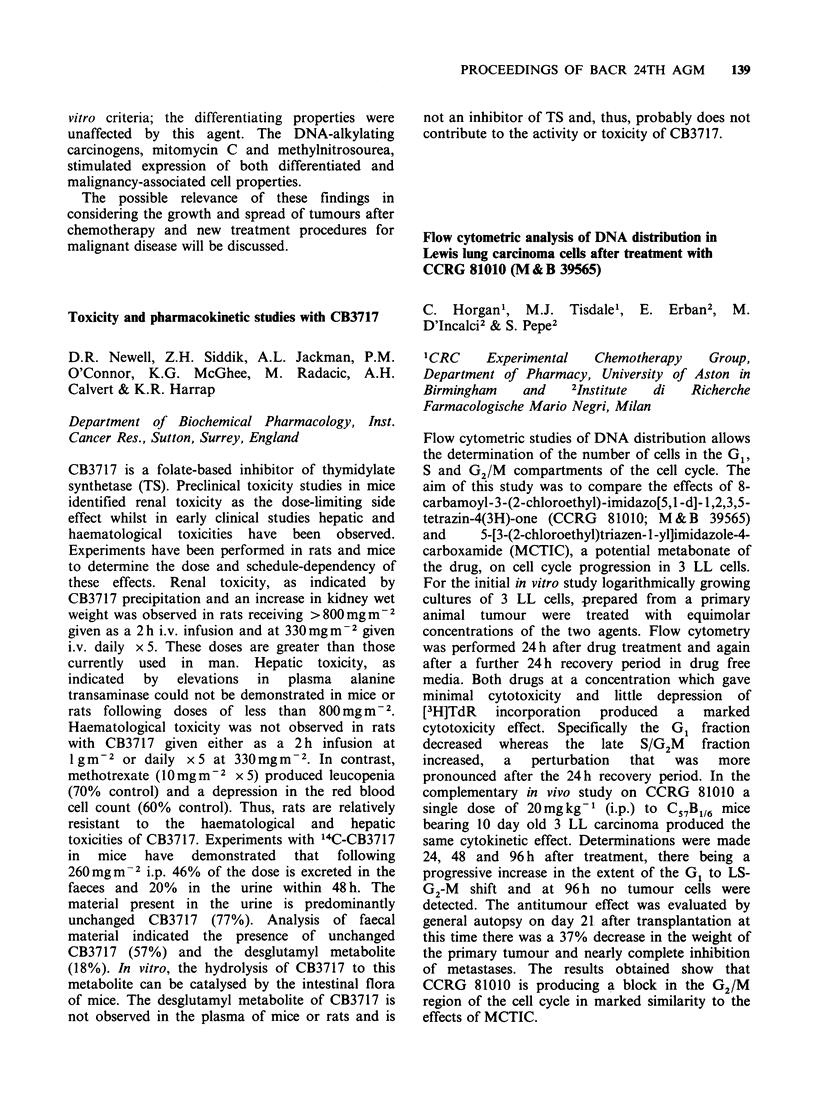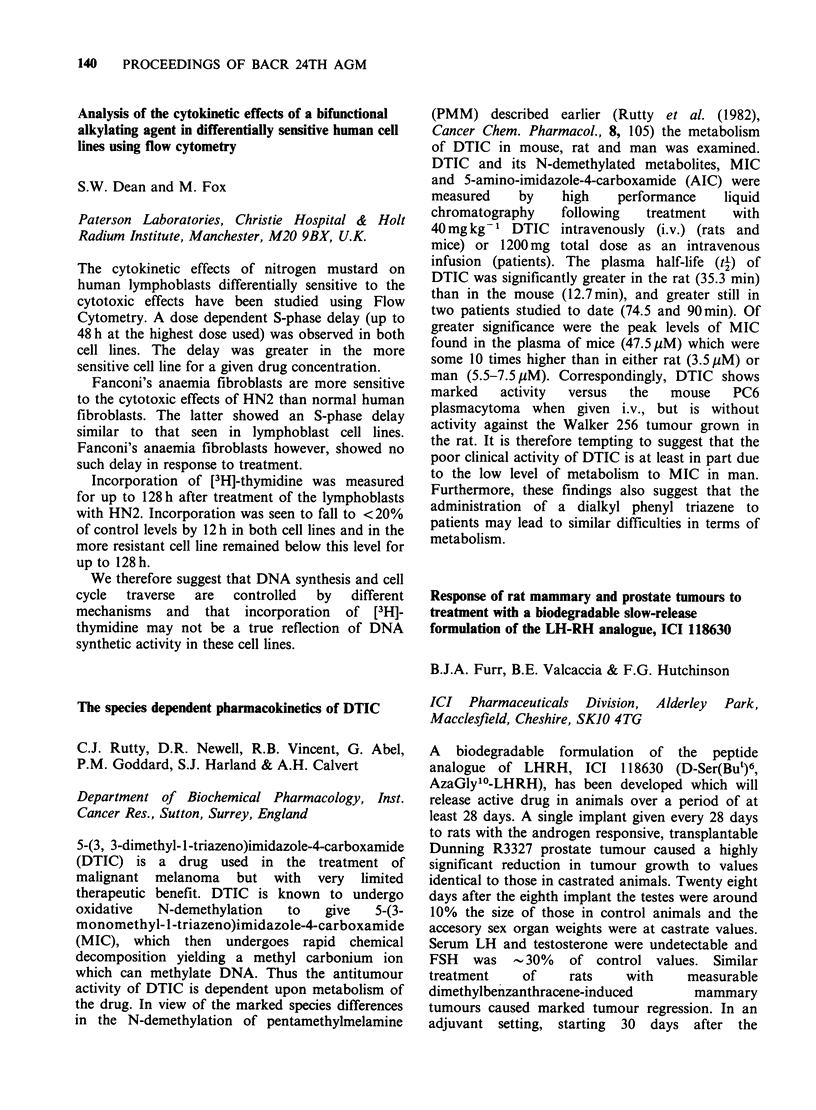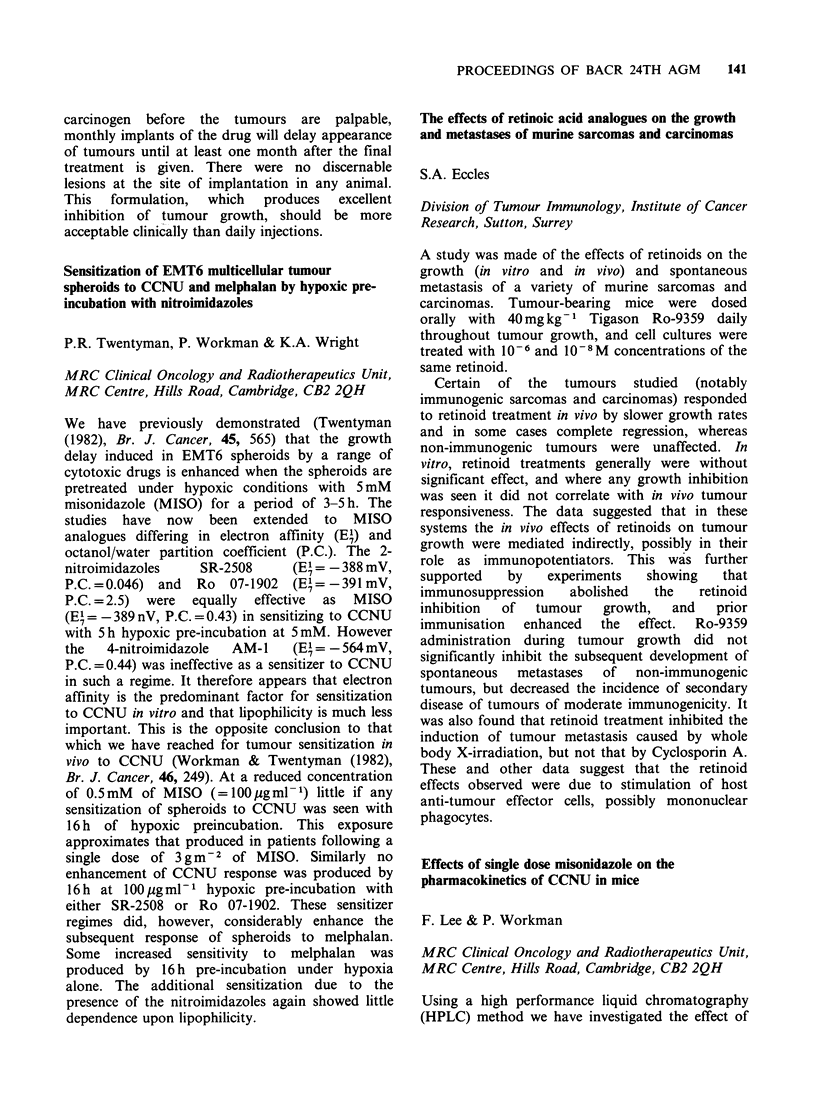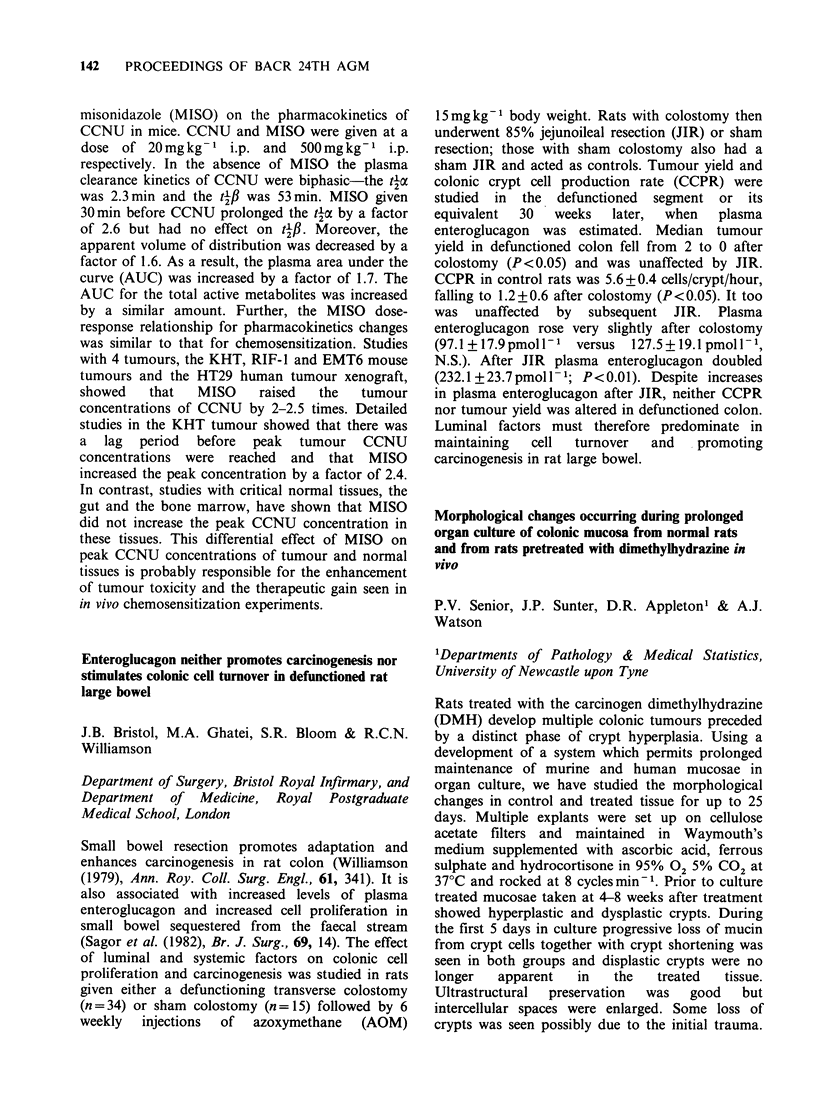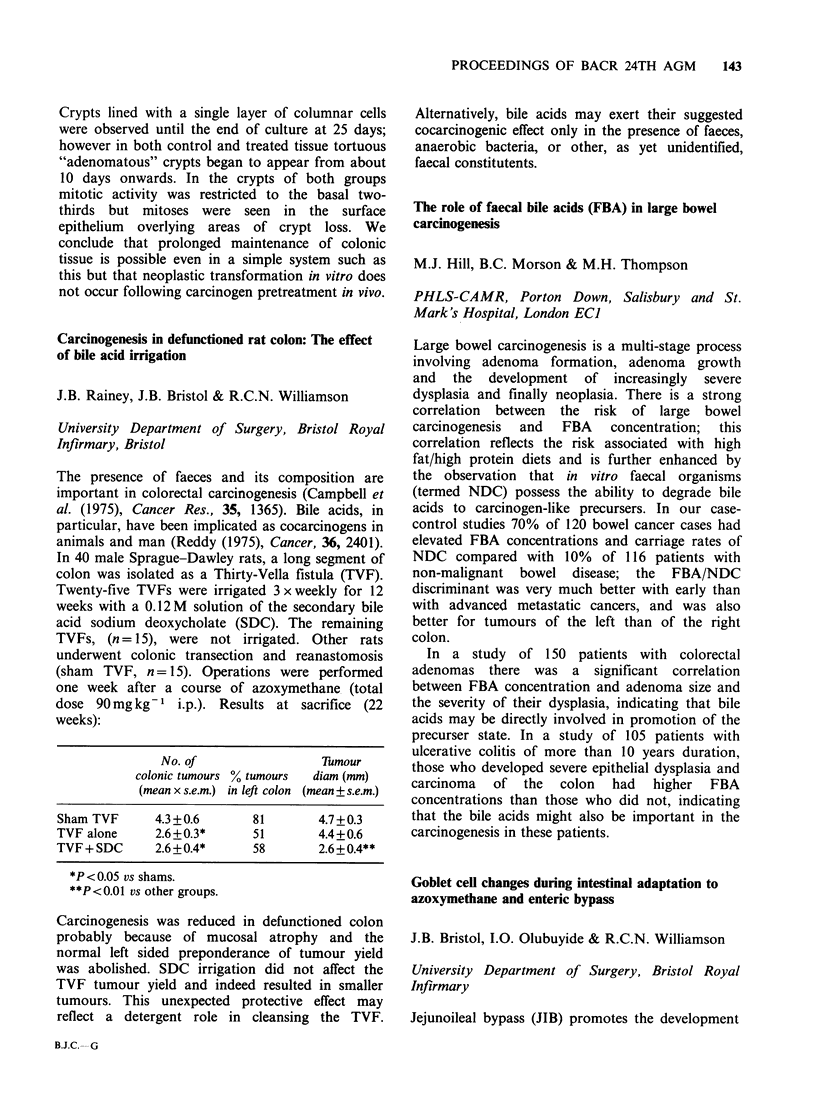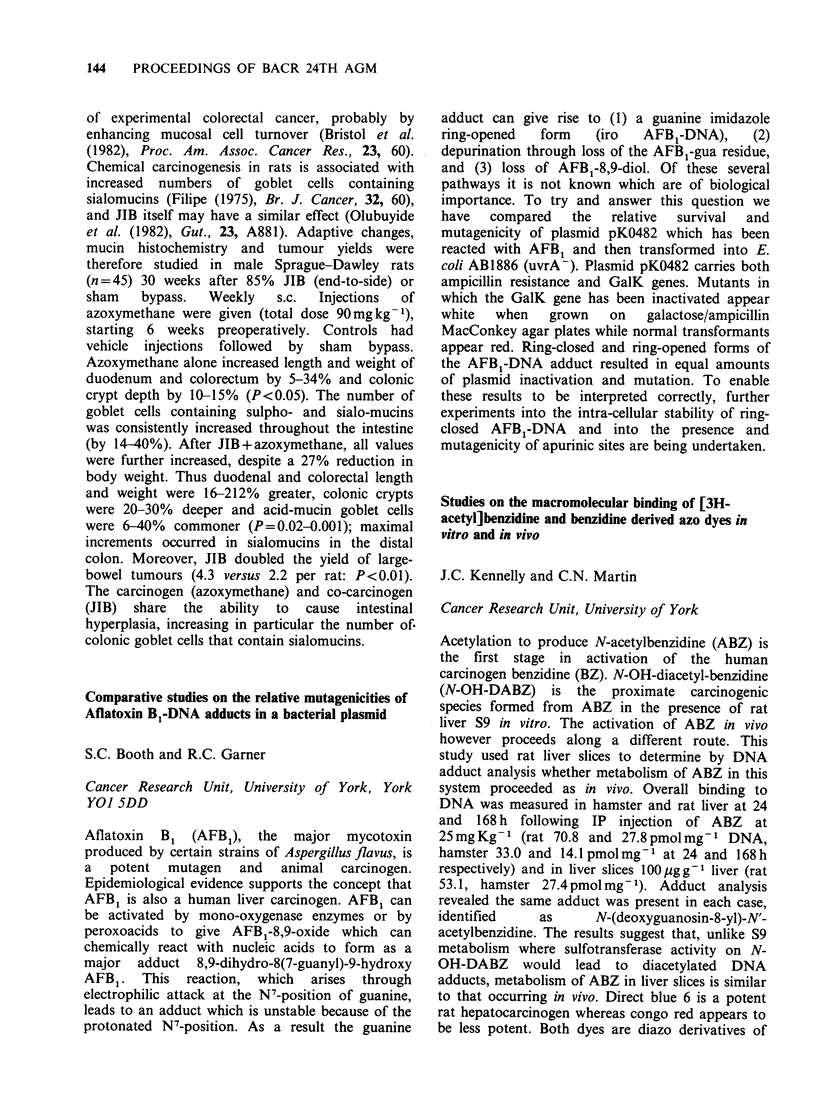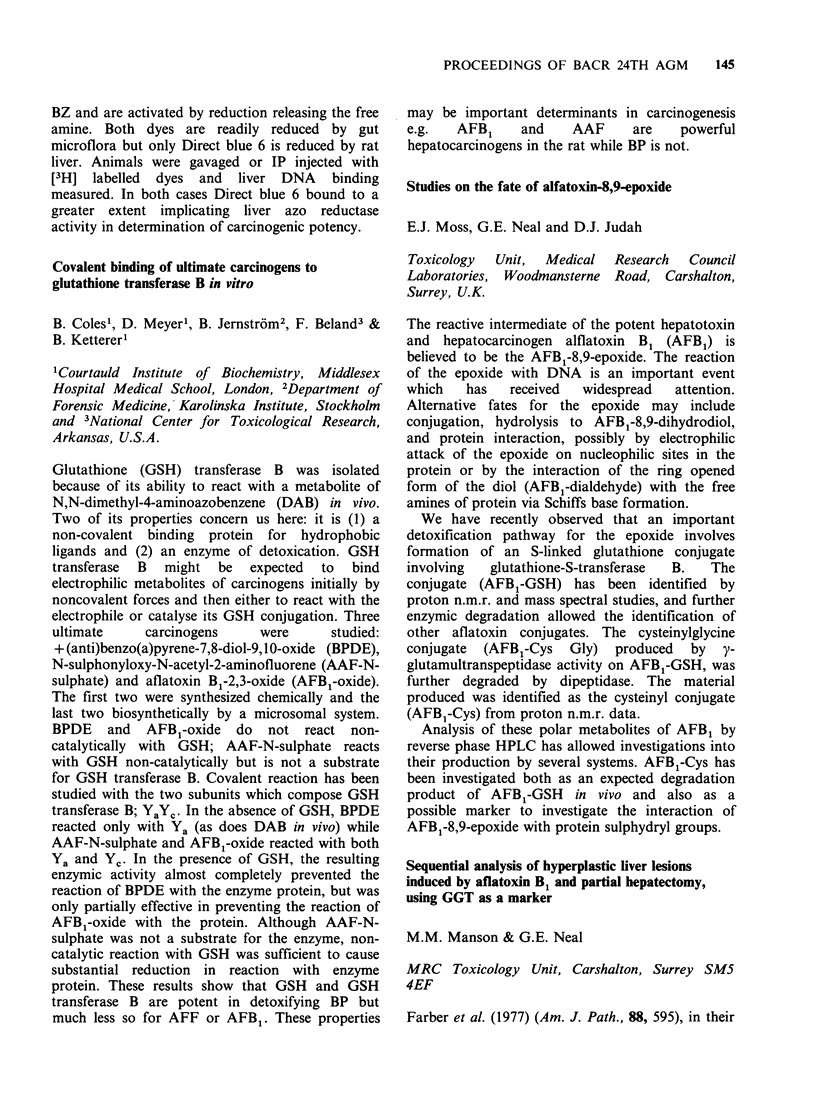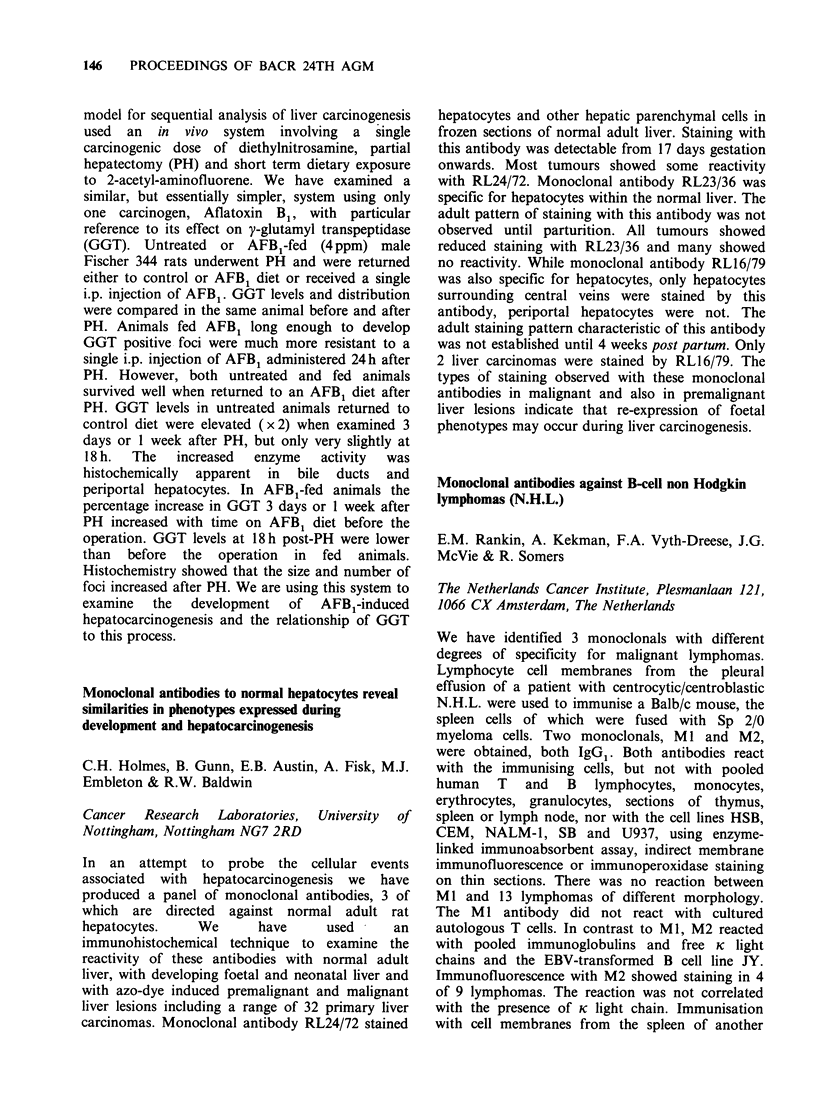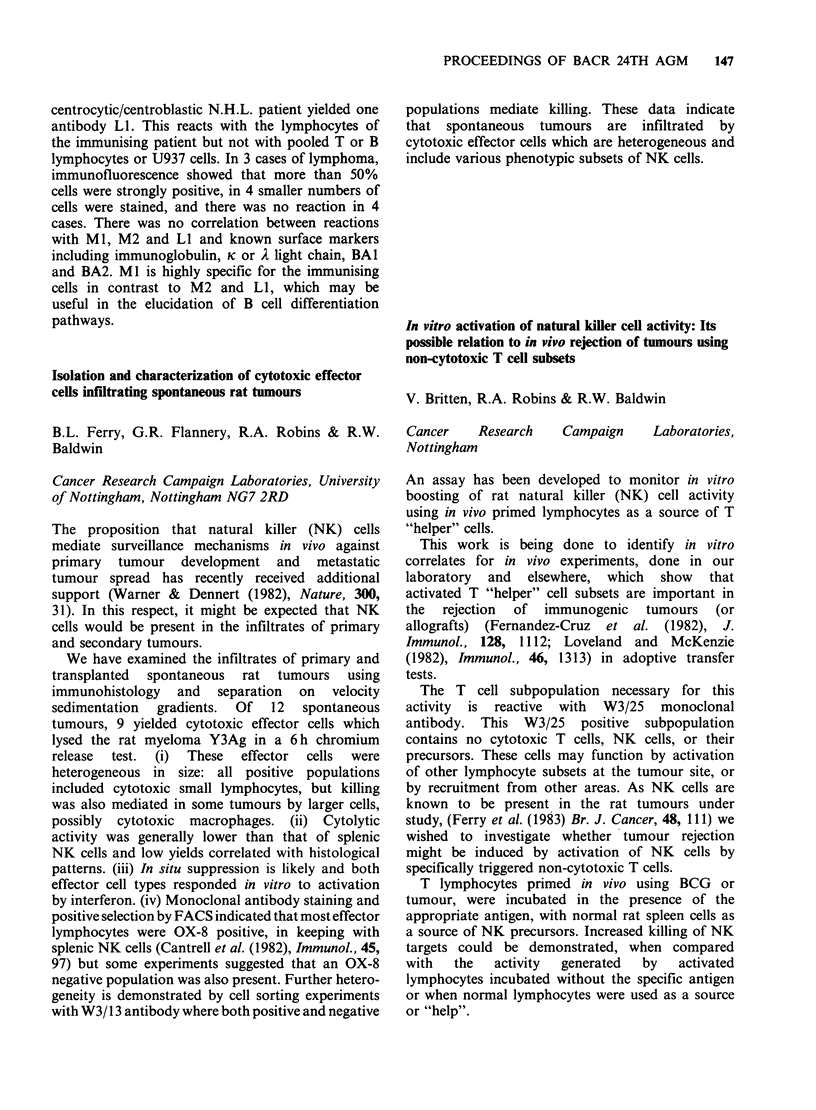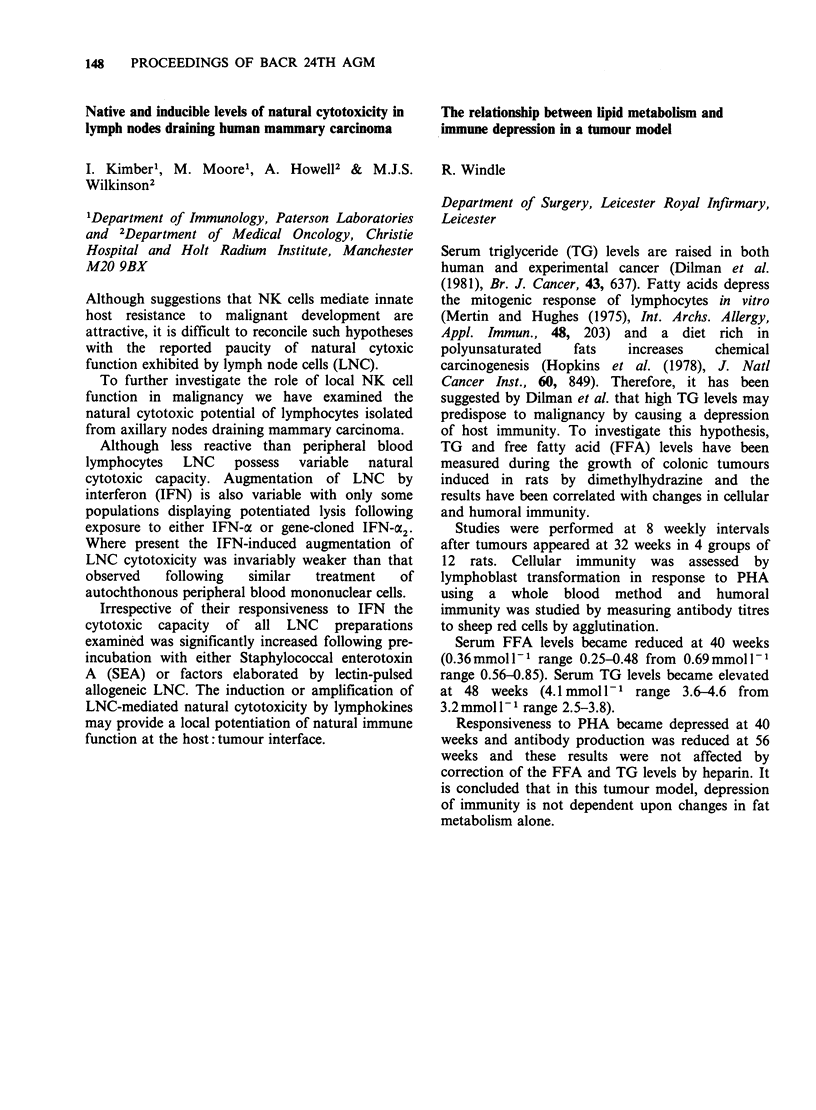# Abstracts for the 24th AGM of the BACR March 23rd-25th, 1983

**Published:** 1983-07

**Authors:** 


Br. J. Cancer (1983), 48, 111-148

### Twenty-fourth Annual General Meeting of the British Association for Cancer Research*

(Incorporating a Symposium on "Environmental Carcinogenesis" and the Walter Hubert Lecture). March 23–25, 1983

*Held at the University of York, Heslington, York*

### Abstracts of Invited and Proffered Papers

### Symposium on "Environmental carcinogenesis"

### Aromatic amines and nitroaromatic hydrocarbons as environmental carcinogens: metabolic activation, carcinogen-DNA adduct formation, and methods for adduct detection

F.F. Kadlubar

*National Center for Toxicological Research, Jefferson, AR, U.S.A. 72079*

Carcinogenic aromatic amines and nitroaromatic hydrocarbons are widely distributed in our environment, being present in cigarette smoke, diesel exhaust, coal oil and shale oil effluents, and polluted air particulates. Metabolic activation may take place in the liver or other target tissues and involves formation of reactive N-hydroxy metabolites or their esterified derivatives. Specific carcinogen-DNA adducts which result from these pathways have now been identified for 2-acetylaminofluorene, 1- and 2-naphthylamine, 4-aminobiphenyl, benzidine, N-methyl-4-aminoazobenzene, 3,2′-dimethyl-4-aminobiphenyl, 4-nitrobiphenyl, and 1-nitropyrene. Recently, we have shown that peroxidation of aromatic amines by the prostaglandin endoperoxide synthetase also results in reactive metabolites that bind to DNA. The identity of these metabolites and their DNA adducts is currently under investigation. Studies conducted thus far indicate that the relative persistence *in vivo* of certain $N^2$- and C8-substituted guanine adducts and their relative mutagenic potencies in bacterial test systems correlate well with tumorigenesis and are consistent with the point mutation (G:T transversion) observed on activation of certain cellular transforming genes. Development of $^{32}P$ reverse-labeling and immunologic methods for detection of these adducts in exposed population may provide a direct assessment of human cancer risk.

### Human monitoring for carcinogen exposure using chemical methods

P.B. Farmer

*MRC Toxicology Unit, Woodmansterne Road, Carshalton, Surrey*

The chemical monitoring of environmental exposure to carcinogens requires the use of exceptionally sensitive analytical techniques. Ideally the reaction product between the ultimate carcinogenic species and the crucial site in DNA should be measured. Normally in practice the nature of this product is not known with certainty and, even if it were, the analysis of such a product would be beset by difficulties caused by its *in vivo* instability and the difficulties in acquiring it in sufficient quantities for chemical analysis. Despite this, postlabelling radiochemical techniques show considerable promise for the sensitive detection of DNA-carcinogen adducts. An alternative approach for routine human monitoring might be the assay of excreted excised DNA-carcinogen adducts, which we are currently studying for some methylating agents.

The reaction products between electrophilic carcinogens and proteins are often more stable and easier to acquire than the DNA-adducts. We have therefore been investigating if the analysis of such products (formed with haemoglobin) is of use for practical monitoring of exposure to alkylating agents. Our initial studies indicate that this is the

*Honorary Secretary, Dr. M.J. Embleton, Cancer Campaign Laboratories, University of Nottingham, Nottingham NG7 2RD, U.K.

case for exposure to some simple alkylating agents such as ethylene oxide and propylene oxide. However interpretation of some results is hindered by the existence of background levels of material. This difficulty has been overcome in experimental systems by the use of stable isotope labelled alkylating agents.

### Detection and quantification of specific carcinogen-DNA adducts in mammalian cells by high-affinity monoclonal antibodies

M.F. Rajewsky

*Institut für Zellbiologie (Tumorforschung), Universität Essen (GH), Hufelandstrasse 55, D-4300 Essen 1, Federal Republic of Germany*

The application of conventional radio-chromatographic techniques to detect and quantitate carcinogen-induced structural modifications in the DNA of mammalian cells, is limited by the necessity to use radioactively labelled agents and by the relatively large amounts of DNA (cells) required for analysis at low levels of modification. Recently developed immunoanalytical methods have improved this situation considerably. Thus, high-affinity monoclonal antibodies, in combination with radio- and enzyme-immunoassays, permit the sensitive detection of alkyl-deoxynucleosides in small samples of hydrolyzed DNA from tissues or cultured cells previously exposed to nonradioactive (e.g., environmental) alkylating N-nitroso carcinogens (see Müller, Adamkiewicz and Rajewsky (1982), *IARC Sci. Publ.* **39**, 443-459). Moreover, a standardised procedure has been established for the use of monoclonal antibodies to quantitate by direct immunofluorescence (and with the aid of a computer-based image analysis of electronically intensified fluorescence signals) specific alkylation products in the nuclear DNA of individual cells (Adamkiewicz, Ahrens and Rajewsky, subm. f. publ.). With this method, the present detection limit for, e.g., $O^6$-ethyldeoxyguanosine ($O^6$-EtdGuo) is of the order of $5 \times 10^2$ $O^6$-EtdGuo molecules per diploid genome. It has, therefore, now become possible to monitor cells (e.g., from biopsy material) directly for the presence of specific carcinogen-DNA adducts, or with respect to their individual capacity for enzymatic removal of such modified structures from their DNA prior to DNA repair. (Supported by the Deutsche Forschungsgemeinschaft, SFB 102/A9, and by the Commission of the European Communities, ENV-544-D[B].)

### The role of viral hepatitis in human liver cancer

A.J. Zuckerman

*London School of Hygiene and Tropical Medicine, London*

The evidence for the implication of hepatitis B virus in the pathogenesis of primary hepatocellular carcinoma is based on epidemiological and geographical observations of a strong correlation between hepatatis B infection and this common and important cancer; a relatively constant risk of developing this type of cancer in both endemic and non-endemic areas among persistent male carriers of hepatitis B surface antigen; infection precedes and may accompany the development of cancer, usually in a liver with chronic damage or macronodular cirrhosis associated with hepatitis B virus; hepatitis B antigens are present in the malignant tissue and there is evidence of covalent integration of the genome of hepatitis B virus into the DNA of the tumour cells; several cell lines derived from primary hepatocellular carcinoma secrete B surface antigen in culture and DNA has been shown to be integrated into the genome of these cells as well as RNA molecules containing specific sequences of hepatitis B virus and at least one of these cell lines has been shown to be heterotransplantable; and the finding of chronic liver damage and primary liver cancer in several animal species infected with viruses which are phylogenetically related to human hepatitis B virus. More recently, the strategy of replication of hepatitis B virus has been shown to have marked similarities to retroviruses, but there are also features which have never been observed with retroviruses.

### Ionising radiation in the environment—sources and effects

A.P. Brown

*National Radiological Protection Board, Chilton, Didcot, Oxon, OX11 0RQ*

The natural and artificial sources of radiation are briefly outlined and their relative contributions to the population dose in the U.K. estimated. The epidemiological evidence relating ionising radiation to cancer induction in humans is reviewed and some of the problems in applying these data to the low levels of radiation in the environment are highlighted. The apparent certainty of cancer risk factors used in radiological protection is contrasted with the uncertainty of the underlying biological events.

### Monitoring human body fluids for carcinogens using bacterial mutation

S. Venitt

*Institute of Cancer Research, Pollards Wood Research Station, Nightingales Lane, Chalfont St. Giles, Bucks. HP8 4SP, U.K.*

The well validated qualitative association between mutagenicity and carcinogenicity has been established largely by extensive use of assays employing reverse-mutation of bacteria from amino-acid auxotrophy to prototrophy (e.g., his−−→his+: trp−−→trp+), using a diverse range of pure chemicals. Because bacterial mutation assays are sensitive, rapid, economical and relatively simple to perform, they are now being used in studies of human body fluids and excreta in order to determine to what extent the presence of mutagens can be implicated in the aetiology of cancers at several sites. The assay of urine for mutagenicity as an indicator of absorption of carcinogens is also gaining wide acceptance. However, the use of reverse mutation assays which were originally designed for testing pure chemicals for determining the mutagenicity of complex biological mixtures is fraught with opportunities for generating artefacts. For example, studies in this laboratory have shown that trace amounts of histidine and tryptophan in faecal extracts can seriously interfere with fluctuation tests, giving false-positive results. The same problem has been encountered in assays of gastric juice, where, in samples from some patients, high levels of free amino acids have been detected. Faeces originate and are stored in the colon, which is anaerobic. However, of several studies of mutagenicity of human faecal extracts now published, none has employed anaerobic mutation assays and the effects of air on the mutagenicity of faecal extracts has not been controlled. We have therefore developed methods for preparing faecal extracts and assaying them for mutagenicity under strictly anaerobic conditions. So far we have found that aqueous faecal extracts prepared and assayed anaerobically gave negative results under conditions where reference mutagens were positive. Extracts of the same stool samples prepared and assayed aerobically were positive, suggesting that air-oxidation may interfere with a true assessment of faecal mutagenicity.

### Abstracts of members' proferred papers

### Pharmacokinetic and preliminary clinical toxicity data on TGU

J.F.B. Stuart[1,2], J. Welsh[1], A. Setanoians[1], S. B. Kaye[1] & K.C. Calman[1]

[1]*Department of Clinical Oncology, University of Glasgow, Glasgow and* [2]*Department of Pharmaceutics, University of Strathclyde, Glasgow*

TGU is a new and comparatively stable epoxide which is thought to act as an alkylating agent. It has anti-tumour activity in a wide range of experimental tumours and is active in cyclophosphamide resistant P388. An HPLC analytical method has been developed with a sensitivity of 10 mg ml$^{-1}$. Pharmacokinetic data have been obtained from male Porton mice. The data fit a two compartment pharmacokinetic model with a bi-exponential decay. The $t_{\frac{1}{2}}\alpha$ is 1.5 min $t_{\frac{1}{2}}\beta$ 5 min, area under the curve (AUC) 57.74 $\mu$g ml$^{-1}$ min$^{-1}$, elimination constant 0.1386 and $V_D$ 0.7497 ml. The patients study includes doses up to 120 mg m$^{-2}$ and 10 patients have received the drug. The pharmacokinetics in 5 patients show a short $t_{\frac{1}{2}}\beta$ and no toxicity has been experienced at this dose of the drug.

### Effects of misonidazole (MISO) on the pharmacokinetics of 5-fluorouracil (FU) in humans

B.J. McDermott, H.W. van den Berg,[1] W.M.C. Martin[2] and R.F. Murphy

*Departments of Biochemistry,* [1]*Therapeutics and Pharmacology, Queen's University of Belfast and* [2]*Belvoir Park Hospital, Belfast*

Five patients with gastrointestinal cancer received FU (1.0 g m$^{-2}$), MISO (1.75 or 2.0 g m$^{-2}$) and FU (1.0 g m$^{-2}$), FU (1.5 g m$^{-2}$), MISO (1.75 or 2.0 g m$^{-2}$) and FU (1.5 g m$^{-2}$), consecutively, at monthly intervals. Elimination of FU and metabolites in urine during 48 h after drug administration were analyzed using a F$^{-}$-specific electrode after combustion of specimens in an oxygen flask. MISO had a potentiating effect on the retention of total drug in 9 of the 10 courses of treatment investigated. The areas under the "Sigma-minus" curves (AUC) of drug elimination were enhanced by 2–33% of those observed when FU was given alone. Delayed clearance of FU from the body in the presence of MISO was reflected in a reduced extent of urinary excretion of total drug during 6 courses of therapy. The anomalous trends in excretion parameters may be explained by the clinical conditions. Patient toxicity during 5 of the treatments corresponded with the highest AUC values. These findings are a preliminary indication that MISO may have an effect on drug pharmacokinetics in humans. Furthermore, the mechanism of MISO potentiation of FU action cannot be a competition for microsomal enzymes, as proposed for the interaction of MISO and alkylating agents, since FU is catabolized at mitochondrial and cytosolic sites.

### Pharmacokinetics of etoposide (VP) in patients with small cell lung cancer

J. Edmundson, N. Thatcher, P.M. Wilkinson, J. Margison, W. Steward, W. Chiu & J. Wagstaffe

*Departments of Medical Oncology and Clinical Pharmacology, Christie Hospital, Manchester M20 9BX*

VP alone or in combination is now used extensively for the management of small cell lung cancer (SCLC), but at present there is no definitive dose schedule. We therefore determined the kinetics in 17 patients with (SCLC). VP was administered in 3 doses of 125, 300 and 600 mg m$^{-2}$ in 500 ml N.saline over 30 min.

Patients given the first dose also received cyclophosphamide (C) 2.5 g m$^{-2}$ i.v. Results are summarised in the Table.

| *Dose mg m$^{-2}$* | *125* | *300* | *600* |
|---|---|---|---|
| Parameter (mean ± s.e.) | | | |
| $T_{\frac{1}{2}}\alpha$ (min) | 22±5.4 | 12±5 | 20±2.4 |
| $T_{\frac{1}{2}}\beta$ (h) | 5.3±1 | 4.5±95 | 6.1±1.5 |
| AUC ($\mu$g l$^{-1}$ h$^{-1}$) | 84±9.8 | 255±23 | 591±83 |
| CL (ml min$^{-1}$) | 48±8.5 | 33.23±3.3 | 32.6±5.1 |

In all instances an open 2 compartment model would fit the observed serum concentrations. The incremental dose increase produced the expected linear increase in AUC but not drug distribution or clearance. There was no evidence to suggest that the rate of biotransformation of C was altered by VP. The results suggest that a single bolus dose may be as effective as fractionated bolus schedules.

### Pharmacokinetics of *cis*-diammine-1,1-cyclobutane dicarboxylate platinum II (CBDCA, JM8) in patients with normal and abnormal renal function

S.J. Harland, D.R. Newell, Z.H. Siddik, R. Chadwick, A.H. Calvert & K.R. Harrap

*Inst. Cancer Res. and Royal Marsden Hosp., Sutton, Surrey, England*

CBDCA is an analogue of cisplatin which is free of the nephrotoxicity (Calvert *et al.*, (1982), *Cancer Chemother. Pharmacol.* **9**, 3) but appears to retain the efficacy. Its limiting toxicity is haematological and patients with poor renal function are particularly susceptible to this.

The pharmacokinetics following a 1 h infusion were studied in patients receiving doses between 20 and 520 mg m$^{-2}$. Renal function was assessed in every case by $^{51}$Cr EDTA clearance. Total plasma platinum (Pt) and free Pt (in plasma ultralfiltrate) were measured by atomic absorption spectrophotometry. Intact CBDCA was measured by HPLC. There was a linear relationship between dose and area under the plasma concentration curve (AUC) for total Pt. Protein binding during the first 4 h was between 0 and 28%. It rose to 85–95% by 24 h. All the free Pt was in the form of CBDCA during the first 4 h. An early elimination phase for free Pt had a half-life of 91±6 (s.e.) mins, similar to that for CBDCA and total Pt. Later half-lives of 279±24 min and >24 h were seen for free and total Pt respectively. 65±1% of the administered Pt appeared in the urine over the first 24 h. Patients with poor renal function have higher AUCs for total Pt. Both renal and total clearance of free Pt correlated significantly with glomerular filtration rate. These findings justify the practice of reducing the dose of CBDCA in the presence of renal impairment.

Renal clearance of free Pt following CBDCA was 0.67±0.05 $^{51}$Cr EDTA clearance, suggesting that there was no tubular secretion of the drug as occurs with cisplatin (Jacobs *et al.*, (1980), *Cancer Treat. Rep.* **64**, 1223). This may account for the difference in nephrotoxicity.

**Plasma levels of N-methylformamide following intravenous and oral administration in man**

C. Brindley, P. Kestell, A. Gescher and J.A. Slack

*CRC Experimental Chemotherapy Research Group, University of Aston, Birmingham*

N-Methylformamide (NMF, NSC 3051) is a stable, water soluble liquid with a remarkably wide spectrum of antitumour activity in mice (Gescher *et al.* (1982), *Br. J. Cancer*, **45**, 843). One of the most attractive properties of NMF which led to the renewed interest in its clinical applicability is the complete absence of deleterious effects on the bone marrow in experimental animals (e.g. in mice: Langdon *et al.*, unpublished). However, in a clinical trial in 1956 NMF caused symptoms of hepatotoxicity in the seven patients treated (Myers *et al.* (1956), *Cancer*, **9**, 949). As part of Phase 1 studies carried out at the Dutch Cancer Institute, Amsterdam (G. McVie) and Charing Cross Hospital (E. Newlands) concentrations of NMF in the plasma of patients were measured by gas-liquid chromatography. Six patients with a variety of malignancies received either 300, 600 or 1200 mg m$^{-2}$ NMF by both i.v. infusion and p.o. administration with fruit juice. Peak plasma levels of NMF after infusions of 600 mg m$^{-2}$ NMF in two patients were 13.1 and 20.5 $\mu$g ml$^{-1}$, and peak plasma levels after oral ingestion of 600 mg m$^{-2}$ NMF were 15.0 and 13.4 $\mu$g ml$^{-1}$. Twelve h after administration NMF plasma levels had declined to 37.6±23.5% of peak concentration in all patients. When the areas under the plasma concentration of NMF vs time curves (calculated from the time of administration to either 12 h or 24 h after administration) after i.v. and p.o. administration were compared, the bioavailability values (190%, 290%, 170%, 60%, 70% and 180%) were found to be very variable. It appears however that in further clinical trials to evaluate the antitumour efficacy of NMF the drug can be given orally.

**Platinum-induced emesis—the effect of escalating doses of metoclopramide (M)**

S.G. Allan, M.A. Cornbleet, P.S. Warrington, S.P. Lockhart, R.C.F. Leonard & J.F. Smyth

*Department of Clinical Oncology, Western General Hospital, Edinburgh*

It has recently been claimed that high doses of M convey significant protection against platinum-induced emesis. The aims of this study are to examine: (1) the differences in anti-emetic effect of escalating doses of M, and (2) the difference in side effects of M at these dose levels. In a double blind, randomised, prospective study, M was given by 2 hourly 100 ml infusions × 5 starting ½ hour prior to *cis*-platinum infusion. 35 patients have been entered to-date. The mean age was 52 (18–70) and patients received *cis*-platinum alone or in combination for various tumours (ovary, bronchus, teratoma, bladder and melanoma). 17 patients have completed treatment at each of three dose levels of M, 3 mg kg$^{-1}$ (low dose, LD), 5 mg kg$^{-1}$ (moderate dose, MD) and 10 mg kg$^{-1}$ (high dose, HD). A total of 61 courses have been evaluated, 19 at LD, 21 at MD and 21 at HD. M was tolerated well at all dose levels. 1 patient at HD and 1 at MD had easily reversible extra-pyramidal reactions and 11 patients (6 on LD) had mild drowsiness. Nausea was abolished in 5% of patients on LD, 19% on MD and 19% on HD. Vomiting was abolished in 5% of patients on LD, 19% on MD and 14% on HD. Diarrhoea was not increased at the higher doses. To-date it would appear that the side effects of M are not increased at the higher doses. This double blind trial continues and data will be presented on at least 35 patients (>100 treatment courses) together with a statistical analysis of the apparent improved effect of doses of M in excess of 3 mg kg$^{-1}$.

**An evaluation of prednisolone as an appetite stimulant in cancer patients**

J.C. Willox[1], J. Corr[2] & K.C. Calman[1]

[1]*Department of Clinical Oncology, Gartnavel General Hospital, Glasgow and* [2]*Department of Dietetics, Gartnavel General Hospital, Glasgow*

Cancer patients, at all stages of their disease complain frequently of anorexia. Prednisolone has been used empirically, for many years to improve well-being and stimulate appetite in cancer patients with advanced disease. This study examined the effect of prednisolone against placebo on the appetite, body weight, food intake and general well-being of 41 oncology out-patients. The patients had a variety of solid tumours; ages ranged from 27 to 80 y (mean 60 y) with male:female ratio 16:25. Eighteen of the patients received regular chemotherapy which coincided with the study timings. The study was double-blind and each patient received an initial two week course of tablets—prednisolone (5 mg) or placebo—1 tablet t.i.d. and then the alternative tablets for a further two weeks. Assessment was by completion of visual analogue scales and answers to questions on appetite, well-being and nausea. A 24-hour dietary recall history was taken and analysed for calorie and protein content. Anthropometric measurements of weight and skinfold thickness were taken. Results show that 82% patients reported improved appetite when commenced initially on prednisolone. This' compares with 50% patients who found benefit from initial placebo tablets. Of the 82% with improvement on prednisolone, only 60% maintained this when crossed over to placebo. The 50% placebo responders increased to 78% when crossed over to prednisolone. This appetite improvement was reflected in an improved food intake on prednisolone initially (69%) compared to placebo (56%). Weight gain, however, showed little difference between groups. In conclusion, the results indicate an improvement in appetite and food intake for a majority of patients when taking prednisolone. Further studies are required to evaluate long term use of prednisolone.

### Pilot study of multiple modality therapy for advanced ovarian adenocarcinoma

G.J.S. Rustin, B. Southcott, M. Glaser, D. Parker, E.S. Newlands, R.H.J. Begent & K.D. Bagshawe

*Departments of Medical Oncology and Radiotherapy, Charing Cross Hospital, London W6 8RF*

Despite dramatic reductions in tumour bulk by surgery and chemotherapy the percentage of patients with advanced ovarian adenocarcinoma who become long term survivors is small. Radiotherapy has been shown to improve survival of patients with minimal residual disease following surgery (Dembo *et al.* (1979), *Am. J. Obstet. Gynecol.*, **134**, 793). A pilot study on 15 women has therefore been performed to determine the feasibility of giving multiple modality treatment for Stage III and IV ovarian adenocarcinoma. Successful initial debulking surgery has been performed in 13 patients. Eleven patients have so far completed 4 courses of *cis*-platinum $100\,mg\,m^{-2}$ alternating with 4 courses of cyclophosphamide and methotrexate over a mean of 13 weeks. At second look surgery 7 patients were macroscopically in complete remission, 3 were partial responses and 1 had stabilisation of disease. The 9 patients with $<1$ cm maximum diameter residual disease then received a mean of 2507 cGy whole abdominal irradiation in 20–25 fractions over 23–38 days. No treatment was then given until relapse. The actuarial median survival has not been reached by 95 weeks. Two of the 9 patients who completed all treatment have died, 2 are alive with disease and 5 are disease free. No long term toxicity has been observed. As this study is of comparable toxicity to other currently used regimens, but of shorter duration, a randomised trial is now indicated to determine whether this approach can increase the percentage of long term survivors.

### Phase I studies with CB3717 (N-(4-(N-amino-4-hydroxy-6-quinazolinly)Methyl)prop-2-ynylamino)benzoyl)-L-glutamic acid)

A.H. Calvert, D.L. Alison, S.J. Harland, A.L. Jackman, C.J. Mooney, I.E. Smith & K.R. Harrap

*Department of Biochemical Pharmacology, Institute of Cancer Research, Sutton and The Royal Marsden Hospital*

CB3717 has been used to treat 50 patients with advanced malignant disease. Initially doses were infused over 1 h. The toxicities observed using this schedule were rises in the plasma transaminase levels, generalised malaise, occasional skin rashes and myleosuppression. The most consistently observed toxicity was the rise in the plasma transaminase levels which occurred in approximately half the patients. Animal studies suggested that this could be due to high biliary concentrations of CB3717 giving rise to intracanalicular precipitation of the drug. The use of a 12 h infusion of CB3717 was therefore also investigated in patients. Doses of CB3717 were escalated from $140–550\,mg\,m^{-2}$. Dose limiting toxicity has not yet been reached. Rises in plasma transaminase levels occurred following treatment with either protocol with approximately equal frequency. No dose response relationship has yet been observed between the dose given and the magnitude or the frequency of the rise in plasma transaminases. Rises in plasma phenylalanine levels were also noted and could be due to inhibition of biopterin dependant phenylalanine hydroxylation. Skin rashses were observed which were erythematous or maculopapular and itchy and were usually confined to the trunk or legs. Other antifolates (eg metoprin) may cause rashes due to elevated histamine levels, due to inhibition of histamine degradation. Studies of CB3717 and related quinazolines showed that although compounds in the 2,4-diamino series were inhibitors of these enzymes, those in the 2-amino-4-hydroxy series (including CB3717) were not. Plasma histamine levels were not elevated. When myleosuppression occurred the white count nadir was 12 days after treatment, and recovery occurred over a few days. Four partial responses were seen, 2 in patients with breast cancer, both heavily pre-treated, one in carcinoma of the ovary, and one in large cell adenocarcinoma of the bronchus. Minor clinical responses have also been seen in patients with ovarian cancer, breast cancer, bowel cancer and adenocarcinoma of the bronchus. It has been notable that clinical responses seem to occur in patients who had rather slowly progressing tumours which had been resistant to multiple other forms of therapy.

**High dose and conventional dose ellipticine (E) in advanced ovarian and breast cancer**

A.L. Harris[1], S. Raju, S. Ziddik, D. Spence, E. Wiltshaw, I.E. Smith & R. Levy

[1]*Clinical Oncology, Newcastle Hospital and Royal Marsden Hospital, Fulham Road, London*

E is a plant-derived intercalating agent that does not cause alopecia or neutropenia. The usual dose is $100\,mg\,m^{-2}$ i.v. weekly. Antibodies to the drug occur. Thirteen patients with advanced post-menopausal breast cancer resistant to endocrine therapy and adriamycin were treated on the weekly schedule. There were 2 partial responses (soft tissue, bone). Three patients stopped treatment because of severe weakness after 3 courses. All patients had dry mouths and 4 had severe vomiting. Seven patients with advanced ovarian cancer (Stage III, IV) resistant to *cis* platinum received $300\,mg\,m^{-2}$ E 3-weekly. All patients had normal plasma creatinine before treatment. Two days after E, creatinine rose to $132–304\,\mu mol\,l^{-1}$, peaking at day 2–8. Urine NAG and $\beta2$ microglobulin rose 2–3-fold. Thus nephrotoxicity prevented the use of the high dose regimen. Antibody titres to E were measured before each course of treatment and ranged from 1/16 to 1/1024 in 10/20 patients. One patient developed severe intravascular haemolysis 1 hour after the 5th course of E ($100\,mg\,m^{-2}$) (antibody titre 1/64). Antibody titre was not predictive of haemolysis.

Although E is active in adriamycin resistant breast cancer, the side effects are severe and limit its utility.

**LHRH analogue treatment for adenocarcinoma of prostate**

J.M. Allen, J.P. O'Shea, G. Williams & S.R. Bloom

*Department of Medicine and Urology, RPMS, Hammersmith Hospital, Du Cane Road, London, W12 0HS, U.K.*

Twelve patients with advanced progressive adenocarcinoma of prostate have been treated for at least 3 months with a long acting decapeptide analogue of gonadotrophin releasing hormone (LHRH), ICI 118, 630. Five patients had either previously failed to respond or relapsed on conventional endocrine therapy. Of these, two have achieved remarkable symptomatic improvement being able to withdraw from narcotic analgesia. The remaining three patients failed to respond to the analogue. Seven patients with histological grade III tumours received the LHRH analogue as first choice. One of these patients presented at an advanced stage with a pathological fracture of the humerus, and has shown only slight improvement. The remaining 6 patients have all responded to treatment in terms of symptomatic relief of pain, clinical regression of tumour and normalisation of tartrate labile acid phosphatase. Endocrine assessment in these patients has shown a rise in serum LH, FSH and testosterone over the first 5 days of treatment. Levels of gonadotrophins and testosterone are significantly suppressed two weeks after starting treatment and remain low thereafter.

The LHRH analogue appears a potent treatment for endocrine responsive tumours such as adenocarcinoma of prostate. It offers advantages over conventional therapy such as stilboestrol as it appears a more specific and effective suppressant of testosterone and gonadotrophins.

**VP16-213 infusions for the treatment of metastatic lung cancer**

W.P. Steward, N. Thatcher, W. Shiu, J. Wagstaffe & D. Crowther

*C.R.C. Department of Medical Oncology, Christie Hospital, Manchester, U.K.*

Twenty-five patients with extensive stage lung cancer (9 small cell, 5 squamous cell, 10 undifferentiated and 1 adenocarcinoma) were given 24 h infusions of VP16-213 at a dosage of $600\,mg\,m^{-2}$ repeated three weekly. Visceral metastatic sites included liver (ten patients) and bone (four patients). Karnofsky performance before treatment was a median of 60% with a range of 40 to 80. Eight patients had previously been given radiotherapy.

Seventy-three infusions were administered to the total patient group. A median of 3 courses (range 1–6 courses) was given. Three patients received only one course and three had five courses or more.

Two partial responses were obtained, 6 patients had static disease, and 17 patients progressed despite treatment. In 15 of the patients there was an improvement in the Karnofsky performance. The median survival from the start of treatment was 2 months (range 1–18 months).

Five patients had severe myelotoxicity and there was one death due to septicaemia.

It is considered that VP16-213 administered at this dosage by infusion is not an effective agent for the treatment of metastatic lung cancer.

### Primary therapy for poor prognosis small cell carcinoma of bronchus (SCCB) with vindesine and VP-16-213

A. Gregor, M.A. Cornbleet, S.G. Allan & J.F. Smyth

*Department of Clinical Oncology, Western General Hospital, Edinburgh*

Despite significant improvement in the overall management of patients with SCCB, the prognosis for patients presenting with hepatic or CNS metastases $\pm$ poor performance status (PS) is particularly poor. To evaluate a new drug combination, we have entered 31 previously untreated patients with biopsy proven SCCB, unsuitable for intensive multimodality therapy into this phase II study. Entry criteria included age up to 75 and PS$\leqq$4. Following staging with marrow aspiration, bone, and brain scans and liver ultrasound, 23 patients had extensive disease (12 liver, 8 marrow, 2 brain) and 17 patients had multiple sites involved. Vindesine 3 mg m$^{-2}$ day 1, and VP-16-213 120 mg m$^{-2}$ days 1–3 were given i.v. every 21 days $\times$ 6. Twenty-eight patients—19 men, 9 women, mean age 63.7 (range 44–73)—are evaluable. 19/28 (68%) patients responded (3 complete), all seen by 9 weeks. 100% limited and 55% extensive disease patients responded, including 6/12 with hepatic involvement. Apart from alopecia (all patients) toxicity was mild. Only one patient developed haematological toxicity, WHO grade III. Gastrointestinal and neurotoxicity were minimal. Median survival is 3.5 months for non-responders and 5+ (not yet reached) for responding patients. This combination provides effective, non-toxic palliation in a substantial proportion of SCCB patients presenting with poor prognostic features.

### Studies on the drug sensitivity of human glioma cell lines in culture

S. Merry, R.I. Freshney & S.B. Kaye

*Department of Clinical Oncology, University of Glasgow, G12 9LY*

Continuous cell lines have been established from 6 individual cases of human glioma, and their sensitivity to 6 drugs (actinomycin D, adriamycin, 5-fluorouracil, melphalan, vincristine and VP16-213) has been established. Cells were seeded onto microtitre plates and, after 72 h, were exposed to drugs for a further 72 h followed by a recovery period of 120 h. Cell number at the end of this period was determined by the incorporation of labelled amino acid into the perchloric acid insoluble material derived from the cells (R.I. Freshney, J. Paul & I.M. Kane (1975), *Br. J. Cancer*, **31**, 89) and by Coulter counting. The range of $ID_{50}$ values obtained for each drug was: actinomycin D $<6\times10^{-6}$–$4\times10^{-8}$ M; adriamycin $6\times10^{-8}$–$5\times10^{-6}$ M; 5-fluorouracil $5\times10^{-6}$–$1.7\times10^{-4}$ M; melphalan $8\times10^{-7}$–$2.5\times10^{-4}$ M; vincristine $6\times10^{-9}$–$3\times10^{-5}$ M and VP16-213 $3\times10^{-7}$–$1.9\times10^{-4}$ M. Cross-sensitivity and resistance was seen for 3 drugs i.e. actinomycin D, adriamycin and VP16-213, but not for other drugs.

Incubation of cells with labelled drug has shown that the net uptake of actinomycin D proceeds at a rate at least four times as great in a sensitive cell line as it does in resistant cell lines. Further studies on transport using radiolabelled adriamycin are in progress. The evidence to date suggests that patterns of cross-resistance seen in several animal tumours also apply to some human tumours in culture. The underlying mechanism may relate to changes in drug influx and/or efflux as has been demonstrated in animal tumours, and may provide a rational basis for therapy aimed at circumventing the tumour cell resistance which is observed clinically.

**Culture and drug sensitivity of human lung tumours in the clonogenic assay system**

A.P. Simmonds[1], P.S. Hamilton[1] & K.G. Davidson[2]

[1]*Biochemistry and* [2]*Cardiothoracic Surgical Unit, Royal Infirmary, Glasgow*

Forty eight samples of lung tumours obtained at thoracotomy have been received for clonogenic assay. Of the 44 presently assessed, 39 have positive pathology and have been cultured in the double layered agar system with mouse spleen conditioned medium. Drŭg sensitivity has been evaluated using 1 h exposures to the drugs vindesine and *cis*-platinum, both in current use for inoperable non small cell cancer of the lung. Assessment of *in vitro* response was made using the parameters of Salmon *et al.* (Cloning of Human Tumour Stem Cells: 1980: Alan R. Liss) with a minimum of 30 colonies per plate accepted in controls for significant drug results. Predominant in tumour pathology were squamous (22/39), the remainder being adenocarcinomas, with the exception of 1 sarcoma and 2 oat cell carcinomas. Twenty-six of the 39 samples were cultured successfully (66%). Surprisingly, in view of tumour site, contamination rate was low (2/39). Plating efficiencies ranged from <0.01% up to 0.16% but were predominantly low. Consequently, significant drug results were obtained in only 10 cases, although a further 4 samples had high plating efficiencies but insufficient cell yield. Sensitivity to vindesine was exhibited in 7/10 (70%) of samples, the remainder being resistent. Strikingly, however, only 1/10 (1%) showed sensitivity to *cis*-platinum, 3 being intermediate, and the remainder 6/10 (60%) were resistant. The sample sensitive to *cis*-platinum was also sensitive to vindesine. No relationship was observed between tumour pathology and growth patterns or drug response. In addition, tests to establish the need for conditioned medium and comparison of methods of disaggregation were equivocal.

**Results obtained in short-term assay using nucleotide incorporation for the *in vitro* prediction of chemosensitivity of ovarian tumours**

A. Wilson, C. Taylor, B. Laher, C. Newman, C. Ford & A. Howell

*Department of Obstetrics & Gynaecology, Withington Hospital, Manchester U.K.*

Cell suspensions obtained from human ovarian tumours were exposed to various concentrations of adriamycin (ADM) for 3 h, and the effect of the drug was quantified using depression of [$^3$H]-uridine incorporation. The methodology was essentially that described by Volm *et al.* (*Eur. J. Cancer* (1979), **15**, 983), and the aim of the investigation was to confirm the West German group's findings, particularly that ADM sensitivity *in vitro* indicates a chemoresponsive tumour. Using a cut-off point of <60% of control at 20 $\mu$g ml$^{-1}$ of ADM 10/23 (43%) tumours were sensitive. Clinical response to treatment with *cis*-platinum correlated with *in vitro* sensitivity to ADM in 5/8 (62.5%) cases. When P388 cell lines which were sensitive (S) and resistant (R) to ADM were tested in the same way the assay did not show a difference between the cell lines, nor could a dose-response curve be obtained. Non-specific binding (NSB) of radioactivity to filters and low viabilities were found to be responsible for this. When the assay was modified to eliminate NSB and maintain viability, reproducible dose-response curves were obtained which showed a difference between the S and R cell lines. Use of the modified assay for human tumours has demonstrated its increased sensitivity. In validation of the assay results of technical artefacts suggests that the level of correlation obtained with the original methodology is not significant, although of the same order as that reported by other studies.

**DTIC: an appropriate clinical alternative**

D.E.V. Wilman

*Department of Biochemical Pharmacology, Institute of Cancer Research, Sutton, Surrey*

Over the years considerable efforts have been made in the search for a suitable clinical alternative to DTIC. Whilst the initial reason for this was to overcome DTIC's photolability, improved clinical procedures and newer photochemical investigations have now rendered the problem of lesser importance.

Our own investigations have produced an extensive structure-activity series of photostable aryltriazenes. This has led to the conclusion that the necessary requirements for antitumour activity in this class of compounds are a carrying group at $N^1$, a methyl group at $N^3$ and a readily metabolisable group also at $N^3$. Triazenes of this type may then undergo metabolism to produce a cytotoxic monomethyltriazene.

Xenograft testing of selected examples of these compounds has shown them to have potent activity against a Grade IV astrocytoma. In particular, 1-(4-carbamoylphenyl)-3-methyl-3-pentyltriazene (CB10-350) is active in this system when the tumour is transplanted in the flank or intra-cerebrally, whereas DTIC has no effect on the intra-cerebral tumour. The NCI have demonstrated similar compounds, especially 1-(4-carbamoylphenyl)-3-ethyl-3-methyltriazene (CB10-335), to have marked activity against colon and lung tumour xenografts. Experimental antitumour activity alone is not sufficient for the selection of a drug for Phase I clinical trial. The high lipophilicity of the 4-carbamoylphenyltriazenes, a necessity for penetration of the blood-brain barrier, makes for difficulties in formulation. Recent metabolic results (C.J. Rutty, pers. comm.) suggest that a dialkyltriazene may not be a suitable clinical candidate, but rather an analogue not requiring oxidative metabolism would be more effective.

### Synthesis and properties of a new bicyclic antitumour agent (CCRG 81010; M & B 39565)

M.F.G. Stevens[1], J.A. Hickman[1], R. Stone[1], N.W. Gibson[1] and E. Lunt[2]

[1]*CRC Experimental Chemotherapy Group, Department of Pharmacy, University of Aston in Birmingham and* [2]*May & Baker Ltd., Dagenham, Essex*

CCRG 81010 or M&B 38565 is a bicyclic heterocycle with broad spectrum antitumour activity. The novel framework imidazo[5,1-d]-1,2,3,5-tetrazine ring-system evolved following long-term chemical investigations on cyclic and acyclic moeities bearing NNN bonds and bicyclic systems with bridgehead N atoms; the crucial peripheral substituents were identified following recent extensive biochemical investigations on the mode of action of the clinically-used agents DTIC and BCNU.

The drug is synthesised by interaction of 5-diazoimidazole-4-carboxamide and 2-chloroethylisocyanate in ethyl acetate at 30° in the dark (95% yield) and has pronounced activity against the NCI panel of mouse tumours (L 1210, P 388, LL, C 38 and B 16). The inhibitory activity against advanced solid mouse tumours (M 5076, ADJ/PC6A) is particularly noteworthy and the drug inhibits both the primary LL tumour and its pulmonary metastases and is markedly superior to DTIC in both cases. The drug shows some similarities to DTIC and BCNU in its spectrum of activity but exhibits distinctive properties *in vivo* and *in vitro*. The intact drug is not an alkylating agent: it has curative properties against the TLX5 lymphoma, a tumour insensitive to alkylating agents of the β-chloroethylamine type, and L 1210/cyclophosphamide. The drug is stable at acid pH values but degrades in alkaline conditions. At pH 7.4 in phosphate buffer the drug has a $t_{\frac{1}{2}}$ of 98 min.

CCRG 81010
M & B 39565

### The relationship between melanogenesis and response to chemotherapy in human malignant melanomas xenografted into athymic mice

S. Sparrow

*Toxicology Unit, Medical Research Council Laboratories, Woodmansterne Road, Carshalton, Surrey*

Five human malignant melanomas were serially passaged in nude mice and only minor morphological changes noticed at different passages. One tumour was partially pigmented (0.05% of cells showing melanin by light microscopy) and four were amelanotic. Tumour volume doubling times varied from 7–10.5 days. The xenografts were treated with CB10-286 (a dimethyl phenyl triazene) by injecting the mice intraperitoneally at a dose rate of 40 mg $Kg^{-1}$ (ten treatments in a period of 4 weeks). The partially pigmented tumour showed a total inhibition of growth and became very melanotic (7.5–33% of cells with melanin). This was accompanied by an increase in cell size and a loss of structural morphology. No mitotic figures were seen in these treated tumours. Two of the amelanotic tumours also showed some reduction in growth rates as a result of treatment. This was accompanied in both cases by the appearance of small numbers of pigmented cells and a reduced mitotic index. The two remaining amelanotic tumours showed no response to treatment either in terms of growth rate or the appearance of pigmented cells.

A number of workers have demonstrated an inverse relationship between proliferation and pigmentation of melanoma cells *in vitro* (Sheridan and Simmons (1981), *Br. J. exp. Path.*, **62,** 289) but the biosynthesis of melanin is still unclear. A number of promoting and inhibiting factors have recently been proposed (Pawelek *et al.* (1980), *Nature,* **286,** 617) and a relationship between these factors and tumour growth could have profound implications for the treatment of malignant melanoma.

### 17β oestradiol modulates the response of human breast cancer cells to methotrexate

R. Clarke[1], H.W. van den Berg[2] and D.G. Kennedy[1]

[1]*Departments of Biochemistry and* [2]*Therapeutics and Pharmacology, The Queen's University of Belfast, Northern Ireland*

The modifying influence of 17β oestradiol (E2) on the antimetabolic and growth inhibitory effects of methotrexate (MTX) has been investigated in two human breast cancer cell lines which differ in their steroid hormone receptor content and oestrogen responsiveness. The MDA-MB-436 cell line synthesises low levels of oestrogen receptor and is unresponsive to the hormone. In this cell line, E2, ($10^{-10}$–$10^{-6}$M) tended to reverse the antimetabolic action of MTX, an effect which became significant at $10^{-6}$M E2. $10^{-6}$M E2 also reduced the anti-proliferative action of MTX such that approximately twice the concentration of MTX was required to inhibit cell proliferation to the same extent as was observed following exposure to MTX alone. This partial reversal of response to MTX correlated with a 20% reduction in steady state intracellular drug concentration when cells were exposed to $10^{-7}$M $^3$H-MTX in the presence of $10^{-6}$M E2. In contrast to these results, E2 ($10^{-8}$–$10^{-6}$M) potentiated the action of MTX towards the MCF-7 cell line which synthesises high levels of the hormone receptor and is oestrogen responsive.

We conclude that the influence of E2 on the cytotoxicity of MTX is dependent on the steroid receptor status of the target cell. This may have important consequences for combined hormone–drug therapy of human breast cancer consisting of a population of tumour cells heterogeneous with respect to steroid hormone responsiveness and receptor content.

### Effects of adriamycin on the membrane potential of L1210 cells

S.B. Chahwala, J.A. Hickman & R.G. Grundy

*CRC Experimental Chemotherapy Group, Department of Pharmacy, University of Aston, Birmingham B4 7ET*

The antitumour activity of adriamycin has been related to its intercalating ability in double-stranded DNA (Di Marco *et al.* (1974), *Antibiot,* **3,** 107). In addition to its antitumour activity, adriamycin is cardiotoxic, eliciting ouabain like effects on the heart. Recently targets other than DNA, in particular the cell membrane have been proposed to explain the cytotoxic and cardiotoxic properties of adriamycin (Schwartz (1979), *Adv. Cancer Chemother.*, **1,** 1). In an effort to extend the studies of adriamycin induced membrane perturbations, we have looked at its effects on the membrane potential (Ψ) of L1210 cells. The (Ψ) was chosen because changes in (Ψ) may reflect changes in ion flux across the membrane and also because (Ψ) changes have been implicated with cell division (Sachs *et al.* (1974), *Exp. Cell. Res.*, **83,** 362). Measurements of (Ψ) were obtained by following the accumulation of the lipid-soluble cation triphenylmethylphosphonium ($TPMP^+$) into L1210 cells. The resulting (Ψ) was −80 mV, comparable to values reported by (Kiefer *et al.* (1980), *Proc. Nat. Acad. Sci. U.S.A.*, **77,** 2200) for murine spleen lymphocytes. The cytotoxic concentration of adriamycin ($2.6 \times 10^{-7}$M) which gave 90% cell kill after 1 h incubation, depolarised (Ψ) by 8 mV after 1 h. The known cardiac glycoside ouabain ($Na^+$-$K^+$ ATPase inhibitor) at 1 mM depolarised (Ψ) by 12 mV. Furthermore, in the assay for $Na^+$-$K^+$ ATPase activity in the L1210 cells, adriamycin ($2.6 \times 10^{-7}$m) and ouabain (1 mM) respectively caused 13% and 50% inhibition after 1 h. In conclusion, it is seen that the activity of the $Na^+$-$K^+$ ATPase does not make a significant contribution to the (Ψ) of L1210 cells and that the observed differences in activity between adriamycin and ouabain contradicts a proposed common mechanism (Gosalvez *et al.* (1979), *Cancer Res.* **39,** 257).

### Metabolic effects of cytotoxic drugs in tumour-bearing animals

G.E. Raines[1], J.C. Willox[1], G.B.S. McDonald[1], J. Irvine[1], D. Doyle[2] & K.C. Calman

[1]*Department of Clinical Oncology, University of Glasgow, Glasgow and* [2]*Department of Neuropathology, Southern General Hospital, Glasgow*

Aspects of host metabolism and morphology were studied in Wistar rats bearing a sensitive cachectic Walker 256 tumour receiving an $LD_{10}$ dose of either *cis*-platinum or cyclophosphamide. Animals were divided into four groups. Group 1 had tumour alone, Group 2 had tumour and drug, Group 3 had drug alone whilst Group 4 had neither tumour nor drug. Body weight and tumour size were monitored during the study and the final tumour weight noted on culling when blood was withdrawn for ketones and albumin determinations. Groups were subdivided for histochemical and e.m. assessment which were performed on the heart, liver, kidney and gastrocnemius and soleus muscles. Total body nitrogen and water content were measured in intact carcases.

The most notable observations were (i) the decreasing tumour size in Group 2 following drug administration, (ii) the evident loss of body nitrogen in Group 1 and maintenance in Group 2, (iii) the reduction of blood ketone levels in Group 2, (iv) the altered albumin concentrations between Groups 1 and 2, and (v) the abnormal histochemical and e.m. results in the different groups.

In conclusion, therefore, the tumour alone group displayed a number of distinct metabolic and morphological abnormalities, some of which were alleviated or reverted to normal with drug administration, and that the drugs themselves affected host metabolism and morphology.

### Studies on the hepatotoxicity of the antitumour agent N-methylformamide in mice

H. Whitby, A. Gescher & L. Levy[1]

*CRC Experimental Chemotherapy Research Group and* [1]*Department of Occupational Health and Safety, University of Aston, Gosta Green, Birmingham*

The antitumour agent, N-methylformamide (NMF), which is currently undergoing phase 2 clinical trial, has been shown to be hepatotoxic in man (Myers *et al.* (1956), *Cancer,* **5**, 949) and in rats (Lundberg *et al.* (1981), *Toxicology,* **22**, 1). We have investigated the effect of NMF in livers of male Balb/C mice *in vivo* and *in vitro*. Ten out of twelve livers from mice injected with NMF 400 mg $kg^{-1}$ i.p. for 5 days (the optimum antitumour dose regimen) showed evidence of toxicity. Histopathological examination of 7 of these livers showed areas of varying degrees of necrosis. Total glutathione (GSH) in five of the livers of treated animals was estimated, as changes in GSH status after single doses of NMF have been reported previously (Gescher *et al.* (1982), *Br. J. Cancer,* **45**, 843). No significant differences in liver GSH were noted between NMF mice (7.02 $\mu mol\,g^{-1}$ liver) and control mice (6.93 $\mu mol\,g^{-1}$ liver). Incubation of isolated mouse hepatocytes with 7 mM NMF resulted in a significant reduction in total intracellular GSH after 80 min when compared with controls. GSH levels were reduced by $64.6 \pm 18.9\%$ ($n = 11$). Lipid peroxidation, as measured by malondialdehyde formation, was markedly increased on incubation of hepatocytes with 7 mM NMF for 180 and 240 min. Such delayed changes tend to suggest that a NMF metabolite may be responsible. The *in vitro* biochemical effects may be relevant to the *in vivo* hepatotoxicity produced by NMF. However, the different effects on GSH levels *in vitro* and in the multidose *in vivo* experiment suggest that other mechanisms, not implicating a reactive metabolite, may also be involved.

### Benznidazole—chemosensitization and pharmacokinetics

P. Workman, P.R. Twentyman, F.Y.F. Lee, M.I. Walton, L.N. Owen[1] & R.A.S. White[1]

*MRC Clinical Oncology Unit and* [1]*Department of Clinical Veterinary Medicine, Cambridge*

Benznidazole (BENZO) is a lipophilic analogue of misonidazole which exhibits greater enhancement of antitumour effects of the nitrosourea CCNU, particularly when the sensitizers are compared at low doses (Workman and Twentyman (1982), *Br. J. Cancer,* **46**, 249). Using BENZO doses which give plasma concentrations which should be achievable in man, we have evaluated the therapeutic gain for its combination with CCNU using the KHT tumour in C3H mice. The endpoints used were regrowth delay for tumour, and LD50 and depression of peripheral white cells for normal tissues. The effects were related to pharmacokinetics, using HPLC assay of BENZO, CCNU and their metabolites.

Using a single dose of $0.3\,mmol\,kg^{-1}$ of BENZO (ip) 30 min before CCNU (ip) the dose modification factor (DMF) for tumour response was 1.5–2.0 compared with 1.2–1.3 for white cells and LD50, thus demonstrating a therapeutic gain for the combination. For this dose the peak plasma and tumour concentrations were about 0.1 mM and the elimination $t_{\frac{1}{2}}$ about 2 h. In experiments where plasma concentrations of 0.1 mM were maintained for 16 h the tumour DMF was no greater than with the single dose. BENZO increased the exposure to CCNU by a factor of 2. Thus, as for misonidazole, the chemosensitization mechanism involves changes in CCNU pharmacokinetics (Lee and Workman, these proceedings). The BENZO $t_{\frac{1}{2}}$ was 4–5 h in sheep (iv) and 6–12 h in dogs (iv or ip). Plasma and tumour concentrations of 0.1 mM were readily obtainable. Tumour/plasma ratios of 50–100% were seen in transplantable mouse tumours and spontaneous dog tumours. As a result of these promising chemosensitization and pharmacokinetic data a Phase I clinical trial of BENZO plus CCNU is now in progress.

### Conjugation of methotrexate to antibody via an albumin carrier confers selective cytotoxicity *in vitro*

M.C. Garnett, M.J. Embleton, E. Jacobs and R.W. Baldwin

*Cancer Research Campaign Laboratories, University of Nottingham, Nottingham HG7 2RD*

Methotrexate (MTX) was linked using stable covalent bonds to a monoclonal antibody (α791T/36) against an osteogenic sarcoma cell line via Human Serum Albumin (HSA) to obtain a conjugate with a high molar ratio of drug to antibody and specificity of action.

A conjugate of empirical formula $(MTX_{32}-HSA)_{1-3}$-α791T/36 was synthesised which retained 25% of the original antibody binding activity. The effectiveness of the conjugate *in vitro* was assessed against target cells of previously determined reactivity towards α791T/36 using inhibition of $^{75}$Se-Selenomethionine uptake as a measure of cytotoxicity. In a chronic 24 h exposure of drug to target cells, methotrexate substituted HSA was 120–360 fold less toxic than free methotrexate. The complete conjugate was as toxic as free methotrexate against reactive target cells but 40-fold less cytotoxic against non-reactive target cells. In a competition cytotoxicity test against free antibody, the cytotoxicity was shown to be dependent on antibody binding. A further variation of the cytotoxicity test in which conjugate of free drug was incubated with target cells for fifteen minutes followed by washing to remove unbound material demonstrated that the conjugate was capable of killing reactive but not non-reactive target cells under these conditions. Although the conjugate was 7–30 fold more active than free methotrexate under these conditions, this was at higher concentrations than in the chronic assay.

### Immunohistochemical localisation of milk fat globule antigens in routine breast biopsy material

N. Berry, D.B. Jones, N. Kirkham, J. Smallwood, I. Taylor & J. Taylor-Papadimitriou

*Departments of Pathology & Surgery, Southampton General Hospital and Imperial Cancer Research Fund*

Two monoclonal antibodies, HMFG-1 (1.10.F3) and HMFG-2 (3.14.A4), identify antigens of the human mammary milk fat globule and stain breast biopsy tissue (Papadimitriou *et al.* (1981), *Int. J. Cancer*, **28**, 17; 21). An indirect immunoperoxidase staining technique has been used to determine the distribution of HMFG-1 and HMFG-2 antigen in formalin fixed, paraffin embedded sections of breast from 50 females. Nine of the specimens were histologically normal. In these, HMFG-1 and HMFG-2 stained both secreted material within, and the luminal surface of cells lining many of the ducts and tubules. Intracellular staining was occasionally seen in these cells. HMFG-2 staining was stronger than HMFG-1. Six cases of benign breast disease showed the same staining pattern as normal breast. Thirty-five cases of breast carcinoma were also stained, comprising 29 ductal, 2 lobular, 2 mixed ductal and lobular, 1 medullary and 1 carcinoid. The staining pattern of the carcinomas varied and could be crudely related to Bloom's grade (Bloom, H.J.G. and Richardson, W.W. (1957), *Br. J. Cancer*, **11**, 359). Tumours showing much tubule formation gave a staining pattern resembling that observed in normal breast. Tumours with little tubule formation showed either focal or diffuse intracellular staining patterns. The pattern of staining with HMFG-1 and HMFG-2 was the same in all sections although HMFG-2 was generally stronger than in HMFG-1. In 2 cases HMFG-1 stained tissue not stained by HMFG-2. The one carcinoid stained with neither antibody staining did not correlate with histological type of tumour but may relate to oestrogen receptor status. The staining of cells isolated from fresh breast tumour tissue has also been studied using these antibodies in relation to the staining pattern of the original tumour biopsy tissue.

### Abdomino-pelvic C.T. scanning in the management of carcinoma of the ovary. A possible alternative to second look laparotomy

G. Blackledge, R. Johnson, B. Eddleston & D. Crowther

*Queen Elizabeth Hospital Birmingham, Christie Hospital Manchester*

121 CT scans were obtained in 75 women with ovarian cancer; 108 of these were of the abdomen and pelvis and 13 of the pelvis only. In 48 cases pelvic CT was performed within 3 weeks of surgery confirming the operative findings in all but 6. In the abdomen, CT identified intra-hepatic deposits and minimal ascites not seen at surgery; further small peritoneal deposits (<1.5 cm) were found at surgery but not seen by CT. CT was superior to clinical examination in all instances and was undoubtedly helpful in assessing the feasibility of a successful repeat laparotomy. More recently studies suggest that unless second look laparotomy can debulk residual tumour its function is limited. CT scanning may be able to replace laparatomy in the assessment of disease post chemotherapy and minimise the number of patients requiring further surgery.

### Gallium scanning by conventional imaging (Ga-C) and emission computed tomography (Ga-ECAT) in the pretreatment evaluation of lung cancer

D.L. Broughton[1], R.C.F. Leonard[2], T. Crake[1], C.J. Gibson[1], S.J. Pearce[1]

[1]*Department of Medicine and Regional Medical Physics, Dryburn Hospital, Durham and* [2]*Department of Clinical Oncology, University of Edinburgh*

In a prospective, coded study the clinical utility of gallium imaging was assessed in 31 consecutive patients presenting with radiological (CXR) evidence of lung cancer. The study assessed (1) the sensitivity of Ga-C and Ga-ECAT imaging in relation to the location and histopathology (HPG) of the tumour, and (2) the value of Ga-C and Ga-ECAT in detecting mediastinal disease. Following bronchoscopy (B) imaging was performed 72 hours after a dose of 160 MBq of gallium citrate. ECAT images were interpreted from both rotating pictures and slice sections. The results showed (1) Gallium imaging was accurate in all HPG subgroups (16 squamous, 6 adeno, 3 large cell, 3 small cell, 3 unclassified). Only one adenocarcinoma did not show a clear primary. (2) Site of the primary on gallium imaging correlated accurately with the radiographic findings and there was no clear variation of intensity of image with size and location. (3) The mediastinum was more often abnormal on Ga-C than CXR, B or ECAT. Twelve patients had thoracotomy, ten had normal mediastinal biopsies (M−) of whom two had been equivocal (ME) on CXR. All were Ga-C M−. Two patients had positive mediastinal node biopsies (M+) who were M− ME on CXR but ME, M+ on Ga-C. Both were M− on Ga-ECAT. (4) In 13 non-surgical patients with a high probability of M+ (B.M+ and/or CXR M+) 12 had Ga-C M+, one ME; 10 had Ga-ECAT M+. In 6 with a low probability of M+ (both B.M− and CXR M−) Ga-C and Ga-ECAT gave discordant results. It is concluded that Ga-C is accurate in detecting mediastinal disease but Ga-ECAT imaging is not as accurate and not a useful adjuvant to gallium imaging. Ga-C is a valuable staging procedure in the pretreatment assessment of lung cancer.

### Does a factor in cancer sera change oxygen utilisation of peripheral blood lymphocytes?

A.G. Paterson, A.D. Grimshaw & D.J.T. Webster

*University Department of Surgery, Welsh National School of Medicine, Heath Park, Cardiff*

We previously reported that lymphocytes from patients with cancer have an increased consumption of oxygen. We have now attempted to define whether this observation is due to an intrinsic function of the lymphocyte or due to a factor present in serum of such patients. The results are shown in the table. Papain causes levels of oxygen consumption to return to normal when incubated at 37°C but has no effect at 4°C.

| | *Lymphocyte $O_2$ consumption* | *After incubation papain 37°C* | *After incubation papain 4°C* |
|---|---|---|---|
| Control (9) | 6.45±0.65 | 5.46±1.52 | 6.31±0.67 |
| Breast cancer (10) | 9.48±1.79 | 6.41±1.36 | 10.54±2.65 |
| GI cancer (9) | 9.80±2.3 | 6.38±1.32 | 9.67±1.95 |

($\mu l \times 10^{-5} sec^{-1}$)

When normal lymphocytes are incubated with serum from patients with cancer oxygen consumption in 9 patients rose from $6.34 \pm 0.90$ to $9.65 \pm 1.67$. No change was recorded in oxygen consumption when serum from patients with normal levels of oxygen consumption were incubated with these lymphocytes.

These results suggest that the cause of increased oxygen utilisation by lymphocytes from patients with cancer relates to a serum factor rather than being an intrinsic function of the lymphocytes.

### Intratumour heterogeneity of thymidine labelling index in primary breast cancer: The reason for its failure as a prognostic indicator

M. Lambert

*Department of Surgery, University Hospital of South Manchester, Manchester 20*

Tritiated Thymidine Labelling Indices (LIs) have been determined from specimens of 22 primary human breast cancers, using the hyperbaric *in vitro* method of Meyer and Bauer (1975). Each fresh specimen, typically measuring 2 x 2 x 4 mm, was cut crudely into 0.5 mm thick slices, which were incubated, processed and counted separately. An LI was determined for each. A total of 2000–2500 tumour cells was counted in each microscopic section in order to determine the LI. Repeated counts upon each microscope slide showed that the LI determined by this means was consistent, with only one out of 22 repeat counts differing significantly ($P<0.05$) from first count.

When the LIs of adjacent 0.5 mm slices from each specimen were compared, there was seen to be a significant difference ($P<0.05$) in 19 out of 22 tumours, the significance of the difference ranging from $P=0.05$ to $P=0.00001$ (a four-fold difference in LI) in these 19 pairs of adjacent slices.

Clearly there is great heterogeneity of proliferative activity within a given primary breast cancer. LI, or any other cell kinetic index based upon evaluation of a *single small sample* in human primary breast cancer is subject to considerable *sampling error* and is unlikely therefore to be of significant prognostic value.

### The metastatic pattern of infiltrating lobular carcinoma of the breast

M. Harris, A. Howell, M. Chrissohoou, M.J. Hudson, R. Swindell & R.A. Sellwood

*Departments of Pathology & Medical Oncology, Christie Hospital, Department of Surgery, Withington Hospital, Manchester, U.K.*

We have compared the metastatic patterns of infiltrating lobular carcinoma (ILC) and infiltrating duct carcinoma (IDC) of the breast using both clinical and autopsy data. Of 1082 patients with breast cancer 135 (12%) had ILC and 831 (77%) had IDC; 56 (41%) ILC's and 309 (37%) IDC's were metastatic. In addition the post mortem findings in 13 ILC's and 69 IDC's were compared.

The clinical data suggests that ILC is more likely to produce metastases in the opposite breast (25% *vs* 10%, $P<0.004$), in the leptomeninges (16% *vs* 0.3%, $P<0.0004$) and diffuse bone marrow involvement as judged by the proportion of positive bone marrow trephines (72% *vs* 27%, $P<0.0001$).

At autopsy gastric (46% *vs* 3%, $P<0.0001$), retroperitoneal (92% *vs* 9%, $P<0.0001$) and female genital tract (38% *vs* 0%, $P<0.0001$) metastases were significantly more frequent in ILC than IDC. Seven of 12 ILC patients with retroperitoneal spread had infiltration of the walls of the ureters causing hydronephrosis; a feature not seen in IDC.

ILC is considered to have a distinctive metastatic pattern compared with IDC.

### A further analysis of mortality from cancer of the prostate among nickel-cadmium battery workers by the method of regression models in life-tables

T.M. Sorahan & J.A.H. Waterhouse

*Cancer Epidemiology Research Unit, University of Birmingham, B15 2TH*

In a previous study of cancer morbidity among men employed in the manufacture of nickel-cadmium batteries (Kipling & Waterhouse, 1967), a statistically significant excess of cancer of the prostate was found ($E=0.58$, $O=4$, $P<0.01$). The present study updates and augments the original series to a cohort of 2559 males employed in the industry between 1923 and 1975 for at least one month. The OPCS provided information on the vital status of each individual on the closing date of the survey, 31 January 1981. For those who had died a death certificate was obtained with the underlying cause of death coded to the 8th revision of ICD. Occupational histories were described in terms of some 75 jobs: 8 with "high" and the remainder with "moderate" or "minimal" exposure to cadmium oxide (hydroxide). The method of regression models in life-tables (RMLT) was used to compare the estimated cadmium exposures (duration of "high" exposure employment) of male employees who died from cancer of the prostate with those of matching survivors in the same year of follow-up, whilst controlling for year and age at commencing employment. A large test-statistic was found but the relatively small number of deaths upon which this statistic was based make the estimation of a precise *P*-value difficult. Simulations carried out would indicate, however, that the statistic is significant at the 5% level. The number of deaths available for analysis was increased by considering those with cancer of the prostate mentioned in Part I or Part II of the death certificate. The effect of excluding the four previously reported cases was to reduce the significant positive statistic to a small non-significant negative statistic. Thus no *new* evidence has been provided which suggests an association between occupational exposure to cadmium oxide (hydroxide) dust and cancer of the prostate.

**Nitrosamine exposure in the rubber industry**

B. Spiegelhalder and R. Preussmann

*Institute of Toxiciology and Chemotherapy, German Cancer Research Center, Heidelberg*

Recent investigations (Fajen, J.M. *et al.* (1979), *Science*, **205**, 1262) of nitrosamines in the rubber industry indicate the widespread occurrence of considerable levels of nitrosodimethylamine and nitrosomorpholine.

In order to elucidate the origin and formation of nitrosamines in this industry, chemicals as well as the air in various areas were analyzed. All chemicals used for rubber compounding contain nitrosamines if they are derivatives of secondary amines, e.g. tetramethylthiurame, zinc-diethyldithiocarbamate or N-oxydiethylene benzothiazolylsulfonamide. Accordingly, variable concentrations of airborn nitrosamines could be detected at places where rubber products are manufactured or stored. The nitrosamines found correspond to the compounded chemicals. The original nitrosamine level in rubber chemicals is not high enough to explain the amounts found in rubber products and in air. Therefore additional nitrosation had to be considered. The responsible nitrosating agents are nitrogen oxides released from rubber chemicals (e.g. nitrosodiphenylamine) or from combustion gases. Preliminary results show that in most cases either by elimination of the nitrosating agent or by exchange or rubber chemicals nitrosamine levels in the working area can be drastically reduced.

**Pharmacokinetic studies in humans with CB3717 (N-(4-(2-amino-4-hydroxy-6-quinazolinyl)methyl) prop-2-ynylamino)benzoyl)-L-glutamic acid)**

D.L. Alison, D.R. Newell & A.H. Calvert

*Department of Biochemical Pharmacology, Institute of Cancer Research, Sutton, Surrey*

CB3717 is an antifolate acting by inhibition of thymidylate synthetase which began early clinical trials at The Royal Marsden Hospital in September 1981. Pharmacokinetic studies have been undertaken in 11 patients following a 1 h infusion of CB3717 and in 4 patients receiving a 12 h infusion at doses ranging between 100 mg m$^{-2}$–500 mg m$^{-2}$. In 9 of these patients the decay of drug levels in the plasma followed a biphasic pattern with an average $t\frac{1}{2}\alpha$ of 69.7 min (range 13.3–173.3 min) and an average $t\frac{1}{2}\alpha\beta$ of 556 min (range 228–1386 min). Peak plasma levels were linearly related to dose, falling within the range previously found to be therapeutic and cytotoxic in preclinical studies and the highest level recorded was 60 $\mu$g ml$^{-1}$ (122.7 $\mu$M). In patients receiving a 12 h infusion of CB3717 at doses of 300 mg m$^{-2}$, and 330 mg m$^{-2}$ peak levels of the drug measured 8, 15.5 and 36 $\mu$g ml$^{-1}$ (16.4, 31.7 and 73.6 $\mu$M) respectively and these were lower than the levels achieved by the same dose given over 1 hour. The mild reversible hepatic toxicity which occurs in approximately 50% of patients, demonstrated by rises in plasma transaminase levels, did not appear to be related to the rate of drug infusion, dose given or peak plasma level attained but did show correlation with the rate of clearance of the drug. 24 hour urine collections following 45 drug treatments showed the average urinary excretion of CB3717 to be 25% (range 4–75%). In addition, an incomplete faecal collection following a 5 day dose regimen in one patient yielded 12% of the total CB3717 given and some of the desglutamyl metabolite CB3751. This metabolite has not been detected in either plasma or urine samples and has been shown to be non cytotoxic and inactive as a thymidylate synthetase inhibitor. Post mortem tissue samples have been analysed from one patient who died 7 days after his second dose of CB3717 at a dose of $330\,mg\,m^{-2}$ and ~5% of the dose given was calculated to be present in the kidneys. Finally, protein binding experiments have demonstrated 99% plasma protein binding of CB3717.

**Evaluation of ECAT techniques for the measurement of gallium-67 uptake in the chest**

C.J. Gibson, R.C.F. Leonard[1] & J. Aikenhead

*Regional Medical Physics Department and [1]Department of Medicine, Dryburn Hospital, Durham, DH1 5TW*

The distribution of $^{67}$Ga-citrate uptake in patients with bronchogenic carcinoma may be an indicator of the extent of the disease, and therefore useful for staging and management. Gallium uptake is non-specific and is also used to detect foci of infection, hence the volume of distribution of gallium may represent inflammatory tissue as well as tumour. However, the overall shape of the uptake gives information about the three dimensional distribution of disease. We investigated the technique of emission computed axial tomography (ECAT) for the determination of volume and shape of gallium uptake. Irregular bags containing concentrations of gallium representative of tumours were imaged in an anthropomorphic chest phantom. Using a rotating gamma camera, ECAT images were obtained and the volume measured from the reconstructed sections compared with the known volume. We were able to show that tomographic imaging was possible even with the low concentrations used ($0.02\,MBq\,ml^{-1}$), and that accurate measurement of volume was possible for volumes above 200 ml. The use of a variable threshold for defining small volumes improved the accuracy, but required more complex data processing. The technique has been applied to clinical studies and examples are shown indicating the ability of ECAT to display the three dimensional distribution of gallium in lung cancer.

**The recovery of tumour cells from colorectal tumours and colonic lavage following centrifugation of cell suspensions on nycodenz columns**

H.C. Umpleby, B. Fermor, M.O. Symes & R.C.N. Williamson

*Department of Surgery, University of Bristol*

Cell suspensions were prepared from 9 colorectal carcinomas by digestion in collagenase + DNase. One ml of each cell suspension ($3.0–28.5 \times 10^6$, mean $16.2 \times 10^6$ cells) was placed on a separate density gradient Nycodenz column. Nycodenz (Nyegaard & Co., Oslo) was diluted with medium, $5\,mmol\,l^{-1}$ Tris HCl, $3\,mmol\,l^{-1}$ KCl, $0.3\,mmol\,l^{-1}$ $CaNa_2$ EDTA, $7.5\,g$ NaCl $l^{-1}$ distilled water. Each column comprised 4, 3 ml layers, Nycodenz, Nycodenz diluted 2:1 with medium, diluted 1:1 and diluted 1:2, in a conical centrifuge tube. Each tube was placed in a horizontal position for 45 min at room temperature prior to application of the tumour cell suspension. Each column was centrifuged at 1500 *g* for 45 min at room temperature. The cells obtained were localised in 4 bands of which the top contained most of the tumour cells. The % recovery of cells in this band was 0.8 to 22.5 (median 3.6) for the 9 colon tumours. With 4 of these tumours a count differentiating tumour cells, mononuclear cells and dead cells was made on a cell suspension diluted with 0.165% w/v trypan blue using a haemocytometer. The % of tumour cells was 82, 90, 95 and 100. Nineteen patients received a colorectal lavage with 500 ml of Hartmans solution. The resulting cell suspension was concentrated by centrifugation and applied to a Nycodenz column. The top band contained a median of $0.25 \times 10^6\,ml^{-1}$ (range nil–$100 \times 10^6$) viable tumour cells—there being 5 cases from which no tumour cells were recovered. In a further 18 patients the transected ends of the bowel were washed with Medium 199 and the concentrated cell suspension applied to Nycodenz column. Viable tumour cells were recovered from 12 of 18 cases, median $0.17 \times 10^6\,ml^{-1}$ range (nil–$4.5 \times 10^6$). Viability was confirmed by showing that the tumour cells incorporated fluorescein. Cytocentrifuge preparations demonstrated the presence of tumour cells in the top band in 7 of 13 cases following lavage and 8 of 14 cases after margin washing.

**Patterns of acute phase reactant proteins (APR's) and immune complexes (IC's) in lymphomas compared with other systemic diseases**

J.P. Pape, R.C.F. Leonard, S.J. Proctor, W.P. Carr, M.S. Jeffery & W.C. Dick

*Departments of Rheumatology, Clinical Oncology & Medicine, University of Newcastle upon Tyne*

Recently interest has been revived in the measurement of APR's in diseases characterised by systemic illness. In non-Hodgkin's lymphoma elevation of C-reactive protein (CRP) has been associated with poor prognosis. Generally, the function of the APR's is far from clear, although their evolutionary conservation implies an important physiological action. We have attempted to learn more about their possible functions by examining patterns of APR's in lymphomas, compared with normals and other inflammatory diseases. A second common abnormality in the chosen disease groups is the presence of circulating immune complexes. It has been hypothesised that individual APR's may be associated with IC's and these have been correlated in our studies, along with the complement factor C3. All APR's and C3 were measured by nephelometry.

The APR's examined were alpha-1-antitrypsin (a1-AT), alpha-1-acid glyco-protein (a1-AGP), alpha-2-macroglobulin (a2-M) and CRP.

The data shows that the CRP concentration in lymphomas are elevated compared with controls and differ from those of other disease groups. However, preliminary analysis indicates no clear correlation between the APR level and (1) clinical stage (2) histopathology (3) treatment status.

### Mechanisms of gastric distension following cytotoxic drugs. Effects of metoclopramide

B. Jones & M.G. Stone

*Richard Dimbleby Department of Cancer Research, St. Thomas's Hospital Medical School, London SE1 7EH*

Nausea and vomiting are important side effects of cytotoxic chemotherapy. Laboratory experiments involving emesis have necessarily been limited to large animals. The following experiments were performed using $C_3H$ mice in which severe gastric distension, but not emesis, occurs after a variety of cytotoxic drugs. The degree of distension was assessed by measuring weights and volumes of stomachs removed 24 h after cytotoxic drug administration. Marked distension occurred following single $LD_{50}$ doses of Neoplatin, CHIP (a platinum IV complex); Cyclophosphamide and lethal total body irradiation (15 Gy). The effect did not occur following Adriamycin or the platinum containing drug FLAP (1000 mg kg$^{-1}$ p.o.).

Single doses of Metoclopramide (0.5, 5 or 25 mg kg$^{-1}$ i.p.) produced a small non-dose dependent reduction in CHIP and Cyclophosphamide-induced distension within 1 h. Multiple doses of Metoclopramide (5 mg kg$^{-1}$ $\times$ 6) over the 24 h period did not yield any reduction in distension. This implies that pyloric sphincter closure is not responsible for distension. This is supported by finding no reduction in transit of small carbon beads (0.25–0.60 mm) between the oesophagus and the appendix in CHIP treated mice. Starvation effectively reduced the degree of distension by 73%.

This model suggests that the clinical benefits of high dose Metoclopramide following platinum drugs are due to central rather than direct gastric effects.

### Genetic damage initiated in somatic cells by oxygen-derived radicals produced by normal metabolic reactions

B.J. Phillips and T.E.B. James

*British Industrial Biological Research Association, Carshalton, Surrey*

Oxygen-derived radicals have been implicated in the mechanisms of action of ionizing radiation, some chemical carcinogens and tumour promoters, and certain anti-cancer agents. Oxygen radicals are produced by many normal cellular enzymes and are released by phagocytic cells in response to foreign substances. It has been suggested that under abnormal circumstances these physiological sources of radicals could cause or promote carcinogenesis. Cultured Chinese hamster ovary (CHO) cells were incubated with either xanthine plus xanthine oxidase or guinea-pig alveolar marcophages stimulated by various means. In both systems the generation of superoxide radicals was demonstrated. Severe chromosome breakage and sister chromatid exchange (SCE) were observed after exposure to conditions of high superoxide production. Chromosome breakage was abolished by superoxide dismutase (SOD) or catalase (CAT) implying that both superoxide and hydrogen peroxide were necessary for this effect. SCE was prevented only by catalase. The chromosomal effects studied are considered to be indicative of genetic damage which might underlie carcinogenesis.

### Promotion of N-nitrosodimethylamine-initiated bile duct carcinogenesis in the hamster by the human liver fluke *Opisthorchis viverrini*

D.J. Flavel & S.B. Lucas

*Department of Medical Helminthology, London School of Hygiene & Tropical Medicine and Department of Histopathology, St. Thomas' Hospital, London*

The liver fluke, *Opisthorchis viverrini* infects several million human beings in the north-east of Thailand (Wykoff *et al.* (1965), *J. Parasitol.*, **51**, 207) and appears to be an aetiological factor in the causation of bile duct carcinoma (Flavell (1981), *Trans. Roy. Soc. Trop. Med. Hyg.*, **75**, 814). In order to test the hypothesis that this parasite might act as a promoter of bile duct carcinogenesis, initiated by an exogenous carcinogenic agent, the following experimental groups of hamsters were set up. Group I animals received 50 *O. viverrini* metacercariae followed 41 days later (when biliary hyperplasia was well established) by 1.6 mg N-nitrosodimethylamine (NDMA), Group II received 1.6 mg NDMA followed 96 h later by 50 *O. viverrini* metacercariae, Group III received 1.6 mg NDMA only and Group IV 50 *O. viverrini* metacercariae only. Animals were left until natural death or were sacrificed when moribund and full post mortems performed. A total of five (10%) animals from Group I and nine (20%) from Group II developed intrahepatic bile duct carcinoma. None of the animals from Groups III and IV developed malignant bile duct tumours, though benign cystic cholangiomas were found in the majority of animals from Group III. These results show quite clearly that the parasite-mediated biliary epithelial cell proliferation is not a prerequisite during the NDMA-initiation phase of carcinogenesis. To the contrary, a higher tumour yield was obtained in animals initiated with NDMA before receiving parasites. These results therefore strongly suggest that the liver fluke, *Opisthorchis viverrini* acts as a promoter of NDMA-initiated bile duct carcinogenesis in the hamster, providing a model two- (or multi-) stage carcinogenesis system with some direct relevance to a malignancy of man.

### A multistage hypothesis for NDMA-initiated bile duct carcinogenesis in *Opisthorchis viverrini* infected hamsters

D.J. Flavell

*Department of Medical Helminthology, London School of Hygiene & Tropical Medicine, Winches Farm Field Station, St. Albans AL4 0XQ*

A multistage hypothesis implicating the liver fluke, *Opisthorchis viverrini* as both a direct and indirect promoter of NDMA-initiated bile duct carcinogenesis is presented here.

1. Initiation with a subcarcinogenic dose of NDMA produces latent tumour cells of biliary epithelial origin.
2. The parasite predisposes the human (Teoh (1963), *J. Path. Bact.*, **86**, 123) and hamster (Flavell, unpublished observation) hosts to colonisation of the biliary tree with species of gut microflora (pyogenic cholangitis). The presence of certain species of gut bacteria might result in the conversion of primary to secondary bile acids, the latter being established promoters of carcinogenesis (Reddy *et al.* (1978), *Carcinogenesis: A Comprehensive Survey*, Raven Press. pp. 453–464).
3. The parasites located in the intrahepatic bile ducts abrade and damage the mucosal lining of the duct wall (Flavell *et al.* (1980), *Acta Tropica*, **37**, 337) thus allowing for easier access of bile borne components to the membrane surface of the biliary epithelial cell which is subsequently promoted by secondary bile acids.
4. The growth of foci of NDMA-initiated and secondary bile acid promoted progenitor tumour cells is further accelerated through the proliferative stimulus exerted on the biliary epithelium by the parasite.

### Alkylation of hepatic Haem by diethylnitrosamine: Formation of N-hydroxyethyl protoporphyrin IX

I.N.H. White, A.G. Smith & P.B. Farmer

*Toxicology Unit, MRC Laboratories, Woodmansterne Road, Carshalton, Surrey*

Mice dosed with diethylnitrosamine form an abnormal green pigment in their livers. A procedure based on HPLC was used to separate and quantitate this product. Formation of green pigment was time and dose dependent and was induced by pretreatment of mice with either phenobarbitone or 3-methylcholanthrene.

The aetio-type absorption spectrum of the purified green pigment dimethyl ester suggested it to be a N-alkylated porphyrin. Desorption chemical ionisation mass spectrometry gave a protonated molecular ion $m/z = 635$, compatible with N-hydroxyethyl protoporphyrin IX. No conversion of N-ethyl protoporphyrin IX to N-hydroxyethyl protoporphyrin IX could be demonstrated *in vivo* or in microsomal systems *in vitro*. A reaction mechanism is proposed involving an initial $\beta$-hydroxylation of one ethyl substituent of diethylnitrosamine followed by the formation of a $\beta$-hydroxyethyl carbonium ion. The alkylation of other cellular macromolecules such as DNA by the proposed metabolite is being investigated.

### Activity of $O^6$-methylguanine-DNA repair protein in animal tissues in relation to their susceptibility to cancer induction by alkyl-nitroso-ureas

V.M. Craddock

*MRC Toxicology Unit, Woodmansterne Road, Carshalton, Surrey*

Much evidence suggests that initiation of cancer depends on replication of DNA containing carcinogen-derived adducts, i.e. it depends not only on the extent of the initial reaction of carcinogen with DNA but also on the rate of cell replication and on the persistence and therefore on the rate of repair of the relevant lesions. Measurement of repair capacity *in vivo* is difficult owing to dilution of the damaged DNA by nascent DNA, saturation of the repair system by a high dose of carcinogen, biphasic removal of adducts etc. A rapid simple method has recently been developed (Craddock *et al.* (1982), *Biochim. Biophys. Res. Comm.*, **107**, 546) for determining the ability of tissue extracts to remove the methyl group from $O^6$MB-DNA. This method has been used to assess the importance of repair in carcinogenesis by comparing repair ability and rate of cell replication in animal tissues which have very different susceptibilities to cancer induction by a low dose of NMU, i.e. rat and mouse liver, spleen, thymus, lung and brain. As susceptibility in the rate varies very markedly with age, the repair capacity of rat liver and brain at different stages of foetal and post-natal development has been measured. The results support the view that it is essential to consider the effect of the carcinogen on cell replication as well as the extent and persistence of $O^6$MG in DNA.

### Quantitation of $O^6$-methyldeoxyguanosine and $O^2$-methylthymidine in cellular DNA using monoclonal antibodies

C.P. Wild, R. Saffhill & J.M. Boyle

*Paterson Laboratories, Christie Hospital and Holt Radium Institute, Manchester M20 9BX, U.K.*

Mouse hybridomas producing monoclonal antibodies to the putative promutagenic lesion $O^6$-methyldeoxyguanosine $O^6$-MedG) and $O^2$-methylthymidine ($O^2$-MedT) have been characterised by radioimmune assays (RIA). In competitive RIA, 50% inhibition of binding of anti-$O^6$-MedG and anti-$O^2$-MedT to the respective [$^3$H]-antigens was obtained using 0.3 pmol of cold $O^6$-MedG and 0.5 pmol of $O^2$-MedT respectively. Similar assays using normal nucleotides and the major methylation product, N-7 methylguanine in tests of cross reactivity showed that $>10^6$ and $>10^5$ higher concentrations of these compounds were required to cause the same inhibition of binding of anti-$O^6$-MedG and anti-$O^2$-MedT. The $O^6$-MedG and $O^2$-MedT antibodies have affinity constants of $3\times10^9\,l\,mol^{-1}$.

A technique has been developed which allows 5–25 $\mu$g clean DNA to be obtained from $1–5\times10^6$ cells by extraction on polycarbonate filters. The DNA can be completely enzyme hydrolysed and the nucleosides separated by Aminex A6 chromatography thus allowing the spectrophotometric quantitation of normal nucleosides and RIA of the alkylated nucleosides. Using this technique we have determined the percentage of $O^6$-MedG removed in 24 h by fibroblasts of human lung tumours, MRC-5 (60%), and Chinese hamster cell lines, V79A2 (10%) and V79/79 (55%) using doses of N-methyl-N-nitrosourea as low as 0.5 mM.

### DNA repair enzymes in human cell lines sensitive ($Mex^-$) and resistant ($Mex^+$) to nitrosoureas

A.L. Harris[1], P. Karran, B. Demple & T. Lindahl

[1]*CRC Unit, Royal Victoria Infirmary, Newcastle upon Tyne and ICRF, Mill Hill, London*

Human cell lines differ in their rate of removal of a specific alkylation lesion from DNA—$O^6$ methyl guanine ($O^6$MeG) (Sklar and Strauss (1981), *Nature*, **289**). $Mex^-$ cell lines (slow removal rate) are more sensitive to methyl nitrosourea (MNU), CCNU and BCNU than $Mex^+$ cell lines. In *E. Coli* exposure to MNU induces a specific suicide enzyme, $O^6$ MeG transferase ($O^6$MeGt), that removes the methyl group from $O^6$MeG, leaving the base intact. We have detected a similar enzyme in human cell lines, measured its activity in 2 $mex^+$ and 2 $mex^-$ cell lines and the activity of 4 glycosylases. Enzymes were extracted from 1–5 g of cells and partially purified by AcA54 chromatography. $O^6$MeGt activity was measured after acid hydrolysis and HPLC of [$^3$H] MNU alkylated DNA substrate. 3Me guanine, 3Me adenine and 7Me guanine glycosylase were measured by HPLC of supernatants after incubation with [$^3$H] DMS labelled DNA. Uracil glycosylase was measured by counting uracil (U) released from [$^3$H]U DNA.

No $O^6$MeGt was detectable in $mex^-$ cell lines, even after partial purification. Both $mex^+$ cell lines had similar levels of $O^6$MeGt. Thus, $mex^-$ cell lines have at least 100× less enzyme than $mex^+$. Mixing experiments showed no evidence of inhibitors. There was no difference in glycosylases in the 4 cell lines (Raji, GM1953, GM621, GM829). These results show lack of a specific enzyme is correlated with sensitivity to nitrosoureas. These enzyme assays will be applied to samples from human tumours in patients treated with nitrosoureas.

### Metabolism and carcinogenicity of alkylated polycyclic hydrocarbons

M.R. Osborne and P. Brookes

*Institute of Cancer Research, London, U.K.*

We have examined the metabolism by rat liver microsomes of a number of carcinogenic and non-carcinogenic polycyclic hydrocarbons, in order to determine how alkyl-groups affect the metabolism and carcinogenic potency; all 12 monomethylbenzanthracenes and 6 alkylated benzopyrenes. The presence of an alkyl-group affected metabolism not only at the bond to which it was attached, but also at neighbouring sites. For example, metabolism at the K-region (5,6-) of benzanthracene was inhibited by methyl-groups at the 4-, 5-, 6- or 8-positions. The carcinogenic potency of the hydrocarbons could only partly be explained in terms of their pattern of metabolism. Some methylbenzanthracenes were capable of yielding 3, 4-dihydrodiols, but were not carcinogenic; 1-isopropylbenzo(a)pyrene could be metabolised on the 7, 8, 9, 10-ring but was also inactive. The diolepoxides derived from these hydrocarbons are probably unreactive owing to electronic and steric factors, respectively.

### Variance in resistance to NK cell-mediated lysis among K562 lines: Influence of sodium n-butyrate on sensitivity

I. Kimber & M. Moore

*Department of Immunology, Paterson Laboratories, Christie Hospital and Holt Radium Institute, Manchester M20 9BX*

Mammalian natural killer (NK) cells have the capacity to bind to, and lyse, a variety of malignant and non-malignant cells in short term culture. Although the nature of the effector cells has been extensively studied the characteristics which determine resistance or susceptibility to NK-mediated lysis remain unclear.

To facilitate investigation of the factors which predispose cells to lysis we have isolated sub-populations of the erythroleukaemic cell line K562 by limiting dilution techniques. The characteristics of two such sub-lines, E10/P2 and F9/P2, which differ markedly in their susceptibility to NK cells have been examined in detail. We have demonstrated that such differences are not attributable to the level of expression of NK cell receptor sites and are probably secondary variable membrane repair capacity following immunological lesions.

We report that treatment of the resistant line (F9/P2) with the differentiating agent sodium n-butyrate induces a significant increase in lytic sensitivity within 48 hours, which is apparently independent of enhanced NK cell receptor expression. Similar treatment of the sensitive line (E10/P2) fails to increase susceptibility. These data suggest that the sensititivity of target cells to immune lysis is influenced by their level of differentiation.

### Tumour growth inhibitory activity of short term *in vitro* cultured lymph node cells (LNC) derived from mice pre-treated with or bearing a non-immunogenic tumour

K.D. Chandradasa & J. Blears

*Department of Immunology, University of Liverpool, P.O. Box 147, Liverpool L69 3BX*

Tumour inhibitory activity was detected in short term cultured LNC from mice bearing a "non-immunogenic", naturally arisen tumour-SP/N-1 (Chandradasa & Blears (1982a), *Eur. J. Cancer and Clin. Oncol.*, **18**, 1063; (1982b), 853). We investigated this phenomenon further in a different naturally arisen Balb/c tumour-SP/T-1, which also failed to elicit a detectable antitumour response in the isogenic host. Tumour pre-treated mice failed to suppress ($10^4$ tumour cells) a cell dose that forms tumour in nearly 100% of the inoculated animals, when tested by tumour challenge as well as by cell transfer assays. When LNC from mice repeatedly inoculated with Mitomycin C (MMC) treated tumour cells were cultured *in vitro* for 45 h, the resulting cells acquired the ability to suppress the tumour outgrowth in cell transfer assays, quite in contrast to the ineffectiveness of non-cultured LNC from similarly pre-treated donors. Cultured LNC obtained from tumour-bearers were also inhibitory although less so than those obtained from tumour pre-treated animals. Non-cultured cells obtained from normal, tumour bearing or tumour pre-treated animals as well as cultured normal LNC failed to show tumour inhibitory activity. These observations indicate that *in vivo* suppression of effector cell differentiation may be the reason for "non-immunogenicity" of the tumour systems we have investigated.

### Preliminary investigating on the mode of action of CCRG 81010 (M & B 39565)

C. Horgan, M.F.G. Stevens & M.J. Tisdale

*CRC Experimental Chemotherapy Group, Department of Pharmacy; University of Aston in Birmingham, B4 7ET, U.K.*

The new antitumour drug CCRG 81010 (or M & B 39565) is an unstable molecule which can potentially liberate a cascade of reactive and cytotoxic species *in vivo*. Two obvious degradation pathways could be expected: (i) formation of 2-chloroethylisocyanate and 5-diazoimidazole-4-carboxamide and (ii) ring-opening leading to formation of 5-[3-(2-chloroethyl)triazen-1-yl]imidazole-4-carboxamide (MCTIC).

Recent results (Gibson & Hickman (1982), *Biochem. Pharmacol.* **31**, 2795) have suggested that the TLX5 lymphoma cells may be sensitive to BCNU *in vivo* as a consequence of the intracellular release of 2-chloroethylisocyanate. On the other hand chloroethylnitrosoureas are generally considered to exert their activity (against L1210) *via* the alkylation and cross-linking of DNA. We were interested to examine the *in vitro* effects of CCRG 81010 in comparison with BCNU and MCTIC in the hope that they might shed light on the mode of action of the new drug. Under conditions where BCNU produced a marked inhibition of glutathione reductase in TLX5 lymphoma cells CCRG 81010 was without effect. Similarly CCRG 81010 proved to have no effect against $\gamma$-glutamyltranspeptidase and $\alpha$-chymotrypsin in direct drug-enzyme inhibition studies. BCNU caused a large decrease in incorporation of (methyl-$^3$H) thymidine and (5-$^3$H) uridine into acid-precipitable material in TLX5 cells *in vitro*, but an equi-cytotoxic concentration of CCRG 81010 had little early effect. The biochemical evidence suggests that 2-chloroethylisocyanate release is not an important factor in the mode of action of CCRG 81010 and the parameters investigated thus far point to its having similar mechanism of action to MCTIC.

### Uptake and retention of *cis*-platin and *cis*-diammine-1,1-cyclobutane dicarboxylate platinum II (CBDCA, JM8) in rat erythrocytes

Z.H. Siddik, D.R. Newell, F.E. Boxall, K.G. McGhee & K.R. Harrap

*Dept. Biochemical Pharmacology, Inst. Cancer Res., Sutton, Surrey, England*

In rats, death from *cis*-platin is usually ascribed to either renal failure, gastrointenstinal toxicity or both. As yet, evidence of pathological lesions which would afford a plausible explanation for CBDCA-induced lethalities is lacking. Recent studies in our laboratory indicate that toxicity to the erythrocytes by CBDCA may provide an explanation for the lethal event. Intravenous administration of CBDCA ($80\,mg\,kg^{-1}$) to rats results in a 70% decline in the erythrocyte count by day 9, and death ensues a day later. In contrast, lethalities from *cis*-platin ($10\,mg\,kg^{-1}$) are preceded by haemoconcentration. Atomic absorption spectrophotometric analyses of whole blood and plasma demonstrated that in animals receiving CBDCA, the Pt concentration in the erythrocytes is twice that seen in rats given *cis*-platin. Similarly, whole blood-plasma Pt ratios 4 days after administration were about three-fold greater for CBDCA than *cis*-platin (14 *vs*. 4). Taken together, these results indicate that the accumulation of Pt in the erythrocytes of rats treated with CBDCA is significantly greater than in animals given *cis*-platin. Parallel studies *in vitro* with rat whole blood resulted in similar findings, with *cis*-platin ($30\,\mu M$) and CBDCA ($300\,\mu M$) giving erythrocyte/plasma Pt ratios at 24 h post incubation of 1.5 and 3.4 respectively. The greater intracellular accumulation of Pt from CBDCA appears to be due to its faster rate of irreversible binding in the erythrocyte than in the plasma. These results are consistent with the premise that deaths from CBDCA may be associated with erythrocyte toxicity.

**Modulation of the acceptance of transfer RNA for amino acids by alkyl aryltriazines and imidazoles**

G.F. Kolar & J. Hradec

*Institute for Toxicology and Chemotherapy, German Cancer Research Centere, Heidelberg, F.R.G.; Department of Biochemistry, Oncological Institute, Prague, Czechoslovakia*

Selected triazenes and imidazoles were incubated with postmitochondrial supernatants from rat liver, and tRNA isolated therefrom was charged with 13 different amino acids. The majority of tested compounds enhanced the acceptance of initiator tRNA but inhibited the formation of L-leucyl-tRNA. The direct alkylating monomethyltriazenes enhanced the acceptance of initiator tRNA when incubated alone with unfractionated tRNA whereas the procarcinogenic dialkyltriazenes showed this effect only after preincubation with postmitochondrial supernatant containing the activating enzymes. It appears that the reactive molecular species [$CH_3$—N≡$N^+$, $CH_3$—N=N—OH], that arise from monomethyltriazenes by heterolysis, or from dimethyltriazenes by enzymic activation, modify the structure of tRNA in a specific manner, possibly by methylation. These results are in complete agreement with previous experience with carcinogenic and noncarcinogenic polycyclic hydrocarbons and azo dyes. Moreover, they provide additional supporting evidence that the enhanced acceptance of initiator tRNA seems to be indicative for the generation of electrophilic (potentially carcinogenic) intermediates from the incubated compounds.

***In vivo* metabolism of adriamycin in the rat: Identification of new metabolites**

J. Cummings[1], N. Wilmott[2], J.F.B. Stuart[1,2] & K.C. Calman[1]

[1]*Department of Clinical Oncology, University of Glasgow, Glasgow and* [2]*Department of Pharmaceutics, University of Strathclyde, Glasgow*

As part of a continuing study of adriamycin (ADR) metabolism in different tissues, with regard to cardiac toxicity and anti-tumour effect, we have examined *in vivo* metabolism of the drug in selected rat tissue. Previous reports of the disposition of ADR by this species have found little evidence for its biotransformation. In this present work rats were given ADR 10 mg $kg^{-1}$ i.v. and then killed sequentially with time. Blood, heart, lungs, liver, kidneys and a subcutaneously growing Mc 40A tumour were collected; the tissues and tumour were immediately frozen in solid $CO_2$ and all were stored at −20°C ready for analysis. ADR and its metabolites were extracted from homogenised tissues and blood with chloroform:isopropanol (2:1) by a new method which uses a Buchler Vortex Evaporator. Extracts were analysed by high pressure liquid chromatography (HPLC) and thin layer chromatography (TLC). Kinetic behaviour of ADR and metabolite profiles varied from tissue to tissue. For example in liver ADR and 3 metabolites which accounted for >50% of the ADR were eliminated very rapidly in an almost identical fashion to the plasma. Whereas in the tumour the ADR level peaked after four hours and fell very slowly, only one metabolite was detected and this was present in only very small amounts. Two metabolites previously reported not to be present in the rat were identified by chromatographic mobility to be adriamycinol 7-deoxy-aglycone and adriamycin 7-deoxyglycone. These *in vivo* products were isolated from the tissue extracts using 1 mm preparative TLC plates, their chemical identity established by rechromatographing against pure standards and verified by mass spectroscopy. The new data is clear evidence for extensive metabolism of ADR by the rat. Qualitatively the pathways of metabolism are the same as other species examined, including man, although quantitative differences exist.

**Increased thymidylate synthetase activity in L1210 cells resistant to CB3717**

A.L. Jackman, D.L. Alison, A.H. Calvert & K.R. Harrap

*Department of Biochemical Pharmacology, Institute of Cancer Research, Sutton, Surrey*

Defining mechanisms of resistance to an antimetabolite may be of use in the elucidation of its locus of action and may allow the development of improved analogues which are not cross-resistant. In addition, if resistance is due to over production of the target enzyme then it should be possible to purify large quantities of the enzyme for a number of studies such as sequencing the primary structure and affinity-labelling the active site. Thymidylate synthetase (TS) is the rate-limiting enzyme in the *de novo* synthesis of thymidylate. In the absence of salvageable thymidine a cell is completely dependent on its *de novo* thymidylate pathway for DNA synthesis. Cytotoxicity of the pyrimidine analogues 5-fluorouracil and 5-fluorodeoxyuridine requires the presence of the activating enzymes phosphoribosyl transferase and thymidine kinase respectively, and resistance is frequently determined by deletion of these enzymes and not by an increase in the target enzyme TS. A small increase in TS (6–10 fold) has been achieved in a hepatoma cell line although resistance was lost in the absence of drug (Priest *et al.* (1980), *Biochem. Pharm.*, **29**, 1549–1553). CB3717 is a quinazoline analogue of folic acid whose sole cytotoxic locus is TS (Ki = 4 nM) and which does not require metabolic activation. In the present communication resistance to CB3717 in L1210 cells was raised by passaging cells in a culture medium containing negligible amounts of salvageable thymidine, with incremental concentrations of the drug. Resistance was slow to develop initially but eventually the cells became completely resistant to 500 $\mu$M CB3717 ($ID_{50}$ for sensitive line = 5 $\mu$M). Several monoclonal lines have been isolated and all share the common feature of raised TS ($\cong$30 fold). The mutation appears to be stable in the absence of CB3717 (>6 months). There is no cross-resistance to 5-fluorouracil, 5-fluorodeoxyuridine or methotrexate. We have purified TS from one of the resistant cell lines by affinity chromatography and have shown that the kinetic constants and inhibition by CB3717 is the same as that for the sensitive line. This suggests that the gene product is identical. Future work will show whether gene amplification is the mechanism of resistance.

### Studies on the potentiation of melphalan toxicity with prednisolone in the rat

K.G. McGhee, D.R. Newell & K.R. Harrap

*Department of Biochemical Pharmacology, Inst. Cancer Res., Sutton, Surrey, England*

Melphalan (M) and Prednisolone (P) are used in combination for the treatment of a number of human tumours. The toxicity of M and P combinations has been studied in male and female non tumour-bearing rats and in male tumour-bearing (Walker 256) animals. The haematological toxicity and lethality of i.v. M was increased in some cases by the simultaneous administration of P at 10 mg kg$^{-1}$ i.p., as shown below:

| | *Melphalan* | | *Melphalan + Prednisolone* | |
|---|---|---|---|---|
| *Rat* | *LD50** | *WBC nadir†* | *LD50** | *WBC nadir†* |
| Male | 3.7 ± 1.4 | 25% | 5.7 | 20% |
| Male tumour bearing | 11.3 | N.D. | 3.2 ± 0.9 | N.D. |
| Female | 10.1 | 50% | 5.6 | 23% |

*mg kg$^{-1}$ melphalan i.v. ± 95% confidence limits where available.

†Effect of 2 mg kg$^{-1}$ melphalan i.v. as % control.
N.D.—not determined.

In female rats and in tumour-bearing male rats the increase in M toxicity produced by P was maximal when the two drugs were administered simultaneously. Furthermore, the P-induced increases in M (4 mg kg$^{-1}$ i.v.) haematological and gastrointestinal toxicity were dose-dependent over the range 0.1–100 mg kg$^{-1}$ P. As combinations of M and P were no more effective than M alone against the Walker 256 carcinosarcoma the therapeutic index of M was reduced by its combination with P. Hence against human disease, if the combination of M and P is not more effective than M alone, the concurrent use of the two drugs should be avoided. The mechanism of the P-induced increases in M toxicity in rats is unknown. However, a pharmacokinetic interaction has been excluded as P did not alter M plasma levels or protein binding in either male or female non tumour-bearing rats.

### Dimethylnitrosamine-induced changes in rat liver plasma membrane fluidity affects glucagon-stimulated adenylate cyclase activity

G.P. Margison, A.D. Whetton, N.J.F. Dodd, L. Needham & M.D. Houslay

*Paterson Labs., Christie Hospital, Manchester, U.K. and Biochemistry Department, UMIST, Manchester.*

Dimethylnitrosamine (DMN) can act as a total carcinogen in the liver. This ability may involve processes complementary to the well-documented interaction of dialkylnitrosamine metabolites with DNA. We have explored the effects of DMN on the important regulatory enzyme of glucagon-stimulated cyclase (GSAC) which mediates the hormone-stimulated vectorial flow of information into the liver cell. DMN inhibited the activity of this enzyme by up to 45% in the uncoupled state and also inhibited the hormone-stimulated state after initially activiting the enzyme. Maximal effects were observed at a DMN concentration of 15 mM. In an effort to understand the nature of this inhibition the effect of DMN on the fluidity of isolated rat liver plasma membranes was studied by electron spin resonance using a spin labelled fatty acid probe. This revealed that DMN produced a marked rigidification of the plasma membrane which was dose dependent with a maximal alteration in the fluidity occurring at a concentration of 20 mM. The increase in membrane rigidity can be demonstrated to be the cause of the observed inhibition of GSAC. The structurally similar non-carcinogenic DMN analog dimethylamine hydrochloride showed no effect on membrane fluidity, but slightly activated GSAC. These findings indicate that DMN, via its effects on membrane fluidity could influence plasma membrane function and cellular response to external stimuli. It is possible that *in vivo* this property of DMN may promote the development of cells that have been initiated by DNA alkylation.

### Removal of $O^6$-methylguanine from DNA by human, monkey and rat tissue extracts

J. Hall, H. Brésil & R. Montesano

*Unit of Mechanisms of Carcinogenesis, International Agency for Research on Cancer, Lyon, France*

$O^6$-Methylguanine ($O^6$-MeG), formed in DNA by reactive intermediates of various alkylating agents, has been shown to be a promutagenic lesion, and its persistence in the DNA of tissues correlates with the probability that that tissue will develop tumours after administration of alkylating agents. In *in vitro* assays, using methylated DNA as the substrate, human, monkey and rat liver extracts were shown to be able to catalyse the removal of $O^6$-MeG. The removal was specific for $O^6$-Meg, since 7-methylguanine and 3-methyladenine present in the DNA substrate were not removed by the tissue extracts. The amount of removal was proportional to the amount of protein added and the loss of $O^6$-MeG occurred with stoichiometric formation of S-methylcysteine within the protein. It was found that human and monkey liver have similar levels of activity (0.8 mg of protein of both enzyme extracts removed approximately 80% of the $O^6$-MeG from the DNA substrate) and that human liver was about 6 times more active than rat liver. A similar activity has also been found in monkey colon but of a lower activity compared with monkey liver, 0.8 mg of liver protein removed approximately 80% of the $O^6$-meG from the DNA substrate compared with approximately 40% removal by the same quantity of colon extract protein. The comparison between the removal activity in other monkey tissue extracts and human tissue extracts will be discussed.

### Expression of a human osteogenic sarcoma antigen on mitogen-stimulated human peripheral blood mononuclear cells

M.R. Price, D.G. Campbell & R.W. Baldwin

*Cancer Research Campaign Laboratories, University of Nottingham, Nottingham NG7 2RD*

In initial tests, the murine monoclonal antibody 79IT/36 against a human osteogenic sarcoma exerted complement dependent cytotoxicity against human peripheral blood mononuclear cells stimulated with PHA. By flow cytofluorimetry using a FACS IV Cell Sorter, antibody reactivity was directed against a subpopulation of cells judged to be PHA-blasts by their forward angle light scatter properties. After lactoperoxidase catalysed radio-iodination of cells and detergent lysis, immune complexes are isolated following the addition of 79IT/36 antibody and Sepharose-Protein A. The apparent molecular weight of the labelled antigen was determined by SDS PAGE and autoradiography to be 72,000, which is equivalent to that of the surface antigen, p72, precipitated by 79IT/36 antibody from lysates of human osteogenic sarcoma cells. This antigen is thus distinct from transferrin receptors which display similar expression in mitogen stimulated T-lymphocytes and tumour cells and consequently it may represent an additional cell surface marker for proliferating human cells.

### Growth of human colorectal carcinomas *in vitro*

S.C. Kirkland & N.A. Wright

*Department of Histopathology, RPMS, Hammersmith Hospital, Ducane Road, London W12 0HS*

In order to investigate growth control in human colorectal carcinoma cells, we have established monolayer cultures of these cells.

Forty-seven human colorectal carcinomas, of various histological types were collected at operation. Cells were mechanically released from tumour pieces and plated into culture flasks in Dulbecco's modified Eagles medium containing 10% foetal calf serum, kanamycin (100 $\mu g\,ml^{-1}$), amphotericin B (1.25 $\mu g\,ml^{-1}$) and minocycline (1 $\mu g\,ml^{-1}$), Gentamicin (100 $\mu g\,ml^{-1}$) and penicillin (50 $\mu g\,ml^{-1}$) were added to this medium for the initial culture period.

Eleven tumours were lost with bacterial contamination, while cells from 20 of the tumours failed to attach to the plastic culture flasks and rapidly degenerated. Sixteen tumours yielded cells which attached to plastic culture flasks and were of epithelial-like morphology, 8 of these showed long term proliferation (2–23 months). To date, cell lines have been established from 3 primary adenocarcinomas.

When injected subcutaneously into nude mice, these cell lines form tumours whose histology closely resembles that of the original tumour. Two of the 3 cells lines secrete carcinoembryonic antigen (CEA). Confluent cultures of the mucinous adenocarcinoma produce "domes" previously observed in densely confluent cultures derived from a variety of transporting epithelia.

These cell lines will be used to test the effects of a variety of hormones and growth factors on the proliferation of human colorectal carcinoma cells *in vitro*.

### The study of two morphologically distinct lines obtained from a metastasizing mammary adenocarcinoma

S.C. Barnett & S.A. Eccles

*Institute of Cancer Research, Clifton Avenue, Sutton, Surrey SM2 5PX*

The biological properties of cells from a murine mammary adenocarcinoma of recent origin were studied during serial transplantation in syngeneic CBA/Ca hosts as the tumour progressed from a well-differentiated, poorly metastatic neoplasm to an anaplastic highly metastatic state. At early generations the tumours yielded uniform cultures of cuboidal epithelial cells which grew in polygonal clusters. By p17, both epithelial and less cohesive spindle type cells were obtained, and by p27 the tumours yielded only spindle cells. However, the original cultures of cuboidal epithelial cells did not spontaneously alter their morphology during a corresponding period *in vitro*. Early passage tumours, and parental and cloned epithelioid cell lines gave only pulmonary colonies after i.v. inoculation, in contrast to late passage tumours and spindle cell lines which showed extensive extrapulmonary colonisation, especially in liver and lymph nodes. The ability of the latter cells to traverse pulmonary capillary beds was confirmed using radiolabelled cells, and similar patterns of dissemination and growth were seen in spontaneous metastasis assays. In spite of the marked phenotypic differences in these "subpopulations" some evidence points to their being of the same cell lineage since their electron microscopical features, estrogen receptor levels, and lectin binding profiles were closely comparable. Cloned lines of epithelioid cells have been converted to spindle type morphology by the addition of DMSO in culture. This phenotypic modulation has remained stable so far for 8 subsequent passages in DMSO free medium and their biological properties are being compared with the original cell lines.

### Experimental analysis of factors affecting distribution of metastatic tumour colonies

S.F. Juacaba, J.E. Price, K.M. Potter & D. Tarin

*Nuffield Department of Pathology, (University of Oxford), John Radcliffe Hospital, Oxford*

Disaggregated cells from a large series of murine mammary tumours have been inoculated intravenously and intra-arterially to study both the distribution of tumour colonies formed and the distribution of labelled cells in the body after vascular release. It has been found that when cells of spontaneous murine mammary carcinomas are injected intravenously the deposits are virtually confined to the lungs and this accords with the finding in animals where the tumours have spread spontaneously. When the cells are inoculated into the aorta it is observed that they are capable of colonising other organs and that the individual tumours have reproducible preferences for establishing colonies in certain sites while consistently not forming deposits in others. Using a new technique involving labelling of the mammary tumour cells with fluorescein isothiocyanate it has been shown that they reach all organs examined within 15 min and failure of a tumour to colonise a particular site is, therefore, not due to its cells not arriving there.

It is concluded that the distribution of metastatic colonies is influenced mainly by interplay between intrinsic properties of the tumour cells and microenvironmental influences in the organs where they arrest.

**Temperature-dependent elaboration of collagenase by the renal adenocarcinoma of the leopard frog, *rana pipiens***

D.J. Ogilvie, R.G. McKinnell & D. Tarin

*Nuffield Department of Pathology, (University of Oxford), John Radcliffe Hospital, Oxford*

Spontaneous renal adenocarcinomas in North-American leopard frogs metastasise frequently (77%) when these poikilothermic animals are kept in a warm environment but not when they are kept cold (Lucké and Schlumberger (1949), *J. Exp. Med.*, **89**, 269). This provides an opportunity for identifying factors which associate specifically with metastatic behaviour. We have found that explants of these tumours secrete collagenase, an enzyme capable of dissolving connective tissue fibres and previously found to be closely correlated with metastatic colony-forming capability of murine mammary tumours (Tarin, Hoyt & Evans (1982), *Br. J. Cancer*, **46**, 266), and that the amount released sequentially rises and falls as the ambient temperature is shifted between metastasis-permissive and inhibitory levels. In contrast, *normal* frog renal tissue has low collagenase output, unaffected by temperature changes. Glucose utilisation and lactate production are similar for normal and neoplastic tissue at both temperatures, demonstrating that differences in enzyme output are not simply due to raised metabolic rate, nor to shifts in the glycolytic pathway. The output of another tumour-associated protease (plasminogen activator) by normal and neoplastic frog renal tissue shows no significant association with temperature. Hence, collagenase output by Lucké carcinoma tissue is specifically associated with metastasis-permissive conditions, but further experiments are needed to test whether the relationship is causal. The peculiar temperature-related properties of this tumour make it potentially suitable for studying the genetic regulation of the metastatic phenotype.

**Characterization of clonogenic cells from human tumour xenografts by centrifugal elutriation and flow cytometry**

T.C. Stephens, A.C. Jones, J.H. Peacock & P. Wilson

*Radiotherapy Research Unit, Institute of Cancer Research, Sutton, Surrey, U.K.*

A range of human tumour xenografts of different histological types (pancreatic, ovarian and lung carcinomata, and melanomas) are being disaggregated with collagenase/pronase/DNase, and the cells quantitatively separated using a Beckman centrifugal elutriator, to yield fractions with increasing peak cell volumes. Each fraction is then tested for clonogenic potential in soft-agar, cytological preparations are made, and samples of cells are stained with ethidium bromide for examination by flow cytometry. The objective is to identify and characterize cells with high-clonogenic potential *in vitro* and then to explore whether such cells might equate with tumour stem cells *in vivo*.

For most of the tumours examined so far, we have successfully identified and separated murine host cells, but have been unable to identify a discrete high cloning tumour cell fraction. We have instead found that the elutriation technique yields a good separation according to cell cycle position and that a uniformly high cloning efficiency is observed at all phases of the cell cycle. However, in a lung adenocarcinoma which was difficult to disaggregate, colony formation was associated only with clumps of cells, perhaps indicating the unwillingness of these cells to grow in isolation. All the tumours so far tested have been poorly differentiated, and we are now looking at more differentiated tumours, and developing density gradient techniques to complement elutriation for cell separation.

**Radioimmunoprecipitation of an antigen defined by an anti human osteogenic sarcoma antibody**

D.G. Campbell, M.R. Price & R.W. Baldwin

*Cancer Research Campaign Laboratories, University of Nottingham, Nottingham NG7 2RD*

The monoclonal antibody 791T/36 is known to cross react with cells other than the immunising 791T osteogenic sarcoma cell line. On 791T cells the 791T/36 epitope is expressed on a protein with an apparent molecular weight of 72,000. A preliminary investigation was performed to determine whether the epitope occurred on similar molecules on other cell lines. After surface labelling various cell lines by lactoperoxidase catalysed radioiodination and preparing detergent lysates, immune complexes were isolated by the addition of 791T/36 antibody and Sepharose-Protein A. The radiolabelled antigen was then characterised by SDS-PAGE and autoradiography. The immune precipitates from three osteogenic sarcoma cell lines (2 OS, 788T and 278T), the prostate carcinoma EB33T and the colon carcinoma HcLo—all previously shown to be reactive with 791T/36 antibody—each contained a protein with a molecular weight of 72,000 as the only or major constituent. Cell lines not cross reactive with the antibody did not contain any antigen as detectable by the methods used. Neuraminidase treatment of the immune precipitates from all the osteogenic sarcoma cells tested caused a reduction in apparent molecular weight from 72,000 to 55,000, implying that the 791T/36 antigen is a glycoprotein with a high sialic acid content.

### Can the differences in metastatic potential of B16 murine melanomas be explained in terms of their glucocorticoid receptor status?

G.P. Risely & G.V. Sherbet

*Cancer Research Unit, University of Newcastle upon Tyne, Royal Victoria Infirmary, Newcastle upon Tyne NE1 4LP*

We have shown that the BL6 variant of the B16 murine melanoma, when grown as a subcutaneous tumour in C57BL/6 mice, has a high level of glucocorticoid receptor (GR), This differs from the F10 variant (very low), F1 (negative) and the parental B16 melanoma (negative). It also differs with respect to inducibility of tyrosine amino transferase, effects of dexamethasone on growth of cells in tissue culture, expression of cell surface proteins and the content of cell surface sulphydryl groups. It seems likely that these effects are receptor-mediated, which is compatible with the differences in GR status of the various lines. This may also explain why, in our hands, the BL6 tumour is found to be metastatic, while the parental type, F10 and F1 are nonmetastatic.

### Stimulation of ovarian tumour cell clonogenicity by mesothelial cells

A. Wilson

*Department of Obstetrics & Gynaecology, Withington Hospital, Manchester, U.K.*

Mesothelial cells (MC) derived from the ascitic fluids of patients with ovarian cancer have been used as feeder cells in the base of a double-layer agar system for determining the clonogenicity of ovarian tumour cells (T). Using an ovarian tumour cell line it has been shown that the degree of stimulation obtained is dependent on the ratio of MC:T. Maximum stimulation occurred at a ratio of 10MC:IT and 100MC:IT, with plating efficiencies of 14% and 16% respectively in the presence of MC compared with 0.78% and 1.7% in their absence. MC only were not clonogenic. Although MC have been found to produce Pg $F_2\alpha$, indomethacin did not abolish the feeder effect. Treatment with 1 $\mu g ml^{-1}$ of cisplatinum did not abolish the colony-stimulating activity of these cells. Use of 5% $O_2$ instead of 20% $O_2$ increased the P.E. of the cell line but MC were still stimulatory in low $O_2$. Seven primary ovarian tumours have been plated in the presence of MC and with five tumours clonogenicity increased from 2- to 5-fold. In view of the low frequency of ovarian cancer metastases outside the abdominal cavity where mesothelial cells are abundant it is possible that these cells have an important role in the regulation of proliferation of ovarian cancer.

### The effects of growth inhibitory agents on differentiated and malignancy-associated properties in astrocytoma cells

M.C. Frame & R.I. Freshney

*Department of Clinical Oncology, University of Glasgow, Glasgow*

Biochemical marker properties have been chosen to represent the differentiated and malignancy-associated astroglial phenotypes and assay systems developed to investigate their levels of expression in monolayer cell cultures derived from anaplastic astrocytomas (grades III and IV, Kernohan and Sayre histological grading). Post-confluent cultures were exposed to a variety of drugs, whose effects were investigated by assaying treated cells for the biochemical marker properties.

Many of the agents studied altered the balance between the expression of differentiated and malignancy-associated properties in astrocytoma cells. In particular dexamethasone, a crude extract from pig brain, and interferon "pushed" the phenotypic expression of the malignant astrocytes in the direction of more mature, differentiated astroglia; at the same time expression of malignancy-associated properties was reduced. Dexamethasone and pig brain extract in combination showed enhanced activity in altering cell phenotype over that caused by either agent alone. The tumour promoting phorbol ester, TPA, effectively "pushed" the phenotypic balance in the direction of malignancy, as determined by the *in*

*vitro* criteria; the differentiating properties were unaffected by this agent. The DNA-alkylating carcinogens, mitomycin C and methylnitrosourea, stimulated expression of both differentiated and malignancy-associated cell properties.

The possible relevance of these findings in considering the growth and spread of tumours after chemotherapy and new treatment procedures for malignant disease will be discussed.

### Toxicity and pharmacokinetic studies with CB3717

D.R. Newell, Z.H. Siddik, A.L. Jackman, P.M. O'Connor, K.G. McGhee, M. Radacic, A.H. Calvert & K.R. Harrap

*Department of Biochemical Pharmacology, Inst. Cancer Res., Sutton, Surrey, England*

CB3717 is a folate-based inhibitor of thymidylate synthetase (TS). Preclinical toxicity studies in mice identified renal toxicity as the dose-limiting side effect whilst in early clinical studies hepatic and haematological toxicities have been observed. Experiments have been performed in rats and mice to determine the dose and schedule-dependency of these effects. Renal toxicity, as indicated by CB3717 precipitation and an increase in kidney wet weight was observed in rats receiving $>800\,mg\,m^{-2}$ given as a 2 h i.v. infusion and at $330\,mg\,m^{-2}$ given i.v. daily $\times 5$. These doses are greater than those currently used in man. Hepatic toxicity, as indicated by elevations in plasma alanine transaminase could not be demonstrated in mice or rats following doses of less than $800\,mg\,m^{-2}$. Haematological toxicity was not observed in rats with CB3717 given either as a 2 h infusion at $1\,g\,m^{-2}$ or daily $\times 5$ at $330\,mg\,m^{-2}$. In contrast, methotrexate ($10\,mg\,m^{-2}$ $\times 5$) produced leucopenia (70% control) and a depression in the red blood cell count (60% control). Thus, rats are relatively resistant to the haematological and hepatic toxicities of CB3717. Experiments with $^{14}C$-CB3717 in mice have demonstrated that following $260\,mg\,m^{-2}$ i.p. 46% of the dose is excreted in the faeces and 20% in the urine within 48 h. The material present in the urine is predominantly unchanged CB3717 (77%). Analysis of faecal material indicated the presence of unchanged CB3717 (57%) and the desglutamyl metabolite (18%). *In vitro*, the hydrolysis of CB3717 to this metabolite can be catalysed by the intestinal flora of mice. The desglutamyl metabolite of CB3717 is not observed in the plasma of mice or rats and is not an inhibitor of TS and, thus, probably does not contribute to the activity or toxicity of CB3717.

### Flow cytometric analysis of DNA distribution in Lewis lung carcinoma cells after treatment with CCRG 81010 (M & B 39565)

C. Horgan[1], M.J. Tisdale[1], E. Erban[2], M. D'Incalci[2] & S. Pepe[2]

[1]*CRC Experimental Chemotherapy Group, Department of Pharmacy, University of Aston in Birmingham and* [2]*Institute di Richerche Farmacologische Mario Negri, Milan*

Flow cytometric studies of DNA distribution allows the determination of the number of cells in the $G_1$, S and $G_2/M$ compartments of the cell cycle. The aim of this study was to compare the effects of 8-carbamoyl-3-(2-chloroethyl)-imidazo[5,1-d]-1,2,3,5-tetrazin-4(3H)-one (CCRG 81010; M & B 39565) and 5-[3-(2-chloroethyl)triazen-1-yl]imidazole-4-carboxamide (MCTIC), a potential metabonate of the drug, on cell cycle progression in 3 LL cells. For the initial *in vitro* study logarithmically growing cultures of 3 LL cells, prepared from a primary animal tumour were treated with equimolar concentrations of the two agents. Flow cytometry was performed 24 h after drug treatment and again after a further 24 h recovery period in drug free media. Both drugs at a concentration which gave minimal cytotoxicity and little depression of [$^3$H]TdR incorporation produced a marked cytotoxicity effect. Specifically the $G_1$ fraction decreased whereas the late $S/G_2M$ fraction increased, a perturbation that was more pronounced after the 24 h recovery period. In the complementary *in vivo* study on CCRG 81010 a single dose of $20\,mg\,kg^{-1}$ (i.p.) to $C_{57}B_{1/6}$ mice bearing 10 day old 3 LL carcinoma produced the same cytokinetic effect. Determinations were made 24, 48 and 96 h after treatment, there being a progressive increase in the extent of the $G_1$ to LS-$G_2$-M shift and at 96 h no tumour cells were detected. The antitumour effect was evaluated by general autopsy on day 21 after transplantation at this time there was a 37% decrease in the weight of the primary tumour and nearly complete inhibition of metastases. The results obtained show that CCRG 81010 is producing a block in the $G_2/M$ region of the cell cycle in marked similarity to the effects of MCTIC.

### Analysis of the cytokinetic effects of a bifunctional alkylating agent in differentially sensitive human cell lines using flow cytometry

S.W. Dean and M. Fox

*Paterson Laboratories, Christie Hospital & Holt Radium Institute, Manchester, M20 9BX, U.K.*

The cytokinetic effects of nitrogen mustard on human lymphoblasts differentially sensitive to the cytotoxic effects have been studied using Flow Cytometry. A dose dependent S-phase delay (up to 48 h at the highest dose used) was observed in both cell lines. The delay was greater in the more sensitive cell line for a given drug concentration.

Fanconi's anaemia fibroblasts are more sensitive to the cytotoxic effects of HN2 than normal human fibroblasts. The latter showed an S-phase delay similar to that seen in lymphoblast cell lines. Fanconi's anaemia fibroblasts however, showed no such delay in response to treatment.

Incorporation of [$^3$H]-thymidine was measured for up to 128 h after treatment of the lymphoblasts with HN2. Incorporation was seen to fall to $<20\%$ of control levels by 12 h in both cell lines and in the more resistant cell line remained below this level for up to 128 h.

We therefore suggest that DNA synthesis and cell cycle traverse are controlled by different mechanisms and that incorporation of [$^3$H]-thymidine may not be a true reflection of DNA synthetic activity in these cell lines.

### The species dependent pharmacokinetics of DTIC

C.J. Rutty, D.R. Newell, R.B. Vincent, G. Abel, P.M. Goddard, S.J. Harland & A.H. Calvert

*Department of Biochemical Pharmacology, Inst. Cancer Res., Sutton, Surrey, England*

5-(3, 3-dimethyl-1-triazeno)imidazole-4-carboxamide (DTIC) is a drug used in the treatment of malignant melanoma but with very limited therapeutic benefit. DTIC is known to undergo oxidative N-demethylation to give 5-(3-monomethyl-1-triazeno)imidazole-4-carboxamide (MIC), which then undergoes rapid chemical decomposition yielding a methyl carbonium ion which can methylate DNA. Thus the antitumour activity of DTIC is dependent upon metabolism of the drug. In view of the marked species differences in the N-demethylation of pentamethylmelamine (PMM) described earlier (Rutty *et al.* (1982), *Cancer Chem. Pharmacol.*, **8**, 105) the metabolism of DTIC in mouse, rat and man was examined. DTIC and its N-demethylated metabolites, MIC and 5-amino-imidazole-4-carboxamide (AIC) were measured by high performance liquid chromatography following treatment with $40\,\text{mg}\,\text{kg}^{-1}$ DTIC intravenously (i.v.) (rats and mice) or 1200 mg total dose as an intravenous infusion (patients). The plasma half-life ($t\frac{1}{2}$) of DTIC was significantly greater in the rat (35.3 min) than in the mouse (12.7 min), and greater still in two patients studied to date (74.5 and 90 min). Of greater significance were the peak levels of MIC found in the plasma of mice (47.5 $\mu$M) which were some 10 times higher than in either rat (3.5 $\mu$M) or man (5.5–7.5 $\mu$M). Correspondingly, DTIC shows marked activity versus the mouse PC6 plasmacytoma when given i.v., but is without activity against the Walker 256 tumour grown in the rat. It is therefore tempting to suggest that the poor clinical activity of DTIC is at least in part due to the low level of metabolism to MIC in man. Furthermore, these findings also suggest that the administration of a dialkyl phenyl triazene to patients may lead to similar difficulties in terms of metabolism.

### Response of rat mammary and prostate tumours to treatment with a biodegradable slow-release formulation of the LH-RH analogue, ICI 118630

B.J.A. Furr, B.E. Valcaccia & F.G. Hutchinson

*ICI Pharmaceuticals Division, Alderley Park, Macclesfield, Cheshire, SK10 4TG*

A biodegradable formulation of the peptide analogue of LHRH, ICI 118630 (D-Ser(Bu$^t$)$^6$, AzaGly$^{10}$-LHRH), has been developed which will release active drug in animals over a period of at least 28 days. A single implant given every 28 days to rats with the androgen responsive, transplantable Dunning R3327 prostate tumour caused a highly significant reduction in tumour growth to values identical to those in castrated animals. Twenty eight days after the eighth implant the testes were around 10% the size of those in control animals and the accesory sex organ weights were at castrate values. Serum LH and testosterone were undetectable and FSH was $\sim 30\%$ of control values. Similar treatment of rats with measurable dimethylbenzanthracene-induced mammary tumours caused marked tumour regression. In an adjuvant setting, starting 30 days after the carcinogen before the tumours are palpable, monthly implants of the drug will delay appearance of tumours until at least one month after the final treatment is given. There were no discernable lesions at the site of implantation in any animal. This formulation, which produces excellent inhibition of tumour growth, should be more acceptable clinically than daily injections.

### Sensitization of EMT6 multicellular tumour spheroids to CCNU and melphalan by hypoxic pre-incubation with nitroimidazoles

P.R. Twentyman, P. Workman & K.A. Wright

*MRC Clinical Oncology and Radiotherapeutics Unit, MRC Centre, Hills Road, Cambridge, CB2 2QH*

We have previously demonstrated (Twentyman (1982), *Br. J. Cancer,* **45**, 565) that the growth delay induced in EMT6 spheroids by a range of cytotoxic drugs is enhanced when the spheroids are pretreated under hypoxic conditions with 5 mM misonidazole (MISO) for a period of 3–5 h. The studies have now been extended to MISO analogues differing in electron affinity ($E^1_7$) and octanol/water partition coefficient (P.C.). The 2-nitroimidazoles SR-2508 ($E^1_7 = -388$ mV, P.C. = 0.046) and Ro 07-1902 ($E^1_7 = -391$ mV, P.C. = 2.5) were equally effective as MISO ($E^1_7 = -389$ nV, P.C. = 0.43) in sensitizing to CCNU with 5 h hypoxic pre-incubation at 5 mM. However the 4-nitroimidazole AM-1 ($E^1_7 = -564$ mV, P.C. = 0.44) was ineffective as a sensitizer to CCNU in such a regime. It therefore appears that electron affinity is the predominant factor for sensitization to CCNU *in vitro* and that lipophilicity is much less important. This is the opposite conclusion to that which we have reached for tumour sensitization *in vivo* to CCNU (Workman & Twentyman (1982), *Br. J. Cancer,* **46**, 249). At a reduced concentration of 0.5 mM of MISO ($= 100 \mu g ml^{-1}$) little if any sensitization of spheroids to CCNU was seen with 16 h of hypoxic preincubation. This exposure approximates that produced in patients following a single dose of $3 g m^{-2}$ of MISO. Similarly no enhancement of CCNU response was produced by 16 h at $100 \mu g ml^{-1}$ hypoxic pre-incubation with either SR-2508 or Ro 07-1902. These sensitizer regimes did, however, considerably enhance the subsequent response of spheroids to melphalan. Some increased sensitivity to melphalan was produced by 16 h pre-incubation under hypoxia alone. The additional sensitization due to the presence of the nitroimidazoles again showed little dependence upon lipophilicity.

### The effects of retinoic acid analogues on the growth and metastases of murine sarcomas and carcinomas

S.A. Eccles

*Division of Tumour Immunology, Institute of Cancer Research, Sutton, Surrey*

A study was made of the effects of retinoids on the growth (*in vitro* and *in vivo*) and spontaneous metastasis of a variety of murine sarcomas and carcinomas. Tumour-bearing mice were dosed orally with $40 mg kg^{-1}$ Tigason Ro-9359 daily throughout tumour growth, and cell cultures were treated with $10^{-6}$ and $10^{-8}$ M concentrations of the same retinoid.

Certain of the tumours studied (notably immunogenic sarcomas and carcinomas) responded to retinoid treatment *in vivo* by slower growth rates and in some cases complete regression, whereas non-immunogenic tumours were unaffected. *In vitro*, retinoid treatments generally were without significant effect, and where any growth inhibition was seen it did not correlate with *in vivo* tumour responsiveness. The data suggested that in these systems the *in vivo* effects of retinoids on tumour growth were mediated indirectly, possibly in their role as immunopotentiators. This was further supported by experiments showing that immunosuppression abolished the retinoid inhibition of tumour growth, and prior immunisation enhanced the effect. Ro-9359 administration during tumour growth did not significantly inhibit the subsequent development of spontaneous metastases of non-immunogenic tumours, but decreased the incidence of secondary disease of tumours of moderate immunogenicity. It was also found that retinoid treatment inhibited the induction of tumour metastasis caused by whole body X-irradiation, but not that by Cyclosporin A. These and other data suggest that the retinoid effects observed were due to stimulation of host anti-tumour effector cells, possibly mononuclear phagocytes.

### Effects of single dose misonidazole on the pharmacokinetics of CCNU in mice

F. Lee & P. Workman

*MRC Clinical Oncology and Radiotherapeutics Unit, MRC Centre, Hills Road, Cambridge, CB2 2QH*

Using a high performance liquid chromatography (HPLC) method we have investigated the effect of misonidazole (MISO) on the pharmacokinetics of CCNU in mice. CCNU and MISO were given at a dose of 20 mg $kg^{-1}$ i.p. and 500 mg $kg^{-1}$ i.p. respectively. In the absence of MISO the plasma clearance kinetics of CCNU were biphasic—the $t\frac{1}{2}\alpha$ was 2.3 min and the $t\frac{1}{2}\beta$ was 53 min. MISO given 30 min before CCNU prolonged the $t\frac{1}{2}\alpha$ by a factor of 2.6 but had no effect on $t\frac{1}{2}\beta$. Moreover, the apparent volume of distribution was decreased by a factor of 1.6. As a result, the plasma area under the curve (AUC) was increased by a factor of 1.7. The AUC for the total active metabolites was increased by a similar amount. Further, the MISO dose-response relationship for pharmacokinetics changes was similar to that for chemosensitization. Studies with 4 tumours, the KHT, RIF-1 and EMT6 mouse tumours and the HT29 human tumour xenograft, showed that MISO raised the tumour concentrations of CCNU by 2–2.5 times. Detailed studies in the KHT tumour showed that there was a lag period before peak tumour CCNU concentrations were reached and that MISO increased the peak concentration by a factor of 2.4. In contrast, studies with critical normal tissues, the gut and the bone marrow, have shown that MISO did not increase the peak CCNU concentration in these tissues. This differential effect of MISO on peak CCNU concentrations of tumour and normal tissues is probably responsible for the enhancement of tumour toxicity and the therapeutic gain seen in *in vivo* chemosensitization experiments.

### Enteroglucagon neither promotes carcinogenesis nor stimulates colonic cell turnover in defunctioned rat large bowel

J.B. Bristol, M.A. Ghatei, S.R. Bloom & R.C.N. Williamson

*Department of Surgery, Bristol Royal Infirmary, and Department of Medicine, Royal Postgraduate Medical School, London*

Small bowel resection promotes adaptation and enhances carcinogenesis in rat colon (Williamson (1979), *Ann. Roy. Coll. Surg. Engl.*, **61**, 341). It is also associated with increased levels of plasma enteroglucagon and increased cell proliferation in small bowel sequestered from the faecal stream (Sagor *et al.* (1982), *Br. J. Surg.*, **69**, 14). The effect of luminal and systemic factors on colonic cell proliferation and carcinogenesis was studied in rats given either a defunctioning transverse colostomy ($n=34$) or sham colostomy ($n=15$) followed by 6 weekly injections of azoxymethane (AOM) 15 mg $kg^{-1}$ body weight. Rats with colostomy then underwent 85% jejunoileal resection (JIR) or sham resection; those with sham colostomy also had a sham JIR and acted as controls. Tumour yield and colonic crypt cell production rate (CCPR) were studied in the defunctioned segment or its equivalent 30 weeks later, when plasma enteroglucagon was estimated. Median tumour yield in defunctioned colon fell from 2 to 0 after colostomy ($P<0.05$) and was unaffected by JIR. CCPR in control rats was $5.6 \pm 0.4$ cells/crypt/hour, falling to $1.2 \pm 0.6$ after colostomy ($P<0.05$). It too was unaffected by subsequent JIR. Plasma enteroglucagon rose very slightly after colostomy ($97.1 \pm 17.9$ pmol $l^{-1}$ versus $127.5 \pm 19.1$ pmol $l^{-1}$, N.S.). After JIR plasma enteroglucagon doubled ($232.1 \pm 23.7$ pmol $l^{-1}$; $P<0.01$). Despite increases in plasma enteroglucagon after JIR, neither CCPR nor tumour yield was altered in defunctioned colon. Luminal factors must therefore predominate in maintaining cell turnover and promoting carcinogenesis in rat large bowel.

### Morphological changes occurring during prolonged organ culture of colonic mucosa from normal rats and from rats pretreated with dimethylhydrazine *in vivo*

P.V. Senior, J.P. Sunter, D.R. Appleton[1] & A.J. Watson

[1]*Departments of Pathology & Medical Statistics, University of Newcastle upon Tyne*

Rats treated with the carcinogen dimethylhydrazine (DMH) develop multiple colonic tumours preceded by a distinct phase of crypt hyperplasia. Using a development of a system which permits prolonged maintenance of murine and human mucosae in organ culture, we have studied the morphological changes in control and treated tissue for up to 25 days. Multiple explants were set up on cellulose acetate filters and maintained in Waymouth's medium supplemented with ascorbic acid, ferrous sulphate and hydrocortisone in 95% $O_2$ 5% $CO_2$ at 37°C and rocked at 8 cycles $min^{-1}$. Prior to culture treated mucosae taken at 4–8 weeks after treatment showed hyperplastic and dysplastic crypts. During the first 5 days in culture progressive loss of mucin from crypt cells together with crypt shortening was seen in both groups and displastic crypts were no longer apparent in the treated tissue. Ultrastructural preservation was good but intercellular spaces were enlarged. Some loss of crypts was seen possibly due to the initial trauma.

Crypts lined with a single layer of columnar cells were observed until the end of culture at 25 days; however in both control and treated tissue tortuous "adenomatous" crypts began to appear from about 10 days onwards. In the crypts of both groups mitotic activity was restricted to the basal two-thirds but mitoses were seen in the surface epithelium overlying areas of crypt loss. We conclude that prolonged maintenance of colonic tissue is possible even in a simple system such as this but that neoplastic transformation *in vitro* does not occur following carcinogen pretreatment *in vivo*.

### Carcinogenesis in defunctioned rat colon: The effect of bile acid irrigation

J.B. Rainey, J.B. Bristol & R.C.N. Williamson

*University Department of Surgery, Bristol Royal Infirmary, Bristol*

The presence of faeces and its composition are important in colorectal carcinogenesis (Campbell *et al.* (1975), *Cancer Res.*, **35**, 1365). Bile acids, in particular, have been implicated as cocarcinogens in animals and man (Reddy (1975), *Cancer*, **36**, 2401). In 40 male Sprague–Dawley rats, a long segment of colon was isolated as a Thirty-Vella fistula (TVF). Twenty-five TVFs were irrigated 3 x weekly for 12 weeks with a 0.12 M solution of the secondary bile acid sodium deoxycholate (SDC). The remaining TVFs, ($n=15$), were not irrigated. Other rats underwent colonic transection and reanastomosis (sham TVF, $n=15$). Operations were performed one week after a course of azoxymethane (total dose $90\,mg\,kg^{-1}$ i.p.). Results at sacrifice (22 weeks):

| | *No. of colonic tumours (mean x s.e.m.)* | *% tumours in left colon* | *Tumour diam (mm) (mean ± s.e.m.)* |
|---|---|---|---|
| Sham TVF | 4.3 ± 0.6 | 81 | 4.7 ± 0.3 |
| TVF alone | 2.6 ± 0.3* | 51 | 4.4 ± 0.6 |
| TVF + SDC | 2.6 ± 0.4* | 58 | 2.6 ± 0.4** |

*$P<0.05$ *vs* shams.
**$P<0.01$ *vs* other groups.

Carcinogenesis was reduced in defunctioned colon probably because of mucosal atrophy and the normal left sided preponderance of tumour yield was abolished. SDC irrigation did not affect the TVF tumour yield and indeed resulted in smaller tumours. This unexpected protective effect may reflect a detergent role in cleansing the TVF. Alternatively, bile acids may exert their suggested cocarcinogenic effect only in the presence of faeces, anaerobic bacteria, or other, as yet unidentified, faecal constitutents.

### The role of faecal bile acids (FBA) in large bowel carcinogenesis

M.J. Hill, B.C. Morson & M.H. Thompson

*PHLS-CAMR, Porton Down, Salisbury and St. Mark's Hospital, London EC1*

Large bowel carcinogenesis is a multi-stage process involving adenoma formation, adenoma growth and the development of increasingly severe dysplasia and finally neoplasia. There is a strong correlation between the risk of large bowel carcinogenesis and FBA concentration; this correlation reflects the risk associated with high fat/high protein diets and is further enhanced by the observation that *in vitro* faecal organisms (termed NDC) possess the ability to degrade bile acids to carcinogen-like precursers. In our case-control studies 70% of 120 bowel cancer cases had elevated FBA concentrations and carriage rates of NDC compared with 10% of 116 patients with non-malignant bowel disease; the FBA/NDC discriminant was very much better with early than with advanced metastatic cancers, and was also better for tumours of the left than of the right colon.

In a study of 150 patients with colorectal adenomas there was a significant correlation between FBA concentration and adenoma size and the severity of their dysplasia, indicating that bile acids may be directly involved in promotion of the precurser state. In a study of 105 patients with ulcerative colitis of more than 10 years duration, those who developed severe epithelial dysplasia and carcinoma of the colon had higher FBA concentrations than those who did not, indicating that the bile acids might also be important in the carcinogenesis in these patients.

### Goblet cell changes during intestinal adaptation to azoxymethane and enteric bypass

J.B. Bristol, I.O. Olubuyide & R.C.N. Williamson

*University Department of Surgery, Bristol Royal Infirmary*

Jejunoileal bypass (JIB) promotes the development of experimental colorectal cancer, probably by enhancing mucosal cell turnover (Bristol *et al.* (1982), *Proc. Am. Assoc. Cancer Res.*, **23**, 60). Chemical carcinogenesis in rats is associated with increased numbers of goblet cells containing sialomucins (Filipe (1975), *Br. J. Cancer*, **32**, 60), and JIB itself may have a similar effect (Olubuyide *et al.* (1982), *Gut.*, **23**, A881). Adaptive changes, mucin histochemistry and tumour yields were therefore studied in male Sprague–Dawley rats ($n=45$) 30 weeks after 85% JIB (end-to-side) or sham bypass. Weekly s.c. Injections of azoxymethane were given (total dose $90\,mg\,kg^{-1}$), starting 6 weeks preoperatively. Controls had vehicle injections followed by sham bypass. Azoxymethane alone increased length and weight of duodenum and colorectum by 5–34% and colonic crypt depth by 10–15% ($P<0.05$). The number of goblet cells containing sulpho- and sialo-mucins was consistently increased throughout the intestine (by 14–40%). After JIB + azoxymethane, all values were further increased, despite a 27% reduction in body weight. Thus duodenal and colorectal length and weight were 16–212% greater, colonic crypts were 20–30% deeper and acid-mucin goblet cells were 6–40% commoner ($P=0.02$–$0.001$); maximal increments occurred in sialomucins in the distal colon. Moreover, JIB doubled the yield of large-bowel tumours (4.3 *versus* 2.2 per rat: $P<0.01$). The carcinogen (azoxymethane) and co-carcinogen (JIB) share the ability to cause intestinal hyperplasia, increasing in particular the number of colonic goblet cells that contain sialomucins.

### Comparative studies on the relative mutagenicities of Aflatoxin B$_1$-DNA adducts in a bacterial plasmid

S.C. Booth and R.C. Garner

*Cancer Research Unit, University of York, York YO1 5DD*

Aflatoxin $B_1$ ($AFB_1$), the major mycotoxin produced by certain strains of *Aspergillus flavus*, is a potent mutagen and animal carcinogen. Epidemiological evidence supports the concept that $AFB_1$ is also a human liver carcinogen. $AFB_1$ can be activated by mono-oxygenase enzymes or by peroxoacids to give $AFB_1$-8,9-oxide which can chemically react with nucleic acids to form as a major adduct 8,9-dihydro-8(7-guanyl)-9-hydroxy $AFB_1$. This reaction, which arises through electrophilic attack at the $N^7$-position of guanine, leads to an adduct which is unstable because of the protonated $N^7$-position. As a result the guanine adduct can give rise to (1) a guanine imidazole ring-opened form (iro $AFB_1$-DNA), (2) depurination through loss of the $AFB_1$-gua residue, and (3) loss of $AFB_1$-8,9-diol. Of these several pathways it is not known which are of biological importance. To try and answer this question we have compared the relative survival and mutagenicity of plasmid pK0482 which has been reacted with $AFB_1$ and then transformed into *E. coli* AB1886 ($uvrA^-$). Plasmid pK0482 carries both ampicillin resistance and GalK genes. Mutants in which the GalK gene has been inactivated appear white when grown on galactose/ampicillin MacConkey agar plates while normal transformants appear red. Ring-closed and ring-opened forms of the $AFB_1$-DNA adduct resulted in equal amounts of plasmid inactivation and mutation. To enable these results to be interpreted correctly, further experiments into the intra-cellular stability of ring-closed $AFB_1$-DNA and into the presence and mutagenicity of apurinic sites are being undertaken.

### Studies on the macromolecular binding of [3H-acetyl]benzidine and benzidine derived azo dyes *in vitro* and *in vivo*

J.C. Kennelly and C.N. Martin

*Cancer Research Unit, University of York*

Acetylation to produce *N*-acetylbenzidine (ABZ) is the first stage in activation of the human carcinogen benzidine (BZ). *N*-OH-diacetyl-benzidine (*N*-OH-DABZ) is the proximate carcinogenic species formed from ABZ in the presence of rat liver S9 *in vitro*. The activation of ABZ *in vivo* however proceeds along a different route. This study used rat liver slices to determine by DNA adduct analysis whether metabolism of ABZ in this system proceeded as *in vivo*. Overall binding to DNA was measured in hamster and rat liver at 24 and 168 h following IP injection of ABZ at $25\,mg\,Kg^{-1}$ (rat 70.8 and $27.8\,pmol\,mg^{-1}$ DNA, hamster 33.0 and $14.1\,pmol\,mg^{-1}$ at 24 and 168 h respectively) and in liver slices $100\,\mu g\,g^{-1}$ liver (rat 53.1, hamster $27.4\,pmol\,mg^{-1}$). Adduct analysis revealed the same adduct was present in each case, identified as *N*-(deoxyguanosin-8-yl)-*N'*-acetylbenzidine. The results suggest that, unlike S9 metabolism where sulfotransferase activity on *N*-OH-DABZ would lead to diacetylated DNA adducts, metabolism of ABZ in liver slices is similar to that occurring *in vivo*. Direct blue 6 is a potent rat hepatocarcinogen whereas congo red appears to be less potent. Both dyes are diazo derivatives of

BZ and are activated by reduction releasing the free amine. Both dyes are readily reduced by gut microflora but only Direct blue 6 is reduced by rat liver. Animals were gavaged or IP injected with [$^3$H] labelled dyes and liver DNA binding measured. In both cases Direct blue 6 bound to a greater extent implicating liver azo reductase activity in determination of carcinogenic potency.

### Covalent binding of ultimate carcinogens to glutathione transferase B *in vitro*

B. Coles[1], D. Meyer[1], B. Jernström[2], F. Beland[3] & B. Ketterer[1]

*[1]Courtauld Institute of Biochemistry, Middlesex Hospital Medical School, London, [2]Department of Forensic Medicine, Karolinska Institute, Stockholm and [3]National Center for Toxicological Research, Arkansas, U.S.A.*

Glutathione (GSH) transferase B was isolated because of its ability to react with a metabolite of N,N-dimethyl-4-aminoazobenzene (DAB) *in vivo*. Two of its properties concern us here: it is (1) a non-covalent binding protein for hydrophobic ligands and (2) an enzyme of detoxication. GSH transferase B might be expected to bind electrophilic metabolites of carcinogens initially by noncovalent forces and then either to react with the electrophile or catalyse its GSH conjugation. Three ultimate carcinogens were studied: +(anti)benzo(a)pyrene-7,8-diol-9,10-oxide (BPDE), N-sulphonyloxy-N-acetyl-2-aminofluorene (AAF-N-sulphate) and aflatoxin $B_1$-2,3-oxide ($AFB_1$-oxide). The first two were synthesized chemically and the last two biosynthetically by a microsomal system. BPDE and $AFB_1$-oxide do not react non-catalytically with GSH; AAF-N-sulphate reacts with GSH non-catalytically but is not a substrate for GSH transferase B. Covalent reaction has been studied with the two subunits which compose GSH transferase B; $Y_aY_c$. In the absence of GSH, BPDE reacted only with $Y_a$ (as does DAB *in vivo*) while AAF-N-sulphate and $AFB_1$-oxide reacted with both $Y_a$ and $Y_c$. In the presence of GSH, the resulting enzymic activity almost completely prevented the reaction of BPDE with the enzyme protein, but was only partially effective in preventing the reaction of $AFB_1$-oxide with the protein. Although AAF-N-sulphate was not a substrate for the enzyme, non-catalytic reaction with GSH was sufficient to cause substantial reduction in reaction with enzyme protein. These results show that GSH and GSH transferase B are potent in detoxifying BP but much less so for AFF or $AFB_1$. These properties may be important determinants in carcinogenesis e.g. $AFB_1$ and AAF are powerful hepatocarcinogens in the rat while BP is not.

### Studies on the fate of alfatoxin-8,9-epoxide

E.J. Moss, G.E. Neal and D.J. Judah

*Toxicology Unit, Medical Research Council Laboratories, Woodmansterne Road, Carshalton, Surrey, U.K.*

The reactive intermediate of the potent hepatotoxin and hepatocarcinogen alflatoxin $B_1$ ($AFB_1$) is believed to be the $AFB_1$-8,9-epoxide. The reaction of the epoxide with DNA is an important event which has received widespread attention. Alternative fates for the epoxide may include conjugation, hydrolysis to $AFB_1$-8,9-dihydrodiol, and protein interaction, possibly by electrophilic attack of the epoxide on nucleophilic sites in the protein or by the interaction of the ring opened form of the diol ($AFB_1$-dialdehyde) with the free amines of protein via Schiffs base formation.

We have recently observed that an important detoxification pathway for the epoxide involves formation of an S-linked glutathione conjugate involving glutathione-S-transferase B. The conjugate ($AFB_1$-GSH) has been identified by proton n.m.r. and mass spectral studies, and further enzymic degradation allowed the identification of other aflatoxin conjugates. The cysteinylglycine conjugate ($AFB_1$-Cys Gly) produced by $\gamma$-glutamultranspeptidase activity on $AFB_1$-GSH, was further degraded by dipeptidase. The material produced was identified as the cysteinyl conjugate ($AFB_1$-Cys) from proton n.m.r. data.

Analysis of these polar metabolites of $AFB_1$ by reverse phase HPLC has allowed investigations into their production by several systems. $AFB_1$-Cys has been investigated both as an expected degradation product of $AFB_1$-GSH *in vivo* and also as a possible marker to investigate the interaction of $AFB_1$-8,9-epoxide with protein sulphydryl groups.

### Sequential analysis of hyperplastic liver lesions induced by aflatoxin $B_1$ and partial hepatectomy, using GGT as a marker

M.M. Manson & G.E. Neal

*MRC Toxicology Unit, Carshalton, Surrey SM5 4EF*

Farber *et al.* (1977) (*Am. J. Path.*, **88**, 595), in their model for sequential analysis of liver carcinogenesis used an *in vivo* system involving a single carcinogenic dose of diethylnitrosamine, partial hepatectomy (PH) and short term dietary exposure to 2-acetyl-aminofluorene. We have examined a similar, but essentially simpler, system using only one carcinogen, Aflatoxin $B_1$, with particular reference to its effect on $\gamma$-glutamyl transpeptidase (GGT). Untreated or $AFB_1$-fed (4 ppm) male Fischer 344 rats underwent PH and were returned either to control or $AFB_1$ diet or received a single i.p. injection of $AFB_1$. GGT levels and distribution were compared in the same animal before and after PH. Animals fed $AFB_1$ long enough to develop GGT positive foci were much more resistant to a single i.p. injection of $AFB_1$ administered 24 h after PH. However, both untreated and fed animals survived well when returned to an $AFB_1$ diet after PH. GGT levels in untreated animals returned to control diet were elevated ($\times 2$) when examined 3 days or 1 week after PH, but only very slightly at 18 h. The increased enzyme activity was histochemically apparent in bile ducts and periportal hepatocytes. In $AFB_1$-fed animals the percentage increase in GGT 3 days or 1 week after PH increased with time on $AFB_1$ diet before the operation. GGT levels at 18 h post-PH were lower than before the operation in fed animals. Histochemistry showed that the size and number of foci increased after PH. We are using this system to examine the development of $AFB_1$-induced hepatocarcinogenesis and the relationship of GGT to this process.

### Monoclonal antibodies to normal hepatocytes reveal similarities in phenotypes expressed during development and hepatocarcinogenesis

C.H. Holmes, B. Gunn, E.B. Austin, A. Fisk, M.J. Embleton & R.W. Baldwin

*Cancer Research Laboratories, University of Nottingham, Nottingham NG7 2RD*

In an attempt to probe the cellular events associated with hepatocarcinogenesis we have produced a panel of monoclonal antibodies, 3 of which are directed against normal adult rat hepatocytes. We have used an immunohistochemical technique to examine the reactivity of these antibodies with normal adult liver, with developing foetal and neonatal liver and with azo-dye induced premalignant and malignant liver lesions including a range of 32 primary liver carcinomas. Monoclonal antibody RL24/72 stained hepatocytes and other hepatic parenchymal cells in frozen sections of normal adult liver. Staining with this antibody was detectable from 17 days gestation onwards. Most tumours showed some reactivity with RL24/72. Monoclonal antibody RL23/36 was specific for hepatocytes within the normal liver. The adult pattern of staining with this antibody was not observed until parturition. All tumours showed reduced staining with RL23/36 and many showed no reactivity. While monoclonal antibody RL16/79 was also specific for hepatocytes, only hepatocytes surrounding central veins were stained by this antibody, periportal hepatocytes were not. The adult staining pattern characteristic of this antibody was not established until 4 weeks *post partum*. Only 2 liver carcinomas were stained by RL16/79. The types of staining observed with these monoclonal antibodies in malignant and also in premalignant liver lesions indicate that re-expression of foetal phenotypes may occur during liver carcinogenesis.

### Monoclonal antibodies against B-cell non Hodgkin lymphomas (N.H.L.)

E.M. Rankin, A. Kekman, F.A. Vyth-Dreese, J.G. McVie & R. Somers

*The Netherlands Cancer Institute, Plesmanlaan 121, 1066 CX Amsterdam, The Netherlands*

We have identified 3 monoclonals with different degrees of specificity for malignant lymphomas. Lymphocyte cell membranes from the pleural effusion of a patient with centrocytic/centroblastic N.H.L. were used to immunise a Balb/c mouse, the spleen cells of which were fused with Sp 2/0 myeloma cells. Two monoclonals, M1 and M2, were obtained, both $IgG_1$. Both antibodies react with the immunising cells, but not with pooled human T and B lymphocytes, monocytes, erythrocytes, granulocytes, sections of thymus, spleen or lymph node, nor with the cell lines HSB, CEM, NALM-1, SB and U937, using enzyme-linked immunoabsorbent assay, indirect membrane immunofluorescence or immunoperoxidase staining on thin sections. There was no reaction between M1 and 13 lymphomas of different morphology. The M1 antibody did not react with cultured autologous T cells. In contrast to M1, M2 reacted with pooled immunoglobulins and free $\kappa$ light chains and the EBV-transformed B cell line JY. Immunofluorescence with M2 showed staining in 4 of 9 lymphomas. The reaction was not correlated with the presence of $\kappa$ light chain. Immunisation with cell membranes from the spleen of another centrocytic/centroblastic N.H.L. patient yielded one antibody L1. This reacts with the lymphocytes of the immunising patient but not with pooled T or B lymphocytes or U937 cells. In 3 cases of lymphoma, immunofluorescence showed that more than 50% cells were strongly positive, in 4 smaller numbers of cells were stained, and there was no reaction in 4 cases. There was no correlation between reactions with M1, M2 and L1 and known surface markers including immunoglobulin, $\kappa$ or $\lambda$ light chain, BA1 and BA2. M1 is highly specific for the immunising cells in contrast to M2 and L1, which may be useful in the elucidation of B cell differentiation pathways.

**Isolation and characterization of cytotoxic effector cells infiltrating spontaneous rat tumours**

B.L. Ferry, G.R. Flannery, R.A. Robins & R.W. Baldwin

*Cancer Research Campaign Laboratories, University of Nottingham, Nottingham NG7 2RD*

The proposition that natural killer (NK) cells mediate surveillance mechanisms *in vivo* against primary tumour development and metastatic tumour spread has recently received additional support (Warner & Dennert (1982), *Nature*, **300**, 31). In this respect, it might be expected that NK cells would be present in the infiltrates of primary and secondary tumours.

We have examined the infiltrates of primary and transplanted spontaneous rat tumours using immunohistology and separation on velocity sedimentation gradients. Of 12 spontaneous tumours, 9 yielded cytotoxic effector cells which lysed the rat myeloma Y3Ag in a 6 h chromium release test. (i) These effector cells were heterogeneous in size: all positive populations included cytotoxic small lymphocytes, but killing was also mediated in some tumours by larger cells, possibly cytotoxic macrophages. (ii) Cytolytic activity was generally lower than that of splenic NK cells and low yields correlated with histological patterns. (iii) *In situ* suppression is likely and both effector cell types responded *in vitro* to activation by interferon. (iv) Monoclonal antibody staining and positive selection by FACS indicated that most effector lymphocytes were OX-8 positive, in keeping with splenic NK cells (Cantrell *et al.* (1982), *Immunol.*, **45**, 97) but some experiments suggested that an OX-8 negative population was also present. Further heterogeneity is demonstrated by cell sorting experiments with W3/13 antibody where both positive and negative populations mediate killing. These data indicate that spontaneous tumours are infiltrated by cytotoxic effector cells which are heterogeneous and include various phenotypic subsets of NK cells.

***In vitro* activation of natural killer cell activity: Its possible relation to *in vivo* rejection of tumours using non-cytotoxic T cell subsets**

V. Britten, R.A. Robins & R.W. Baldwin

*Cancer Research Campaign Laboratories, Nottingham*

An assay has been developed to monitor *in vitro* boosting of rat natural killer (NK) cell activity using *in vivo* primed lymphocytes as a source of T "helper" cells.

This work is being done to identify *in vitro* correlates for *in vivo* experiments, done in our laboratory and elsewhere, which show that activated T "helper" cell subsets are important in the rejection of immunogenic tumours (or allografts) (Fernandez-Cruz *et al.* (1982), *J. Immunol.*, **128**, 1112; Loveland and McKenzie (1982), *Immunol.*, **46**, 1313) in adoptive transfer tests.

The T cell subpopulation necessary for this activity is reactive with W3/25 monoclonal antibody. This W3/25 positive subpopulation contains no cytotoxic T cells, NK cells, or their precursors. These cells may function by activation of other lymphocyte subsets at the tumour site, or by recruitment from other areas. As NK cells are known to be present in the rat tumours under study, (Ferry *et al.* (1983) *Br. J. Cancer*, **48**, 111) we wished to investigate whether tumour rejection might be induced by activation of NK cells by specifically triggered non-cytotoxic T cells.

T lymphocytes primed *in vivo* using BCG or tumour, were incubated in the presence of the appropriate antigen, with normal rat spleen cells as a source of NK precursors. Increased killing of NK targets could be demonstrated, when compared with the activity generated by activated lymphocytes incubated without the specific antigen or when normal lymphocytes were used as a source or "help".

### Native and inducible levels of natural cytotoxicity in lymph nodes draining human mammary carcinoma

I. Kimber[1], M. Moore[1], A. Howell[2] & M.J.S. Wilkinson[2]

[1]*Department of Immunology, Paterson Laboratories and* [2]*Department of Medical Oncology, Christie Hospital and Holt Radium Institute, Manchester M20 9BX*

Although suggestions that NK cells mediate innate host resistance to malignant development are attractive, it is difficult to reconcile such hypotheses with the reported paucity of natural cytoxic function exhibited by lymph node cells (LNC).

To further investigate the role of local NK cell function in malignancy we have examined the natural cytotoxic potential of lymphocytes isolated from axillary nodes draining mammary carcinoma.

Although less reactive than peripheral blood lymphocytes LNC possess variable natural cytotoxic capacity. Augmentation of LNC by interferon (IFN) is also variable with only some populations displaying potentiated lysis following exposure to either IFN-$\alpha$ or gene-cloned IFN-$\alpha_2$. Where present the IFN-induced augmentation of LNC cytotoxicity was invariably weaker than that observed following similar treatment of autochthonous peripheral blood mononuclear cells.

Irrespective of their responsiveness to IFN the cytotoxic capacity of all LNC preparations examined was significantly increased following pre-incubation with either Staphylococcal enterotoxin A (SEA) or factors elaborated by lectin-pulsed allogeneic LNC. The induction or amplification of LNC-mediated natural cytotoxicity by lymphokines may provide a local potentiation of natural immune function at the host : tumour interface.

### The relationship between lipid metabolism and immune depression in a tumour model

R. Windle

*Department of Surgery, Leicester Royal Infirmary, Leicester*

Serum triglyceride (TG) levels are raised in both human and experimental cancer (Dilman *et al.* (1981), *Br. J. Cancer,* **43,** 637). Fatty acids depress the mitogenic response of lymphocytes *in vitro* (Mertin and Hughes (1975), *Int. Archs. Allergy, Appl. Immun.,* **48,** 203) and a diet rich in polyunsaturated fats increases chemical carcinogenesis (Hopkins *et al.* (1978), *J. Natl Cancer Inst.,* **60,** 849). Therefore, it has been suggested by Dilman *et al.* that high TG levels may predispose to malignancy by causing a depression of host immunity. To investigate this hypothesis, TG and free fatty acid (FFA) levels have been measured during the growth of colonic tumours induced in rats by dimethylhydrazine and the results have been correlated with changes in cellular and humoral immunity.

Studies were performed at 8 weekly intervals after tumours appeared at 32 weeks in 4 groups of 12 rats. Cellular immunity was assessed by lymphoblast transformation in response to PHA using a whole blood method and humoral immunity was studied by measuring antibody titres to sheep red cells by agglutination.

Serum FFA levels became reduced at 40 weeks ($0.36\,mmol\,l^{-1}$ range 0.25–0.48 from $0.69\,mmol\,l^{-1}$ range 0.56–0.85). Serum TG levels became elevated at 48 weeks ($4.1\,mmol\,l^{-1}$ range 3.6–4.6 from $3.2\,mmol\,l^{-1}$ range 2.5–3.8).

Responsiveness to PHA became depressed at 40 weeks and antibody production was reduced at 56 weeks and these results were not affected by correction of the FFA and TG levels by heparin. It is concluded that in this tumour model, depression of immunity is not dependent upon changes in fat metabolism alone.